# Evaluating agreement between individual nutrition randomised controlled trials and cohort studies - a meta-epidemiological study

**DOI:** 10.1186/s12916-025-03860-2

**Published:** 2025-01-21

**Authors:** Julia Stadelmaier, Gina Bantle, Lea Gorenflo, Eva Kiesswetter, Adriani Nikolakopoulou, Lukas Schwingshackl

**Affiliations:** 1 Institute for Evidence in Medicine, Medical Center - University of Freiburg, Faculty of Medicine, University of Freiburg, Freiburg, Germany; 2 Cochrane Germany, Cochrane Germany Foundation, Freiburg, Germany; 3 Institute of Medical Biometry and Statistics, Faculty of Medicine and Medical Center, University of Freiburg, Freiburg, Germany; 4 Laboratory of Hygiene, Social and Preventive Medicine and Medical Statistics, School of Medicine, Aristotle University of Thessaloniki, Thessaloniki, Greece

**Keywords:** Meta-epidemiological study, Concordance, Randomised controlled trials, Cohort studies

## Abstract

**Background:**

In nutrition research, randomised controlled trials (RCTs) and cohort studies provide complementary evidence. This meta-epidemiological study aims to evaluate the agreement of effect estimates from individual nutrition RCTs and cohort studies investigating a highly similar research question and to investigate determinants of disagreement.

**Methods:**

MEDLINE, Epistemonikos, and the Cochrane Database of Systematic Reviews were searched from January 2010 to September 2021. We matched individual RCTs to cohort studies based on population, intervention/exposure, comparator, and outcome (PI/ECO) characteristics. Two reviewers independently extracted study characteristics and effect estimates and rated the risk of bias using RoB2 and ROBINS-E. Agreement of matched RCTs/cohort studies was analysed by pooling ratio of risk ratios (RRR) and difference of (standardised) mean differences (DSMD).

**Results:**

We included 64 RCT/cohort study pairs with 4,136,837 participants. Regarding PI/ECO similarity, 20.3% pairs were “more or less identical”, 71.9% “similar but not identical” and 7.8% “broadly similar”. Most RCTs were classified as “low risk of bias” (26.6%) or with “some concerns” (65.6%); cohort studies were mostly rated with “some concerns” (46.6%) or “high risk of bias” (47.9%), driven by inadequate control of important confounding factors. Effect estimates across RCTs and cohort studies were in high agreement (RRR 1.00 (95% CI 0.91–1.10, *n* = 54); and DSMD − 0.26 (95% CI − 0.87–0.35, *n* = 7)). In meta-regression analyses exploring determinants of disagreements, risk-of-bias judgements tend to have had more influence on the effect estimate than “PI/ECO similarity” degree.

**Conclusions:**

Effect estimates of nutrition RCTs and cohort studies were generally similar. Careful consideration and evaluation of PI/ECO characteristics and risk of bias is crucial for a trustworthy utilisation of evidence from RCTs and cohort studies.

**Supplementary Information:**

The online version contains supplementary material available at 10.1186/s12916-025-03860-2.

## Background

In nutrition research, randomised controlled trials (RCTs) and cohort studies provide complementary evidence, although neither study design is sufficient by itself to capture the whole picture of diet-disease relations [1, 2]. RCTs are deemed the gold standard and best suited for assessing the efficacy and safety of interventions and inferring causal relationships [3]. Nevertheless, their implementation is often fraught with ethical challenges and feasibility issues, particularly when investigating rare or long-term outcomes, or when study participants are likely to have strong preferences [4]. Cohort studies offer a counterbalance as they are non-experimental and follow groups of people in real-world settings and often over a long period of time. However, cohort studies are susceptible to bias, particularly from measurement error and unmeasured and residual confounding [5, 6], and are thus often considered as less trustworthy [5, 7].

Several meta-research studies of individual meta-analyses have evaluated how and to what extent effects from RCTs and observational studies differ when investigating a similar PI/ECO (population, intervention/exposure, comparison, outcome) question in medical research [8, 9], and recently also in the field of nutrition [10, 11]. On average, differences between RCTs and observational studies were small, but substantial statistical heterogeneity was detected at meta-epidemiological and individual meta-analysis levels. Differences in PI/ECO characteristics, including dose, sample size, and follow-up length, are proposed as potential determinants of heterogeneity and disagreement [10, 11].

Disagreement may also stem from flaws in the design or conduct of the individual studies, leading to biased effect estimates. A previous meta-epidemiological study on the influence of methodological study characteristics on effect estimates in nutrition RCTs showed that lack of blinding of outcome assessment and missing data may exaggerate intervention estimates [11]. In cohort studies, validity is threatened by known or unknown confounding variables that can distort the causal relationship between the dietary exposure and the outcome [5]. Inadequate evaluation and control of selection bias are thus a major source of biased effect estimates and varying conclusions about the diet-disease associations [12]. Additionally, measuring dietary exposure is challenging, and flaws in the method of assessment may contribute to bias due to misclassification [13].

Due to the tremendous role of an optimal diet in the prevention of non-communicable diseases [14], a comparison of individual RCTs and cohort studies is highly needed to further evaluate determinants of discordant effect estimates. This methodological approach allows best to explore the impact of PI/ECO matching criteria and risk of bias on the agreement of RCTs and cohort studies. To the best of our knowledge, no previous study has approached this before. Thus, this meta-epidemiological study aims to compare effect estimates of individual RCTs and cohort studies investigating a highly similar PI/ECO question and to investigate important determinants of disagreement.

## Methods

This meta-epidemiological study adheres to the Preferred Reporting Items for Systematic Reviews and Meta-Analyses (PRISMA 2020) [15] and guidelines for meta-epidemiological research [16].

### Data sources and searches

We searched MEDLINE, Epistemonikos and the Cochrane Database of Systematic reviews to identify nutrition systematic reviews, which provide evidence from RCTs and cohort studies for similar PI/ECO questions. Details of the search strategy have been reported previously [10, 11]. The two meta-epidemiological studies identified 183 eligible body of evidence-pairs. For the purpose of this study, a new sample was generated by excluding body of evidence-pairs that (i) evaluated different types of intervention/exposure (e.g. comparing vitamin C supplementation in RCTs vs. dietary vitamin C intake in cohort studies) or (ii) included only retrospective cohort studies. Eligibility criteria are described in Additional file 1: Appendix 1 [10, 11].

We accounted for overlaps between both meta-epidemiological studies, ensuring that PI/ECO questions are addressed only once. To achieve this, we prioritised the body of evidence-pairs identified in Stadelmaier 2024 [11], as the systematic reviews included encompassed both RCTs and cohort studies, thus sharing the same methodological approach to identify both study designs. For highly correlated outcomes (e.g. coronary heart disease and cardiovascular mortality), we selected the pair that included the largest number of studies from RCTs, or, in the second instance, the highest number of participants.

### Study selection

For each of the body of evidence-pairs included, we chose one individual RCT and one matching cohort study based on a standardised approach: First, we selected the RCT with the longest follow-up period. If multiple RCTs had the same follow-up period, the decision criterion was the largest sample size. Second, we matched the most similar (based on PI/ECO characteristics) cohort study to the RCT. A detailed description of matching guidance can be found in Additional file 1: Appendix 2 [10]. Matching was performed by two reviewers independently (JS, LS), and discrepancies were resolved by discussion.

### Data extraction

Data extraction was carried out independently by two reviewers (JS, GB) using a piloted data extraction form. Disagreements were solved by discussion with a third reviewer (LS).

For each included individual study, we extracted the following information: name of the first author, year of publication, country, study name (and acronym), study design (e.g. parallel or factorial RCT, prospective cohort or nested case–control studies), and the PI/ECO characteristics for the selected research question. The latter included information on the study population (e.g. mean age, disease status), intervention or exposure (e.g. Mediterranean dietary pattern, selenium supplementation), comparator (e.g. placebo, control diet, lowest intake category), type of comparison (e.g. high vs. low intake), and outcome (e.g. cardiovascular disease), as well as the duration of the intervention/exposure and the length of follow-up. Moreover, we extracted the sample size, number of participants, number of outcome events, comparison (e.g. highest vs. lowest intake category), type of effect size (e.g. risk ratios [RR], hazard ratios [HR], odds ratios [OR], mean differences [MD] or standardised mean difference [SMD]), and the effect estimates (and their corresponding 95% confidence interval [CI]).

### Evaluating similarity between RCTs and cohort studies

We rated PI/ECO similarity between each matched RCT/cohort study pair with a standardised approach, as described previously [10, 11], classifying the similarity of each PI/ECO domain as “more or less identical”, “similar but not identical”, or “broadly similar”. For instance, we rated pairs as “broadly similar” in population, when e.g. a population with an existing non-communicable disease (e.g. in the RCT) was compared to a general healthy population (e.g. in the cohort study). For study pairs with different interventions/ exposures of the same class – such as multivitamin supplementation in the RCT versus multi-micronutrient supplementation in the cohort study - we rated them as “similar, but not identical”. Additional details on the similarity rating, including examples, are provided in Additional file 1: Appendix 2 [10].

To determine the overall similarity of each study pair, the domain with the lowest degree of similarity was considered. For instance, if the domain “population” was rated as “broadly similar”, we rated the overall similarity of this study pair also as “broadly similar”. Two reviewers (JS, GB) independently assessed the PI/ECO similarity, and discrepancies were resolved through discussion.

### Assessment of risk of bias in included studies

Two reviewers (JS, GB, or LG) independently assessed the risk of bias of each included study with any disagreements resolved by discussion or involvement of a third author (LS). We used the revised Risk of Bias (RoB2) [17] and the Risk of Bias in Non-randomised Studies - of Exposure (ROBINS-E) tool [18] to assess the risk of bias in RCTs and cohort studies, respectively. If an individual study was included with several relevant outcomes, we conducted separate risk of bias assessments for each outcome. Moreover, multiple cohort studies included in the same publication were evaluated separately. The overall risk of bias for a study was judged as “low risk”, “some concerns”, or “high risk” (or “very high risk” in cohort studies). Assessments were visualised using Risk-of-bias VISualization (robvis) [19]. Additional guidance for the RoB2 and ROBINS-E assessments is provided in Additional file 1: Appendix 3 and 4.

### Data synthesis and analysis

Where necessary, we recalculated and/or converted effect estimates to improve the comparability between the RCT and its matching cohort study: Binary outcomes were expressed as RR. We standardised as recommended the direction of the effect for all studies, to ensure that binary effect estimates < 1 are expressing a beneficial effect [10]. If intake/supplementation dose differed between the RCT and its matching cohort study, we attempted to convert effect estimates to a standardised dose, using the RCT dose as the reference. We used the generalised least squares method described by Longnecker and Greenland 1992 [20] to estimate the RR for the dose used in the RCT. Continuous outcomes were presented as mean differences and converted, where necessary, to standard units (kg, mmol/l). Detailed descriptions of all transformations made are reported in Additional file 1: Appendix S5 and Table S1 [10, 20–99].

To quantify the comparison of effect estimates, we computed a ratio of risk ratios (RRR) [100] for each study pair with a binary outcome and a difference of mean differences (DMD) or standardised mean differences (DSMD) for continuous outcomes. This methodological approach is in line with previous meta-epidemiological studies in the field [8–11]. Cohort studies served as the reference group, so the summary effect estimates indicate whether effect estimates from the RCTs are larger or smaller compared to those in the matched cohort study. Of note, these measures do not indicate whether the effect is beneficial or harmful, as the direction of difference depends on the direction of effect of the underlying studies.

We used a random-effects model to pool the summary effect estimates (RRR, DMD or DSMD) and assessed the statistical heterogeneity of effect estimates with the *τ*^2^ or *I*^2^ statistics [101, 102]. To estimate *τ*^2^, we used the Paule and Mandel method [103, 104]. We computed 95% prediction intervals (PI) to provide the range of possible values for the differences between the results of RCTs and cohort studies, which are expected to arise in future studies comparing the two designs [105].

Subgroup analyses were conducted with respect to the different dietary interventions/exposures (dietary pattern, food groups or micronutrients), type of intake (dietary intake or supplementation), cluster of outcome (e.g. cardiovascular disease), risk of bias rating (e.g. pairs with low risk of bias in RCT and some concerns in matching cohort studies), and degree of PI/ECO similarity. We carried out three sensitivity analyses to assess the robustness of our findings. First, we evaluated whether excluding study pairs with at least one “high risk of bias” or “very high risk of bias” rating would alter the findings of the main analysis. Second, we accounted for overlapping individual RCTs. Third, in a post hoc analysis, we excluded study design pairs where the selected RCT was the longest but not the largest study (by sample size) in the respective systematic review.

We also performed univariable and multivariable meta-regression to explore the influence of PI/ECO similarity, RoB2 rating, or ROBINS-E rating as covariates on the summary effect estimate in our sample.

All statistical analyses were conducted using the R package meta (version 4.3.2) [106].

Besides the statistical analysis, we visually inspected the effect estimates of RCTs and cohort studies to examine the number and proportion of effect estimates that (i) point in similar or opposite directions; or (ii) show a significant difference in their RRR or DMD (95% CI does not overlap with the null-effect).

## Results

**Fig. 1:**
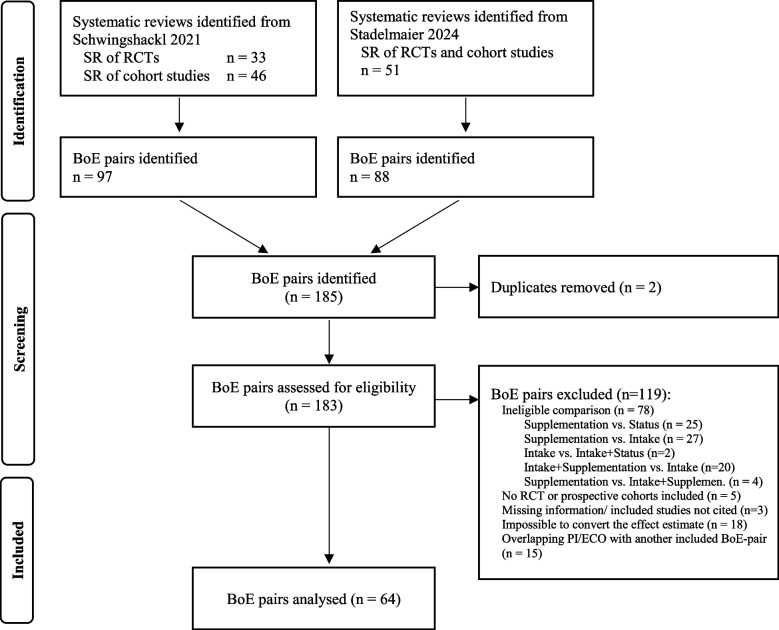
Flow diagram of the selection process. BoE, body of evidence; PI/ECO, population, intervention/exposure, comparator, outcome; RCT, randomised controlled trial; SR, systematic review

The flow diagram of the study search and selection process is displayed in Fig. 1. Of the 183 body of evidence-pairs identified in both meta-epidemiological studies [10, 11], we finally included 64 RCT/cohort study pairs from 45 systematic reviews [59, 60, 99, 107–148]. A list of all excluded body of evidence-pairs with their exclusion reasons is displayed in Additional file 1: Table S2 [59, 107–109, 114, 117, 120, 122, 125–127, 133, 140, 142, 144, 148–227]. After matching, our final sample comprised 35 individual RCTs and 31 individual cohort studies. In 50 out of the 64 pairs, the selected RCT with the longest follow-up also had the largest sample size among the studies within the respective body of evidence.

### Descriptive characteristics

Of the 35 RCTs (42 reports) [23–58, 228–233], most were conducted in the US (*n* = 16) and Europe (*n* = 14), and included participants at high risk for chronic disease (*n* = 16), or a general healthy population (*n* = 16). Of the 31 individual cohort studies (52 reports) [64–98, 234–250], most were conducted in the US (*n* = 16), and Europe (*n* = 10), including a general healthy population.

Out of the 64 included RCT/cohort study pairs, 40 (62.5%) investigated the effects of (micro-)nutrients (e.g. folic acid supplementation), 18 (28.1%) of dietary pattern (e.g. Mediterranean diet), and six (9.4%) of food groups (e.g. nut intake). The most common outcomes were cancer (*n* = 18, 28.1%), pregnancy-related outcomes (*n* = 11, 17.2%), cardiovascular disease (*n* = 10, 15.6%), and intermediate disease markers (*n* = 10, 15.6%). The total number of participants was 4,136,837, with a median of 2342 in RCTs and 29,361 in cohort studies. The study duration/ follow-up varied from 0.1 to 11.3 years (median: 4.1) in RCTs, and 0.3 to 30 years (median 7.4) in cohort studies. Detailed description of PI/ECO characteristics of each included primary study and matched study pairs are depicted in Additional file 1: Tables S3–S5 [23–61, 64–99, 107–111, 113–132, 134, 135, 137–148, 228–250].

### PI/ECO similarity degree

Thirteen (20.3%) study pairs were classified as “more or less identical”, 46 (71.9%) as “similar but not identical”, and five (7.8%) as “broadly similar” (Additional file 1: Table S6). The rating “broadly similar” was attributable to differences in study population, when participants at high risk or with chronic disease in RCTs [52, 230, 231] were compared to a general healthy population in cohort studies [93, 243, 244].

### Risk of bias

We classified 26.6% of RCTs as “low risk of bias”, 65.6% as “some concerns” and 7.8% as “high risk of bias” (Additional file 1: Fig. S1–S2). “High risk of bias” ratings were attributable to deviations from intended intervention (*n* = 1) or missing data (*n* = 4). Forty-two studies were found to have minor methodological flaws or did not adequately describe their methods with regard to the randomisation process (*n* = 28, 43.8%), missing data (*n* = 30, 46.9%), and/or the study protocol (*n* = 31, 48.4%), and were thus rated with “some concerns”.

Among cohort studies, 46.6% were rated with “some concerns”, 47.9% with “high risk of bias”, and 5.5% with “very high risk of bias” (Additional file 1: Fig. S3–S4). The high-risk ratings were mainly (28/35) attributable to non-measurement or inappropriateness of controlling for confounding factors. Four studies, reported solely unadjusted effect estimates. A detailed description of confounders considered by the cohort study authors are presented in Additional file 1: Table S7.

### Meta-epidemiological analysis

We analysed 54 study pairs with binary outcomes [23, 25–27, 29–33, 35–44, 46–49, 51–53, 55–58, 64, 65, 67–80, 83–87, 89–91, 93, 95–98, 228–250], and ten with continuous outcomes [24, 28, 30, 34, 45, 50, 54, 56, 66, 81, 82, 88, 92–95].

**Fig. 2:**
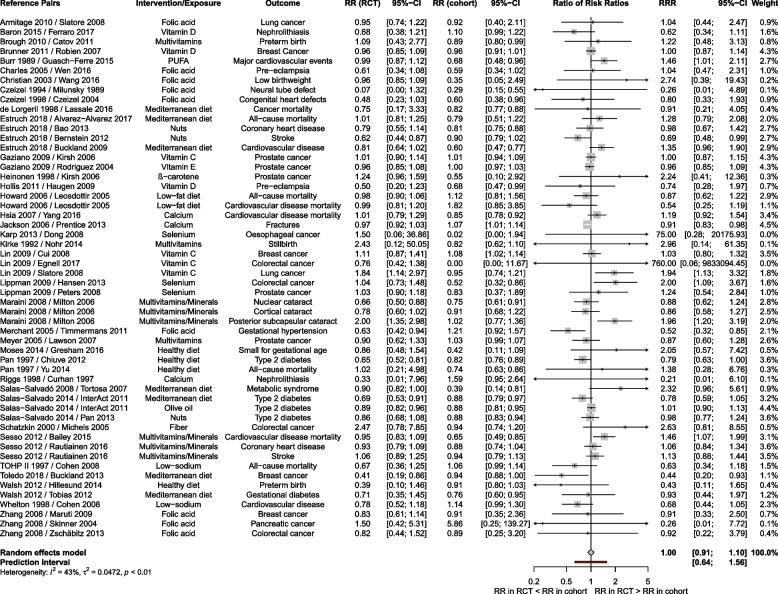
Forest plot of the overall comparison between pairs of randomised controlled trials and cohort studies for binary outcomes using pooled ratio of risk ratios. CI, confidence interval; RCT, randomised controlled trial; RR, risk ratio; RRR, ratio of risk ratios

The majority of RCTs (38/54, 70.3%) and cohort studies (40/54, 74.1%) showed a RR < 1. Comparing all matched study pairs, the effect estimates of RCTs and cohort studies were often in the same direction (32/54 for binary and 8/10 for continuous outcomes). Pooling RRR across matched study pairs showed high agreement (RRR 1.00, 95% CI 0.91 to 1.10, *I*^2^ = 43%, PI 0.64 to 1.56; see Fig. 2). In nine out of 54 (16.7%) pairs [27, 35, 41, 44, 46, 65, 75, 90, 91, 229, 230, 232, 233, 235, 237, 240, 244, 247], effect estimates differed between study designs (i.e. the 95% CIs did not include the null effect).

**Fig. 3:**
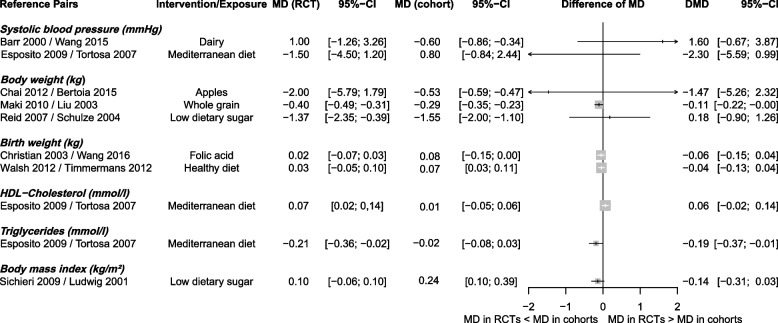
Forest plot of the comparison between pairs of randomised controlled trials and cohort studies for continuous outcomes using the difference of mean difference. CI, confidence interval; DMD, difference of mean differences; MD, mean difference; RCT, randomised controlled trial;

For continuous outcomes, DMDs between RCTs and cohort studies were small (Fig. 3). In two out of ten pairs [34, 45, 81, 93], effect estimates differed between study designs. The pooled DSMD was −0.26 (95% CI −0.87 to 0.35; *I*^2^ = 96%; PI −2.50 to 1.98, Additional file 1: Fig. S5).

### Subgroup, sensitivity, and meta-regression analyses

We conducted subgroup and sensitivity analyses for binary outcomes, but not for continuous outcomes since the number of eligible pairs was small.

Results of all subgroup analyses are depicted in Table 1 and in Additional file 1: Fig. S6–S10. Subgroup analyses by dietary intervention/exposure and type of intake (intake or supplementation) yielded agreement between study designs. Subgroup analysis by outcome type also showed no differences between study designs. When stratified by overall PI/ECO similarity, we observed on average no disagreement across pairs, but imprecise effect estimates and wide prediction intervals in the group of “broadly similar” pairs (RRR 1.36, 95% CI 0.85 to 2.15; PI 0.29 to 6.30, *n* = 5). Subgroup analysis by risk of bias revealed no disagreement between effect estimates across RCTs and cohort studies, but 95% CI and 95% PI were wide. Effect estimates were most dissimilar when comparing RCTs with “low risk of bias” to cohort studies with “some concerns” (RRR 1.27, 95% CI 0.99 to 1.64; PI 0.66 to 2.45, *n* = 6).

**Table 1:** Overview of main results for binary outcomes

	**Pairs included**	**Ratio of risk ratios** **(95% CI)**	**Heterogeneity** **(*I***^**2**^** (%); *τ***^**2**^**)**	**95% prediction interval**
Main analysis	54	1.00 (0.91 to 1.10)	43; 0.05	0.64 to 1.56
* Stratified by dietary intervention/exposure*				
(Micro-)nutrients	37	1.04 (0.93 to 1.17)	44; 0.05	0.65 to 1.65
Dietary pattern	13	0.93 (0.74 to 1.17)	46; 0.08	0.48 to 1.80
Foods group	4	0.95 (0.83 to 1.09)	23; 0.01	0.60 to 1.50
* Stratified by type of intake*				
Intake	21	0.94 (0.80 to 1.10)	48; 0.06	0.54 to 1.64
Supplementation	33	1.04 (0.93 to 1.16)	40; 0.04	0.70 to 1.56
Stratified by outcome				
Cancer	18	1.08 (0.89 to 1.31)	31; 0.06	0.61 to 1.91
Cardiovascular disease	10	1.06 (0.87 to 1.27)	59; 0.06	0.58 to 1.93
Pregnancy outcomes	11	0.79 (0.60 to 1.05)	1; 0.001	0.57 to 1.10
Endocrine/metabolic disease	7	0.90 (0.71 to 1.14)	50; 0.05	0.46 to 1.76
All-cause mortality	4	0.93 (0.70 to 1.24)	14; 0.01	0.42 to 2.04
Eye disease	3	1.12 (0.66 to 1.88)	76; 0.17	0.00 to 552.31
Fractures	1	0.91 (0.83 to 0.98)	NA	NA
* Stratified by overall PI/ECO similarity degree*				
More or less identical	10	1.06 (0.86 to 1.31)	41; 0.06	0.57 to 1.96
Similar but not identical	39	0.96 (0.87 to 1.06)	38; 0.03	0.67 to 1.38
Broadly similar	5	1.36 (0.85 to 2.15)	70; 0.18	0.29 to 6.30
* Stratified by risk-of-bias rating (rating in RCT vs. in cohort studies)*^a^				
Low risk vs. some concerns	6	1.27 (0.99 to 1.64)	56; 0.04	0.66 to 2.45
Low risk vs. high risk	10	1.00 (0.85 to 1.18)	36; 0.03	0.63 to 1.59
Some concerns vs. some concerns	19	0.98 (0.85 to 1.13)	47; 0.05	0.60 to 1.60
Some concerns vs. high risk	13	0.90 (0.68 to 1.19)	44; 0.10	0.43 to 1.91
Some concerns vs. very high risk	2	1.10 (0.11 to 10.52)	42; 1.18	NA
High risk vs. some concerns	1	0.87 (0.60 to 1.28)	NA	NA
High risk vs. high risk	2	0.98 (0.51 to 1.86)	0; 0	NA
High risk vs. very high risk	1	2.96 (0.14 to 61.35)	NA	NA

*CI* confidence interval, *NA* not applicable, *PI/ECO* population, intervention or exposure, comparator, outcome, *RCT* randomised controlled trial

^a^No study pairs were available for comparing low risk vs. low risk, low risk vs. very high risk, some concerns vs. low risk, and high risk vs. low risk

We identified 29 study pairs, where either the RCT and/or the cohort study were rated as (very) high risk of bias. Excluding these did not alter the findings of the main analysis (RRR 1.04, 95% CI 0.91 to 1.19, *I*^2^ = 50%, *τ*^2^ = 0.06, PI 0.62 to 1.75, *n* = 25, Additional file 1: Fig. S11). Similarly, the sensitivity analysis which accounted for overlapping RCTs confirmed the main findings (RRR 0.99, 95% CI 0.89 to 1.11, *I*^2^ = 42%, *τ*^2^ = 0.05, PI 0.63 to 1.56, *n* = 43, Additional file 1: Fig. S12). In the post hoc sensitivity analysis, excluding study pairs where the selected RCT was the longest but not the largest study in the respective systematic review, findings were similar to the main analysis (RRR 0.99, 95% CI 0.90 to 1.09, *I*^2^ = 40%, *τ*^2^ = 0.04, PI 0.66 to 1.48, *n* = 44, Additional file 1: Fig. S13).

Meta-regression analyses did not show any statistically significant effects of the potential determinants “PI/ECO similarity”, “RoB2 rating”, and “ROBINS-E rating” on the summary effect estimate in our sample (Additional file 1: Tables S8–S11). However, a multivariable meta-regression incorporating all three as covariates suggests that risk of bias judgements tend to have had more influence on the effect estimate in our sample than the “PI/ECO similarity” degree. For one-level increase in the “RoB2 rating” or “ROBINS-E rating” (reflecting increased susceptibility to potential sources of bias), the RRR decreases by 13% (95% CI 26% decrease to 6% increase) or 11% (95% CI 28% decrease to 6% increase), respectively (Additional file 1: Table S12). For one-level increase in PI/ECO similarity (reflecting less matching pairs), the RRR increases by 5% (95% CI 17% decrease to 18% increase).

## Discussion

### Principal findings

For the first time, the agreement of effect estimates from individual nutrition RCTs and cohort studies has been systematically evaluated, and determinants for the disagreement were explored. Overall, 64 highly matched RCT/cohort study pairs that mostly investigated the health impact of micronutrients (*n* = 40, 62.5%) have been included. The degree of PI/ECO similarity was deemed as convincing, 59/64 pairs were “more or less identical” or “similar but not identical”. Most of the included RCTs were classified as “low risk of bias” (26.6%) or with “some concerns” (65.6%), whereas cohort studies were often rated as “high risk of bias” (47.9%) or “very high risk of bias” (5.5%) - mostly due to inadequate control of important confounding factors.

We observed that on average RCTs and cohort studies had similar effect estimates. For binary outcomes, the pooled RRR was 1.00 (95% CI 0.91 to 1.10), and for continuous outcome pairs, the pooled DSMD was -0.26 (95% CI -0.87 to 0.35). Stratified analyses by dietary intervention/exposure, type of intake, outcome, similarity and risk of bias rating did not show differences between RCTs and cohort studies. However, effect estimates seem to be more imprecise and prediction intervals wider in "broadly similar" PI/ECO study pairs, suggesting that the difference in effect estimates could be considerably larger in either direction. Consequently, it cannot be concluded that in general there is no important difference. In meta-regression analyses exploring determinants of disagreements, the risk of bias judgements tend to have had more influence on the effect estimate in our sample than the “PI/ECO similarity” degree.

### Comparison with other studies

Influential publications on the credibility of observational studies in nutrition research provide prominent examples where RCTs either corroborate [251] or fail to confirm [252] the observed associations between dietary exposure and risk of non-communicable diseases in large cohort studies. However, these are only based on a selective presentation of study design comparisons to show either the large discordance or concordance. Therefore, a systematic evaluation such as ours, considering a comprehensive set of RCT/cohort study comparisons is highly needed. Our results support the assumption that findings of RCT and cohort studies - evaluating a highly similar research question - are often in agreement.

Meta-research studies evaluating bodies of evidence from nutrition RCTs and cohort studies included in the same evidence synthesis [11] or matching systematic reviews [10] found on average no differences or slight differences between effect estimates (RRR 1.04, 95% CI 0.99 to 1.10, and RRR 1.09, 95% CI 1.04 to 1.14 respectively). Differences in PI/ECO characteristics between the body of evidence of RCTs and cohort studies are assumed to be important drivers of disagreement and statistical heterogeneity.

Our findings are also in line with meta-epidemiological studies performed in the medical field: Bröckelmann 2022 [8] revealed a summary effect of 1.04 (95% CI 0.97 to 1.11) by considering bodies of evidence from RCTs and cohort studies for various medical research questions. The Cochrane Review by Toews 2024 [9] observed slight differences between effect estimates (RRR 1.09, 95% CI 1.04 to 1.13, *I*^2^ = 34%) when assessing the agreement between bodies of evidence from RCTs and cohort studies included in 14 methodological reviews. These authors acknowledged determinates of disagreement such as clinical and statistical heterogeneity between primary studies within meta-analyses, and the influence of bias, which is often not assessed (with established methods) in the included reviews.

The above-mentioned studies were all conducted at the systematic review level, where heterogeneity in studies within and between bodies of evidence is more challenging to address. Our shift to an individual study level complements this research by allowing a more detailed evaluation of determinants potentially affecting the agreement between RCTs and cohort studies. PI/ECO matching at the individual study level provides a higher degree of PI/ECO similarity, for example, through harmonisation of (dose-specific) effect estimates, and by selecting best-matching populations. Moreover, assessing risk of bias for each included RCT and cohort study further strengthens the validity of our approach. These considerations ensure better homogeneity compared to previous meta-research studies [8–11].

The authors of the RCT DUPLICATE initiative adopted a different approach for comparing RCTs and observational data in the field of medication treatment effects: Wang 2023 [253] used data of healthcare databases to replicate original RCTs, and drew similar conclusions, especially when closely emulating the PICO characteristics of the original trials. However, the authors assumed the RCT findings to be internally valid but did not provide a risk of bias assessment using validated tools, such as the RoB2 tool [17].

### Agreement and disagreement between RCT and cohort studies

In our sample of 64 RCT/cohort study pairs, investigating for instance the effects of the Mediterranean diet on cardiovascular disease, vitamin D supplementation on breast cancer, or low-fat diets on mortality, more than 80% (53/64 pairs) were in high agreement.

Nevertheless, in 11/64 pairs, effect estimates were not fully in agreement. In six of these eleven pairs, the effect estimates of RCTs and cohort studies were in the same direction, but 95% CI were wide. Notable differences in sample sizes (e.g. 4152 vs. 335,062 [233, 237]) or follow-up time (e.g. 0.23 vs. 12 years [45, 81]), may have caused imprecise RCT results.

In three study pairs on vitamin supplementation [46, 90, 91, 229, 230, 244], RCTs revealed large favourable/ detrimental effects, whereas cohort studies showed no effect or wide 95% CI. These cohort studies were rated as “high risk of bias” due to insufficient adjustment of our pre-defined confounding factors (e.g. alcohol intake and physical activity), whereas the RCTs were rated mainly as “some concerns”. Since both the RCTs and the cohort studies indicate substantial bias, exaggeration of intervention effects cannot be ruled out.

Other reasons for disagreement may yield from dissimilarities in PI/ECO characteristics. In the study pair “Jackson 2006/Prentice 2013” [41, 247], we observed differences in the categorisation of the intervention/exposure. The RCT examined the effects of daily doses of 500mg calcium carbonate with 200IU vitamin D_2_, whereas the cohort study classified exposure status by defining the group of users and non-users of calcium and vitamin D supplements. Participants with irregular or low intake may thus distort the findings, contributing to an underestimation of the effects or harms of the supplement use in the cohort study.

In two other discordant pairs, we observed notable differences in the study settings where RCTs and cohort studies were performed. For example, in the SELECT trial [44], participants from the US, Canada, and Puerto Rico were recruited, whereas the matched cohort [75] was based on a Danish population. The observed disagreement in effect estimates may thus arise from health disparities, rather than from the study design itself. In the SELECT trial, effect discrepancies of selenium on colorectal cancer risk may be linked to differences in race/ethnicity, healthcare systems, socioeconomic factors and lifestyle behaviour [254].

### Potential implications for research

Recently, the credibility of nutrition research has repeatedly been questioned, since several RCTs did not confirm the findings of large epidemiological studies [252]. However, our study shows clearly that most findings from individual RCTs were confirmed in best-matching cohort studies. The public health implications of our findings are therefore tremendous. For example, both RCTs and cohort studies showed a beneficial effect of the Mediterranean diet on type 2 diabetes [53, 78] and breast cancer [233, 237], healthy diet on type 2 diabetes [49, 239], or multivitamins/minerals on nuclear cataract [230, 244]. Moreover, both RCTs and cohort studies showed no effect of vitamin C on prostate cancer [36, 79] as well as vitamin D on breast cancer [26, 86]. Nevertheless, we encountered some study pairs with conflicting results. These are particularly noteworthy when one study design indicates a harmful effect while the other indicates no effect. We identified three examples of such a scenario: First, a harmful association of calcium supplementation on fracture risk [247]; second, vitamin C supplementation increases the risk of lung cancer [229]; third, multivitamin supplementation increases the risk of posterior subcapsular cataract [230]. However, these RCT findings need to be interpreted with caution, since the presence of statistical artefacts was not ruled out [229]. In general, these controversial findings cannot be solved at an individual study level. Definitive answers can only be obtained by considering the totality of evidence through high-quality systematic reviews [5].

To integrate successfully evidence from both study designs into future nutrition evidence syntheses, it is crucial to determine whether and under which circumstances similar findings can be expected. Our matching approach illustrates that study pairs with similar PI/ECO characteristics generally show concordant results. Additionally, we assume that study setting, sample size, duration of follow-up, as well as a higher risk of bias may serve as potential critical determinants elucidating conflicting results. Therefore, review authors should explore these determinants before deciding whether cohort studies and RCTs are “sufficiently similar” to be integrated in a systematic review.

Assessing the risk of bias sheds light on the credibility of individual studies, and the potential over- or underestimation of the true intervention effects. In our sample, inadequate adjustment for potential confounders was the main reason for “high risk of bias” in cohort studies. This highlights the need for systematic review authors to consider adjustment for potential confounders as a critical criterion for including cohort studies in a systematic review. Evidence from “low risk of bias” RCTs is considered as the most trustworthy source for estimating effect of intervention, whereas residual confounding can never be completely ruled out in cohort studies. Therefore our findings, showing notable disagreement in effect estimates between “low risk of bias” RCTs and cohort studies with “some concerns” are plausible. However, comparing “low risk of bias” RCTs with “high risk of bias” cohort studies did not confirm this observation. One explanation might be that the influence of the unconsidered important confounding factors is not substantial, or that the different biases observed operate in different directions, driving the effect estimates towards the null effect [18]. So in this context, more research is needed to evaluate the impact and direction of different methodological characteristics (pre-defined bias domains) on effect estimates in RCTs and cohort studies.

The GRADE working group recommends relying on the certainty of RCTs when determining whether or not to include cohort studies in systematic reviews [255]. This requires not only the risk of bias assessment, but also the other GRADE domains: indirectness, inconsistency, imprecision and publication bias [256]. Cohort studies can serve as a valuable source to complement the available body of evidence when the certainty of evidence of RCTs is anticipated to be low or very low [256]. For instance, when cohort studies align more closely with the PI/ECO criteria of the research question of interest, they may mitigate problems of indirectness. Moreover, considering that imprecision is a common reason for downgrading the certainty of evidence in nutrition systematic reviews of RCTs [257], incorporating findings from cohort studies may be valuable due to large sample sizes and number of cases. However, it is important to note that the inclusion of cohort studies may increase statistical inconsistency [10, 257]. In general, more research is needed on the evaluation of the certainty of evidence in the context of comparing bodies of evidence from RCTs and cohort studies [258].

### Strengths and limitations

Our meta-epidemiological study has several strengths. We examined a large sample of 64 highly PI/ECO-matched study pairs, encompassing various dietary interventions/exposures and health-related outcomes. Our extensive data extraction allowed us to adequately select and match RCTs and cohort studies, and to perform a rigorous examination of differences in PI/ECO characteristics. We conducted various statistical analyses, including 20 dose–response analyses, to recalculate and/or convert effect estimates to improve the comparability between the RCT and its matching cohort study. Moreover, we performed various subgroup analyses, sensitivity analyses, and meta-regressions to explore determinants associated with disagreements. We adhered to a stringent methodology throughout the whole review process, with standardised extraction sheets, established risk of bias tools [17, 18], and two independent reviewers in screening, matching, data extraction, as well as similarity and the risk of bias assessment.

Several limitations need to be taken in to account: First, although the matching process of RCTs and cohort studies was standardised and conducted by two independent reviewers, it has not been validated so far. However, we rely on PI/ECO similarity criteria used in previous studies [10, 11]. Second, for some identified pairs, more than one cohort was a suitable match for a respective RCT, and we considered the geographical location, sex, and age as additional pre-defined characteristics for matching. Prioritising other characteristics, such as the year of publication, may have resulted in choosing another cohort study and thus may have altered the findings. Third, we cannot rule out some degree of overlap. Some primary studies have contributed to more than one study pair (although with different diet-disease associations), which may have influenced the precision of our results. However, when each RCT was included only once in the sensitivity analysis, we found highly similar findings as compared to the main analysis. Fourth, we did not evaluate the impact of domain-specific risk of bias ratings on the effect estimates, but used the overall risk of bias rating to classify the individual studies at hand. However, domain-specific risk of bias might be an important driver of disagreement between RCTs and cohort studies and needs to be addressed in future research. Finally, although the evaluation at the individual study level allowed for close PI/ECO matching and risk of bias assessment, it may have constrained generalisability compared to the systematic review level and does not permit GRADE assessment.

## Conclusions

In our large sample of nutrition RCTs and cohort studies, effect estimates were on average similar. The influence of the investigated determinants of agreement was not substantial, probably due to the high PI/ECO similarity degree between RCTs and cohort studies. Nevertheless, prediction intervals appear to be wider in less similar study pairs, or when comparing “low risk of bias” RCTs to cohort studies with “some concerns”. Our findings highlight the importance of careful consideration and evaluation of study design-specific PI/ECO characteristics and especially risk of bias to enhance the trustworthy utilisation of evidence from nutrition RCTs and cohort studies. Finally, the identified determinates provide useful insights for a potential integration of both study designs in evidence syntheses.

## Supplementary Information

Additional file 1: Appendix 1. Description of eligibility criteria. Appendix 2. Criteria for Rating Population (P), Intervention/Exposure (I/E), Comparator (C), and Outcome (O) similarities. Appendix 3. Additional guidance to assess the risk of bias in cohort studies. Appendix 4. Additional guidance to assess the risk of bias in the included randomised controlled trials. Appendix 5. Methods to harmonise the type of effect estimates in study design pairs. Table S1. Overview of transformations made to the original data extraction. Table S2. Reasons for exclusion. Table S3. Characteristics of included randomised controlled trials. Table S4. Characteristics of included cohort studies. Table S5. Description of study design pairs. Table S6. Population (P), Intervention/Exposure (I/E), Control (C), and Outcome (O) similarity. Table S7. Overview of adjustments made in multivariable analysis in the included cohort studies. Table S8. Univariable meta-regression for PI/ECO similarity across pairs with binary outcomes. Table S9. Multivariable meta-regression for PI/ECO similarity (by domain) across pairs with binary outcomes. Table S10. Univariable meta-regression for risk of bias rating with the RoB2 tool across pairs with binary outcomes. Table S11. Univariable meta-regression for risk of bias rating with the ROBINS-E tool across pairs with binary outcomes. Table S12. Multivariable meta-regression for PI/ECO similarity and risk of bias rating across pairs with binary outcomes. Table S13. Overlaps between study design pairs. Figure S1. Risk of bias in individual randomised controlled trials. Figure S2. Risk of bias in randomised controlled trials (summary plot). Figure S3. Risk of bias in individual cohort studies. Figure S4. Risk of bias in cohort studies (summary plot). Figure S5. Forest plot of the comparison between bodies of evidence from randomised controlled trials versus those from cohort studies for continuous outcomes using difference of standardised mean difference. Figure S6. Forest plot of the comparison between study design pairs with binary outcomes / subgroup analysis by dietary intervention/exposure. Figure S7. Forest plot of the comparison between study design pairs with binary outcomes / subgroup analysis by type of intake. Figure S8. Forest plot of the comparison between study design pairs with binary outcomes / subgroup analysis by outcome. Figure S9. Forest plot of the comparison between study design pairs with binary outcomes / subgroup analysis by PI/ECO similarity. Figure S10. Forest plot of the comparison between study design pairs with binary outcomes / subgroup analysis by risk of bias rating. Figure S11. Forest plot of the comparison between study design pairs with binary outcomes / sensitivity analysis excluding pairs with high risk of bias rating. Figure S12. Forest plot of the comparison between study design pairs with binary outcomes / sensitivity analysis including each RCT only once for each outcome. Figure S13. Forest plot of the comparison between study design pairs with binary outcomes / sensitivity analysis including only RCT with largest sample size.

## Data Availability

Data were extracted from published studies (systematic review, randomised controlled trials and prospective cohort studies). All data generated or analysed during this study are included in this published article and its additional files. Data and codes for statistical analysis can be found under the following link: https://osf.io/j4d9n/.
