# Supplementary material for: Evaluating agreement between individual nutrition randomised controlled trials and cohort studies - a meta-epidemiological study
Source: BMC Med. 2025 Jan 21;23:36. doi: 10.1186/s12916-025-03860-2 (PMC11752614; doi:10.1186/s12916-025-03860-2)
Supplement: Supplementary file 1 — Additional file 1: Appendix 1. Description of eligibility criteria. Appendix 2. Criteria for Rating Population (P), Intervention/Exposure (I/E), Comparator (C), and Outcome (O) similarities. Appendix 3. Additional guidance to assess the risk of bias in cohort studies. Appendix 4. Additional guidance to assess the risk of bias in the included randomised controlled trials. Appendix 5. Methods to harmonise the type of effect estimates in study design pairs. Table S1. Overview of transformations made to the original data extraction. Table S2. Reasons for exclusion. Table S3. Characteristics of included randomised controlled trials. Table S4. Characteristics of included cohort studies. Table S5. Description of study design pairs. Table S6. Population (P), Intervention/Exposure (I/E), Control (C), and Outcome (O) similarity. Table S7. Overview of adjustments made in multivariable analysis in the included cohort studies. Table S8. Univariable meta-regression for PI/ECO similarity across pairs with binary outcomes. Table S9. Multivariable meta-regression for PI/ECO similarity (by domain) across pairs with binary outcomes. Table S10. Univariable meta-regression for risk of bias rating with the RoB2 tool across pairs with binary outcomes. Table S11. Univariable meta-regression for risk of bias rating with the ROBINS-E tool across pairs with binary outcomes. Table S12. Multivariable meta-regression for PI/ECO similarity and risk of bias rating across pairs with binary outcomes. Table S13. Overlaps between study design pairs. Figure S1. Risk of bias in individual randomised controlled trials. Figure S2. Risk of bias in randomised controlled trials (summary plot). Figure S3. Risk of bias in individual cohort studies. Figure S4. Risk of bias in cohort studies (summary plot). Figure S5. Forest plot of the comparison between bodies of evidence from randomised controlled trials versus those from cohort studies for continuous outcomes using difference of standardised mean difference. [file 12916_2025_3860_MOESM1_ESM.pdf]

# Additional File

**Evaluating agreement between individual nutrition randomised controlled trials and cohort studies – a meta-epidemiological study**

Julia Stadelmaier[1], Gina Bantle[1], Lea Gorenflo[1,2], Eva Kiesswetter[1], Adriani Nikolakopoulou[3,4], Lukas Schwingshackl[1]

[1] Institute for Evidence in Medicine, Medical Center - University of Freiburg, Faculty of Medicine, University of Freiburg, Freiburg, Germany.

[2] Cochrane Germany, Cochrane Germany Foundation, Freiburg, Germany.

[3] Institute of Medical Biometry and Statistics, Faculty of Medicine and Medical Center, University of Freiburg, Freiburg, Germany.

[4] Laboratory of Hygiene, Social and Preventive Medicine and Medical Statistics, School of Medicine, Aristotle University of Thessaloniki, Thessaloniki, Greece.

Corresponding author:

Julia Stadelmaier, MSc

Breisacher Straße 86, 79110 Freiburg, Germany

M: julia.stadelmaier@uniklinik-freiburg.de

P: +49 (0)761 270 85330

**Table of contents**

Appendix 1 Description of eligibility criteria ....................................................................................4

Appendix 2 Criteria for Rating Population (P), Intervention/Exposure (I/E), Comparator (C), and Outcome (O) similarities ..............................................................................................................5

Appendix 3 Additional guidance to assess the risk of bias in cohort studies ....................................6

Appendix 4 Additional guidance to assess the risk of bias in randomised controlled trials................. 11

Appendix 5 Methods to harmonise the type of effect estimates in study design pairs.......................... 14

Table S1 Overview of transformations made to the original data extraction ....................................... 15

Table S2 Reasons for exclusion ........................................................................................................ 20

Table S3 Characteristics of included randomised controlled trials ..................................................... 25

Table S4 Characteristics of included cohort studies............................................................................ 36

Table S5 Description of study design pairs.......................................................................................... 47

Table S6 Population (P), Intervention/Exposure (I/E), Control (C), and Outcome (O) similarity........ 52

Table S7 Overview of adjustments made in multivariable analysis in the included cohort studies...... 55

Table S8 Univariable meta-regression for PI/ECO similarity across pairs with binary outcomes........ 61

Table S9 Multivariable meta-regression for PI/ECO similarity (by domain) across pairs with binary outcomes............................................................................................................................................ 61

Table S10 Univariable meta-regression for risk of bias rating with the RoB 2 tool across pairs with binary outcomes ................................................................................................................................. 61

Table S11 Univariable meta-regression for risk of bias rating with the ROBINS-E tool across pairs with binary outcomes ........................................................................................................................ 61

Table S12 Multivariable meta-regression for PI/ECO similarity and risk of bias rating across pairs with binary outcomes ........................................................................................................................ 61

Table S13 Overlaps between study design pairs ................................................................................. 62

Figure S1 Risk of bias in individual randomised controlled trials ...................................................... 63

Figure S2 Risk of bias in randomised controlled trials (summary plot)............................................... 65

Figure S3 Risk of bias in individual cohort studies.............................................................................. 65

Figure S4 Risk of bias in cohort studies (summary plot) .................................................................... 67

Figure S5 Forest plot of the comparison between bodies of evidence from randomised controlled trials versus those from cohort studies for continuous outcomes using difference of standardised mean difference............................................................................................................................................ 68

Figure S6 Forest plot of the comparison between study design pairs with binary outcomes / subgroup analysis by dietary intervention/exposure ............................................................................................ 69

Figure S7 Forest plot of the comparison between study design pairs with binary outcomes / subgroup analysis by type of intake ...................................................................................................................... 70

Figure S8 Forest plot of the comparison between study design pairs with binary outcomes / subgroup analysis by outcome ............................................................................................................................ 71

Figure S9 Forest plot of the comparison between study design pairs with binary outcomes / subgroup analysis by PI/ECO similarity ........ 72
Figure S10 Forest plot of the comparison between study design pairs with binary outcomes / subgroup analysis by risk of bias rating ........ 73
Figure S11 Forest plot of the comparison between study design pairs with binary outcomes / sensitivity analysis excluding pairs with high risk of bias rating ........ 74
Figure S12 Forest plot of the comparison between study design pairs with binary outcomes / sensitivity analysis including each RCT only once for each outcome ........ 74
Figure S13 Forest plot of the comparison between study design pairs with binary outcomes / sensitivity analysis including only RCTs with largest sample size ........ 75

**Appendix 1** Description of eligibility criteria according to Schwingshackl 2021 and Stadelmaier 2024

| **Population** | **General population** |
|---|---|
| **Intervention/ Exposure**<br><br>(dietary intake or supplementation) | - **Dietary pattern:** e.g. Mediterranean diet, vegetarian diet, carbohydrate-restricted diet. OR<br>- **Food groups:** food groups (macro-level), and foods (micro-level) are considered: e.g. grains, vegetables, fruit, milk and dairy products, meat, fish, eggs, nuts, chocolate, oil. OR<br>- **Macronutrients:** *carbohydrates*, e.g. starch, fructose, glucose, sucrose; *fat*, e.g. omega–3 fatty acids (EPA, DHA, α-linolenic acid); omega–6 fatty acids (linoleic acid), monounsaturated fat; *proteins*, e.g. amino acids. OR<br>- **Micronutrients:** *vitamins*, e.g. β-carotene, vitamins A, E, C (ascorbic acid), and D (cholecalciferol, ergocalciferol); B vitamins (thiamine, riboflavin, niacin, pyridoxine, cobalamin, folic acid); *minerals*, e.g. calcium, magnesium, selenium, sodium, potassium, iron, zinc, copper, iodine. OR<br>- **Other:** fibre (psyllium, inulin, cellulose); probiotics; prebiotics; synbiotics. |
| **Control/ Comparison** | - **Low/ no intake or supplementation** of the above mentioned interventions/ exposure. OR<br>- **Placebo.** OR<br>- **Usual care.** |
| **Outcome** | - **Patient-relevant outcomes:** e.g. mortality, cancer, type 2 diabetes, dementia, age-related macular degeneration, *coronary heart disease*, e.g. myocardial infarction, ischemic heart disease, and acute coronary syndrome; *stroke*, e.g. ischemic or haemorrhagic.<br>- **Intermediate disease markers**: e.g. systolic and diastolic blood pressure, fasting glucose, LDL-cholesterol; body weight. |
| **Study designs** | - **Randomised controlled trials**: parallel, crossover, factorial, cluster design.<br>- **Cohort studies:** nested case-control, case-cohort studies, long-term prospective cohort studies. Retrospective and cross-sectional studies are excluded. |

DHA: docosahexaenoic acid; EPA: eicosapentaenoic acid; LDL: low-density lipoprotein

**Appendix 2** Criteria for Rating Population (P), Intervention/Exposure (I/E), Comparator (C), and Outcome (O) similarities (adapted from Schwingshackl 2021)

| Rating | Population | Intervention/ Exposure and Comparison | Outcome |
|---|---|---|---|
| **1 = more or less identical** | **Both studies include primary or secondary prevention.**<br>Example:<br>- both studies with healthy population, general population<br>- both studies with diseased population (e.g. people with cardiovascular disease)<br>**Both studies include same type of population regarding basic characteristics (e.g. age, gender)**<br>Example:<br>- both studies with adults<br>- both studies with postmenopausal women | **The studies use very similar intervention/ exposure.**<br>Example:<br>- folate supplementation in RCT and cohort study<br>- apple intake in RCT and cohort study<br>**Both studies consider the same dose of intake or supplements**<br>Example:<br>- 400IU/day of Vitamin D in RCT and cohort study | **The studies examine the same outcome.**<br>Example:<br>- all-cause mortality in RCTs and cohort studies |
| **2 = similar but not identical** | **Both studies include primary and secondary prevention *or* mixed population vs. primary or secondary prevention *or* high-risk population vs. primary or secondary prevention.**<br>Example:<br>- mixed population (healthy people and partly people with cardiovascular disease) in RCT and general healthy population in cohort study<br>- population at high-risk in RCT vs. general healthy population in cohort study | **The studies use similar intervention/ exposure:**<br>**- different interventions/exposures of the same class**<br>**- similar interventions/exposures with different co-interventions**<br>**- similar time frame of intervention.**<br>Example:<br>- multi-micronutrient supplementation vs. multivitamin supplementation<br>- intake of product + drug (e.g. aspirin) vs. intake of product<br>**Both studies do not consider the same dose of intake or supplements**<br>Example:<br>- 400IU/day of vitamin D in RCT and use (vs. non-use) in cohort study | **The studies examine the same cluster of outcomes.**<br>Example:<br>- cardiovascular disease in RCT and coronary heart disease in cohort study<br>- cardiovascular mortality in RCT and cardiovascular incidence + mortality in cohort study |
| **3 = broadly similar** | **One study includes primary prevention and the other study (mostly) secondary prevention.**<br>Example:<br>- people with cancer in RCT vs. general healthy population in cohort study<br>- mixed population (most [≥66%] of the people with cardiovascular disease) in RCT and general population in cohort study | **The studies use broadly similar intervention/ exposure:**<br>**- broadly similar categories**<br>**- different time frame/ early treatment vs. any treatment.**<br>Example:<br>- Vitamin C intake vs. fruit and vegetable intake<br>- pre-conception vs. post-conception use of folic acid | **The studies examine similar outcomes.**<br>Example:<br>- colorectal adenoma in RCT vs. colorectal cancer in cohort study |

RCT: randomised controlled trial;

**Appendix 3** Additional guidance to assess the risk of bias in cohort studies

<table>
<tr><th>Domain</th><th>Explanation</th><th>Judgements</th></tr>
<tr><td>Risk of bias due to confounding</td><td><ul><li>Is there potential for confounding of the effect of exposure in this study?</li><li>Did the authors use a multivariable-adjusted analysis method that controlled at least for age, sex, education/socioeconomic status, smoking, alcohol consumption, physical activity, weight/body mass index, and total energy intake (for exposures of dietary intake)?</li><li>Were confounding factors that were controlled for measured validly and reliably by the variables available in this study?</li><li>Did the authors avoid adjusting for post-exposure variables?</li></ul>Notes: Confounding is expected in all observational studies, thus no study was assigned low risk of bias. Time-varying confounding was expected to be unlikely and is not expected to cause risk of bias in the present study.</td><td><u>Low risk of bias:</u><br>No bias expected due to confounding, including time-varying confounding.<br><br><u>Some concerns:</u><br>Confounding is expected for age, sex, education/socioeconomic status, smoking, alcohol consumption, physical activity, weight/body mass index, (and total energy intake), and the authors performed a multivariable-adjusted analysis to control for these confounding factors. The variables adjusted for are valid and reliable measures of the confounding factors.<br>or Education/socioeconomic status (SES) is not included as confounding factor in the multivariable-adjusted analysis, but SES is not expected to vary substantially within the cohort (e.g. NHS, HPFS).<br>or The authors statistically investigated whether the confounding factors have an effect on the risk estimate and excluded the confounder from the multivariable model if there was no effect on the overall effect estimate.<br><br><u>High risk of bias:</u><br>At least one known important confounding factor was not measured or appropriately controlled for.<br>or The authors adjusted for post-exposure variables that are affected by exposure (e.g. sodium intake and risk of stroke [adjustment for blood pressure during follow-up = intermediate biological variable on the causal pathway] → over adjustment).<br><br><u>Very high risk of bias:</u><br>No adjustment was made for any covariate.<br>or<br>The authors controlled for post-exposure variables, and the use of negative controls, or other considerations, suggest serious uncontrolled confounding.</td></tr>
<tr><td colspan="3">Triage: The results of the first domain (risk of bias due to confounding) determined whether a full assessment is warranted. If the assessment of the first domain resulted in a (very) high risk of bias, no further domain will be considered for evaluation, as the overall judgement will already be at (very) high risk of bias.</td></tr>
</table>

| | | |
|---|---|---|
| **Risk of bias arising from measurement of exposure assessment** | • Does the measured exposure well-characterise the exposure metric specified to be of interest in this study?<br>• Was the exposure likely to be measured with error, or misclassified?<br>*Notes:* Differential misclassification is not expected to occur in prospective cohort studies, since diet is reported before the occurrence of the outcome (Freedman 2011).<br>Some type of non-differential misclassification cannot be excluded (any dietary assessment method involves measurement error), thus no study was assigned low risk of bias. | Low risk of bias:<br>Exposure status is well characterised by the measurement and no measurement error is expected in its assessment. *and* Exposure was measured at multiple times, and exposure is stable or change only slightly over time. *or* Exposure was measured with a single measurement, but exposure can be assumed to be stable over time.<br><br>Some concerns:<br>Exposure status is well characterised by the measurement, and exposure was measured using an established or validated tool (e.g. a validated food frequency questionnaire, multiple 24h recalls) *and* Exposure was measured at multiple times, and exposure is stable or change only slightly over time *or* Exposure was measured with a single measurement, but exposure can be assumed to be stable over time.<br><br>High risk of bias:<br>Exposure status is not well characterised by the measurement (e.g. assumed from an indirect measurement or important sources of dietary intake are not considered).<br>*and/or* Exposure was measured using a not validated tool.<br>*and/or* Exposure was measured with a single measurement, which is unlikely to characterise exposure during the period (e.g. single 24h recall) or exposure cannot be assumed to be stable over time.<br><br>Very high risk of bias:<br>Differential measurement error is expected (measurement error depends on the outcome). |
| **Risk of bias in selection of participants into the study** | • Was selection of participants into the study (or into the analysis) based on participant characteristics observed after the start of the exposure window being studied?<br>• Do start of follow-up and start of exposure coincide for most participants?<br>• Were methods used that are likely to correct for the presence of selection biases?<br><br>Notes: In observational studies, it is unlikely that post-exposure variables influenced selection of participants into the study. Exclusion of participants may be mostly based on missing data, which will be considered in the domain referring to missings (see below). | Low risk of bias:<br>All participants who would have been eligible for the target study were included in the study. *and* Start of exposure and follow-up coincide.<br><br>Some concerns:<br>Selection into the study may have been related to exposure and outcome; *and* the authors used appropriate methods to correct for the selection bias. *and/or* Start of exposure and follow-up do not coincide, but the effect of exposure is constant over time.<br><br>High risk of bias:<br>Selection into the study was related to exposure and outcome. *and/or* Start and exposure and follow-up do not coincide and the effect of exposure is not constant over time.<br>*and* This could not be corrected for in the analyses. |

| | | |
|---|---|---|
| | | Very high risk of bias:<br>Selection into the study was related to exposure and outcome. *and/or* Start and exposure and follow-up do not coincide and the effect of exposure is not constant over time.<br>*and* Sensitivity analysis is available, and demonstrates that there is evidence of substantial impact. |
| **Risk of bias due to post-exposure interventions** | • Were there post-exposure interventions that were influenced by prior exposure during the follow-up period? | Low risk of bias:<br>There were (probably) no interventions administered to alleviate the effect of exposures.<br><br>Some concerns:<br>Post-exposure interventions were identified and the analysis corrected for the effect of these interventions.<br><br>High risk of bias:<br>Post-exposure interventions were identified and the analysis did not correct for the effect of these interventions |
| **Bias due to missing data** | • Were there missing outcome data?<br>• Were participants excluded due to missing data on exposure status?<br>• Were participants excluded due to missing data on other variables needed for analysis?<br>• Did the authors perform a complete case analysis?<br>• Was an appropriate method used to correct for bias due to missing data (e.g. appropriate imputation)?<br><br>*Notes:* Missing data on exposure variables and other variables are expected to be missing at random and not related to exposure or outcome that have been assessed during follow-up. | Low risk of bias:<br>Little loss-to-follow-up (<20%) and data on exposure and other variables were reasonably complete (<10% missing data) and was unlikely to introduce bias.<br>*or* The analysis addressed missing data and is likely to have removed any risk of bias.<br><br>Some concerns:<br>There is a proportion of missing data in the original cohort or a high proportion of loss-to-follow-up. *and* The analysis is unlikely to have removed the risk of bias arising from the missing data (e.g. using logistic regression).<br><br>High risk of bias:<br>High proportions (>50%) of missing data. *and* The analysis is unlikely to have removed the risk of bias arising from the missing data.<br>*or* The nature of the missing data means that the risk of bias cannot be removed through appropriate analysis.<br><br>Very high risk of bias:<br>High proportions (>50%) of missing data; and missing data were addressed inappropriately in the analysis. |

| | | |
|---|---|---|
| **Risk of bias due to measurement of the outcome** | • Were the methods of outcome assessment comparable across exposure groups?<br>• Could the outcome measure have been influenced by knowledge of the exposure status?<br>• Were any systematic error in measurement of the outcome related to exposure status?<br><br>*Notes:* In observational studies, it is not expected that outcome assessors were aware of exposure status of the participants. | Low risk of bias:<br>The methods of outcome assessment were comparable across exposure groups. *and* The outcome measure was unlikely to be influenced by knowledge of the exposure status of study participants *and* Any error in measuring the outcome is unrelated to exposure status (i.e. objective measures or self-reported outcomes that are (mostly, ≥90%) confirmed by a second source (e.g. medical records, record linkage and death certificates)).<br><br>Some concerns:<br>The methods of outcome assessment were comparable across exposure groups. *and* Any error in measuring the outcome may be minimally related to exposure status.<br>*or* Outcome measure are not reliable measured (i.e. confirmed records are available for <90% of all participants and the authors did not perform an additional analysis separating confirmed and probable cases).<br><br>High risk of bias:<br>The methods of outcome assessment were not comparable across exposure groups. *or* The outcome measure was subjective (i.e. self-report of cardiovascular disease, type 2 diabetes, etc. by study participants or next of kin, without confirmation by a second source). *and* Error in measuring the outcome was related to exposure status. |
| **Risk of bias due to selection of the reported result** | • Was the result reported in accordance with an available, pre-determined analysis plan?<br>• Is the reported effect estimate likely to be selected from multiple exposure measurements?<br>• Is the reported effect estimate likely to be selected from multiple analyses of exposure-outcome relationship?<br>• Is the reported effect estimate likely to be selected from different subgroups?<br><br>*Notes:* In observational studies, it is unusual to publish an a priori analysis plan or protocol. Therefore, if the authors present a clear description of the conducted analyses (i.e. methods section), and it appears to be consistent with the reported results; and the reported results correspond to all intended outcomes, analyses and sub cohorts (e.g. postmenopausal women), low risk of bias can be adequate. | Low risk of bias:<br>The results are reported according to an a-priori analysis plan or protocol. There is a clear description of all analysis and the analyses are consistent, and all reported results correspond to all intended outcomes, analyses and sub-cohorts.<br><br>Some concerns:<br>The results are reported according to an a-priori analysis plan or protocol, and there is indication of selection of the reported analysis among multiple analyses; *or* there is indication of selection of the cohort or subgroups for analysis and reporting on basis of the results (e.g. estimates not shown for all analyses).<br>*or* There is no a-priori analysis plan or protocol and there appear to be no issues with the exposure, multiple analyses (e.g., effect estimates were similar when different multiple analyses were used), or the selection or definition of subgroups, but there are inconsistencies/or no information between intended and reported analyses. |

| | | |
|---|---|---|
| | However, if there are any inconsistencies/or no information between intended analyses and reported results, e.g. inconsistencies between the adjustments described in the methods section and the adjustments for the corresponding reported estimates in the results section; there is reason for some concerns in this domain.<br><br>Multiple outcome measurements for the definition of cardiovascular disease, mortality, type 2 diabetes, etc. are not expected. | High risk of bias:<br>There is a high risk of selective reporting from multiple exposure measurements, *or* outcomes measurements, *or* multiple analyses of data.<br>*or* The cohort or subgroup is selected from a larger study for analysis and appears to be reported based on the results. (up to 2).<br><br>Very high risk of bias:<br>There is a high risk of selective reporting from multiple exposure measurements, *or* outcomes measurements, *or* multiple analyses of data *or* the cohort or subgroup is selected from a larger study for analysis and appears to be reported based on the results. (more than 2) |
| **Overall judgement** | Low risk of bias | The study is judged to be at low risk of bias for all domains. |
| | Some concerns | The study is judged to be at low risk of bias or some concerns for all domains. |
| | High risk of bias | The study is judged to be at high risk of bias in at least one domain, but no domains are at very high risk of bias. |
| | Very high risk of bias | The study is judged to be at very high risk of bias in at least one domain. |

**Appendix 4** Additional guidance to assess the risk of bias in randomised controlled trials

| **Domain 1: Risk of bias arising from randomisation process** | |
|---|---|
| **1.1** Was the allocation sequence random? | No information about randomisation method → NI |
| **1.2** Was the allocation sequence concealed until participants were enrolled and assigned to interventions? | Check if allocation was concealed (e.g. by using envelopes or a central or external enrolment service).<br><br>If appropriate allocation concealment can be assumed → PY/Y<br>If no information about allocation concealment is reported → NI |
| **1.3** Did baseline differences between intervention groups suggest a problem with the randomisation process? | Check group sizes.<br>Look for imbalances for key variables such as age, gender, health status, baseline values of outcomes.<br><br>Baseline tables:<br>If p-values are given, check for significant differences in baseline characteristics between intervention groups.<br>If p-values are not given, check (by eye) for large/obvious baseline imbalances between intervention groups.<br><br>Text:<br>If small or no significant imbalances are reported → PN/N |

| **Domain 2: Risk of bias due to deviations from the intended interventions** | |
|---|---|
| **2.1** Were participants aware of their assigned intervention during the trial? | In studies with dietary interventions other than supplementation of vitamins/minerals, blinding is likely not possible due to the nature of the included interventions → Y/PY |
| **2.2** Were carers and people delivering the interventions aware of participants' assigned intervention during the trial? | In studies with dietary interventions other than supplementation of vitamins/minerals, blinding is likely not possible due to the nature of the included interventions → Y/PY |
| **2.3.** If Y/PY/NI to 2.1 or 2.2: Were there deviations from the intended intervention that arose because of the trial context? | Check if<br>(a) additional interventions that were introduced were not consistent with trial protocol<br>(b) failure to implement the protocol interventions as intended was evident<br><br>If no reasons or details of deviations from the planned interventions are reported, it is likely that no deviations occurred → PN/N<br><br>If reported deviations are expected to arise in usual care, e.g. disliked diet, missed visits, lost interest, difficulty following diet → PN/N |
| **2.4** If Y/PY to 2.3:<br>Were these deviations likely to have affected the outcome? | Judge whether the above mentioned aspects/deviations had an impact on the outcome. |
| **2.5** If Y/PY/NI to 2.4: Were these deviations from intended intervention balanced between groups? | See guidance |

| | |
|---|---|
| **2.6** Was an appropriate analysis used to estimate the effect of assignment to intervention? | If ITT or modified ITT was used → Y/PY<br><br>If ITT or modified ITT can be assumed (i.e. number randomised per group = number analysed per group) → Y/PY<br><br>If no details of the analysis are reported (i.e. number randomised per group ≠ number analysed per group, with no information about excluded participants) → NI<br><br>If per protocol analysis was used (with investigators actively excluding available data, e.g. due to reasons related to compliance) → PN/N |
| **2.7** If N/PN/NI to 2.6: Was there potential for a substantial impact (on the result) of the failure to analyse participants in the group to which they were randomised? | Cut-off: >5% missing per group (excluded or analysed in wrong group) |

| **Domain 3: Risk of bias due to missing outcome data** | |
|---|---|
| **3.1** Were data for this outcome available for all, or nearly all, participants randomised? | Note that imputed data should be regarded as missing data, and not considered as 'outcome data' in the context of this question.<br><br>Cut-off: ≥20% missing data → N/PN<br><br>Low RoB: <20% + valid reasons<br>Some concerns: <20% without valid reasons<br>However, if valid imputation techniques mentioned → low RoB |
| **3.2** If N/PN/NI to 3.1: Is there evidence that the result was not biased by missing outcome data?<br><br>**3.3** If N/PN to 3.2: Could missingness in the outcome depend on its true value?<br><br>**3.4** If Y/PY/NI to 3.3: Is it likely that missingness in the outcome depended on its true value? | Check if<br>(a) (multiple) imputation was used<br>(b) Sensitivity analysis were conducted<br>(c) Reasons were given<br><br>High risk: > 20%<br>However, if:<br>- valid imputation techniques mentioned → low RoB<br>- no imputation techniques are used, but valid reasons are mentioned for both groups and are (nearly) equally distributed across groups, we will not assume high RoB |

| Domain 4: Risk of bias in measurement of the outcome | |
|---|---|
| **4.1** Was the method of measuring the outcome inappropriate? | - For (binary) disease outcomes, check if self-reported outcomes were validated by a second source (register, medical records).<br>- For anthropometric measures, check if a standardised protocol was used.<br>- For blood glucose, verify that measurement was not done with a portable tool by participants.<br>- Blood pressure, check if standardised protocol was used and measurement was performed by the researcher. |
| **4.2** Could measurement or ascertainment of the outcome have differed between intervention groups? | Check if outcome measurement differed between groups.<br>If Y/PY → high RoB |
| **4.3** If N/PN/NI to 4.1 and 4.2: Were outcome assessors aware of the intervention received by study participants? | If N/PN → low RoB |
| **4.4** If Y/PY/NI to 4.3: Could assessment of the outcome have been influenced by knowledge of intervention received? | See guidance |
| **4.5** If Y/PY/NI to 4.4: Is it likely that assessment of the outcome was influenced by knowledge of intervention received? | See guidance |

| Domain 5: Risk of bias in selection of the reported result | |
|---|---|
| **5.1** Were the data that produced this result analysed in accordance with a pre-specified analysis plan that was finalised before unblinded outcome data were available for analysis?<br><br>Is the numerical result being assessed likely to have been selected, on the basis of the results, from...<br><br>**5.2.** ... multiple eligible outcome measurements (e.g. scales, definitions, time points) within the outcome domain?<br><br>**5.3** ... multiple eligible analyses of the data? | Check if<br>(a) protocol or trial registry entry is available<br>(b) information on the pre-specified analysis is given<br>(c) changes to the pre-specific analysis plan were made (check also history of changes of the register entry)<br>(d) deviations were reported in the manuscript.<br><br>If no study protocol/registration is available and no deviations are reported in the manuscript. → some concerns<br>If registry entry is available but no information about the analysis plan exists. → some concerns<br><br>If study protocol/registration is present and there is no evidence for differences between protocol and report. → low RoB<br>If study protocol/registration is present and differences between protocol and report were clearly described and justified in the text.<br>→ low RoB<br><br>Cave: Only consider outcome pre-specification information that is dated before the end of trial. |

ITT: intention-to-treat analysis, NI: no information, PN/N: partial no/no, PY/Y: partial yes/yes, RoB: risk of bias

**Appendix 5** Methods to harmonise the type of effect estimates in study design pairs

To ensure consistent usage of effect estimates, we used appropriate formula for converting different type of effect measures. Binary outcomes were expressed as risk ratios [RR]. We transformed odds ratios [OR] into RR using an assumed control risk (ACR; $RR = \frac{OR}{1-ACR \times (1-OR)}$ ). When effect estimates of randomised controlled trials were expressed in hazard ratios, we computed de novo a risk ratio based on the number of participants randomised and number of cases with the outcome of interest, in each arm. In cohort studies, we took the adjusted OR and HR as approximation of the RR. For continuous outcomes, we computed mean differences for measurements that were on the same scale, and standardised mean differences for comparisons between differing outcome scales.

Where necessary, we recalculated and/or converted effect estimates to improve the comparability between the RCT and its matching cohort study: If no effect estimate was reported in individual studies, we used the number of cases and participants per group to derive risk ratios. Moreover, when a publication was based on several cohort studies (e.g. Nurses' Health Study [NHS] and the Health Professional Follow-up Study [HPFS]) or presented only sex-specific effect estimates, we pooled these effect estimates using a fixed-effect model. For data harmonisation, we standardised as recommended the direction of the effect for all studies, to ensure that binary effect estimates <1 are expressing a beneficial effect. If intake/supplementation dose differed between the RCT and its matching cohort study, we attempted to convert effect estimates to a standardised dose, using the RCT dose as the reference. We used the generalized least squares method described by Longnecker and Greenland 1992 to estimate the RR for the dose used in the RCT.

**Table S1** Overview of transformations made to the original data extraction

| Reference | Intervention/ Exposure | Outcome | Original effect estimate (95% CI) | What we used: effect estimate (95% CI) | Rationale |
|---|---|---|---|---|---|
| **Randomised controlled trials** | | | | | |
| Baron 2015 | Vitamin D | Nephrolithiasis | NR | RR 0.68 (0.38 to 1.21) | ES generated with data presented in Table 3 |
| Barr 2000 | Dairy | Systolic blood pressure | NR | MD 1.00 (-1.26 to 3.26)<br>SMD 0.12 (-0.16 to 0.40) | ES generated with data presented in Table 6, MD converted to SMD |
| Brough 2010 | Multivitamins | Preterm birth | NR | RR 1.09 (0.43 to 2.77) | ES generated with data presented in Table 4 |
| Brunner 2011 | Vitamin D | Breast cancer | HR 0.96 (0.85 to 1.09) | RR 0.96 (0.85 to 1.09) | HR recalculated to RR with data presented in Table 2 |
| Burr 1989 | PUFA | MACCE | NR | RR 0.99 (0.87 to 1.12) | ES generated with data taken from Abdelhamid 2018b, with data presented in Analysis 1.39 |
| Chai 2012 | Apples | Body weight (kg) | NR | MD -2.00 (-5.79 to 1.79)<br>SMD 0.21 (-0.60 to 0.19) | ES generated with data presented in Table 2, MD converted to SMD |
| Charles 2005 | Folic acid | Pre-eclampsia | 5mg: OR 0.65 (0.31 to 1.38)<br>200µg: OR 0.55 (0.25 to 1.25) | RR 0.61 (0.34 to 1.08) | OR recalculated to RR, combine groups (pooled ES) |
| Christian 2003 | Folic acid | Low birthweight | NR | RR 0.96 (0.85 to 1.09) | ES generated with data presented in Table 5 |
| Christian 2003 | Folic acid | Birthweight | MD -0.02 (-0.071 to 0.032) | SMD 0.00 (-0.11 to 0.11) | MD converted to SMD with data presented in Table 4 |
| Czeizel 1994 | Folic acid | Neural tube defect | NR | RR 0.07 (0.00 to 1.32) | ES generated with data presented in Table 6 |
| Czeizel 1998 | Folic acid | Congenital heart defects | OR 0.42 (0.19 to 0.98) | RR 0.48 (0.23 to 1.03) | OR recalculated to RR with data presented in Table 4 |
| de Lorgeril 1998 | Mediterranean diet | Cancer mortality | NR | RR 0.75 (0.17 to 3.33) | ES generated with data presented in Table 2 |
| Esposito 2009 | Mediterranean diet | HDL-Cholesterol (mmol/l) | MD 0.07 (0.02 to 0.14) | SMD 1.19 (0.90 to 1.48) | MD converted to SMD with data presented in Table 2 |
| Esposito 2009 | Mediterranean diet | Systolic blood pressure | MD -1.5 (-4.5 to -1.2) | SMD -0.76 (-1.03 to -0.48) | MD converted to SMD with data presented in Table 2 |
| Esposito 2009 | Mediterranean diet | Triglycerides (mmol/l) | MD -0.21 (-0.36 to -0.02) | SMD -0.99 (-1.28 to -0.71) | MD converted to SMD with data presented in Table 2 |
| Estruch 2018 | Mediterranean diet | All-cause mortality | HR 0.98 (0.77 to 1.24) | RR 1.01 (0.81 to 1.25) | HR recalculated to RR with data presented in Table 3 |
| Estruch 2018 | Mediterranean diet | Cardiovascular disease | HR 0.70 (0.55 to 0.89) | RR 0.81 (0.64 to 1.02) | HR recalculated to RR with data presented in Table 3 |
| Estruch 2018 | Nuts | Coronary heart disease | NR | RR 0.79 (0.55 to 1.14) | ES taken from Afshin 2014: dose-response analysis (per 4 servings/week) |

| | | | | | |
|---|---|---|---|---|---|
| Estruch 2018 | Nuts | Stroke | NR | RR 0.62 (0.44 to 0.87) | ES taken from Afshin 2014: dose-response analysis (per 4 servings/week) |
| Gaziano 2009 | Vitamin C | Prostate cancer | HR 1.02 (0.90 to 1.15) | RR 1.01 (0.90 to 1.14) | HR recalculated to RR with data presented in Table 2 |
| Gaziano 2009 | Vitamin E | Prostate cancer | HR 0.97 (0.85 to 1.09) | RR 0.96 (0.85 to 1.08) | HR recalculated to RR with data presented in Table 2 |
| Heinonen 1998 | β-carotene | Prostate cancer | NR | RR 1.24 (0.96 to 1.59) | ES generated with data presented in Table 2 |
| Hollis 2011 | Vitamin D | Pre-eclampsia | NR | RR 0.50 (0.20 to 1.23) | ES generated with data taken from Hollis 2013, with data presented in Table 2 |
| Howard 2006 | Low-fat diet | All-cause mortality | NR | RR 0.98 (0.90 to 1.06) | ES generated with data presented in Figure 1 |
| Howard 2006 | Low-fat diet | Cardiovascular disease mortality | NR | RR 0.99 (0.81 to 1.20) | ES generated with data presented in Table 4 |
| Hsia 2007 | Calcium | Cardiovascular disease mortality | HR 1.01 (0.79 to 1.29) | RR 1.01 (0.79 to 1.29) | HR recalculated to RR with data presented in Table 2 |
| Jackson 2006 | Calcium | All fractures | HR 0.96 (0.91 to 1.02) | RR 0.97 (0.92 to 1.03) | HR recalculated to RR with data presented in Table 2 |
| Karp 2013 | Selenium | Oesophageal cancer | NR | RR 1.50 (0.06 to 36.86) | ES generated with data presented in Table 2 |
| Kirke 1992 | Multivitamins | Stillbirth | NR | RR 2.43 (0.12 to 50.05) | ES generated with data presented in Table 2 |
| Lippman 2009 | Selenium | Colorectal cancer | HR 1.05 (0.66 to 1.67) | RR 1.04 (0.73 to 1.48) | HR recalculated to RR with data presented in Table 4 |
| Lippman 2009 | Selenium | Prostate cancer | HR 1.04 (0.87 to 1.24) | RR 1.03 (0.90 to 1.18) | HR recalculated to RR with data presented in the results section |
| Maki 2010 | Whole grain | Body weight | NR | MD 0.10 (-0.06 to 0.10)<br>SMD -1.33 (-1.66 to -1.00) | ES generated with data presented in the results section (modified ITT-analysis), MD converted to SMD |
| Merchant 2005 | Folic acid | Gestational hypertension | HR 0.62 (0.40 to 0.94) | RR 0.63 (0.42 to 0.94) | HR recalculated to RR with data presented in Table 2 |
| Meyer 2005 | Multivitamins | Prostate cancer | HR 0.88 (0.60 to 1.29) | RR 0.90 (0.62 to 1.33) | HR recalculated to RR with data presented in Table III |
| Moses 2014 | Healthy diet | Small for gestational age | NR | RR 0.86 (0.48 to 1.54) | ES generated with data presented in the results section |
| Pan 1997 | Healthy diet | All-cause mortality | NR | RR 1.02 (0.21 to 4.98) | ES generated with data presented in the results section |
| Pan 1997 | Healthy diet | Type 2 Diabetes | NR | RR 0.65 (0.52 to 0.81) | ES generated with data presented in Table 2 |
| Reid 2007 | Low dietary sugar | Body weight change | NR | MD -1.37 (-2.35 to -0.39)<br>SMD -0.47 (-0.81 to -0.12) | insufficient study data provided; ES taken from Malik 2013 with author correspondence; converted to low vs. high |
| Riggs 1998 | Calcium | Nephrolithiasis | NR | RR 0.33 (0.01 to 7.96) | ES generated with data presented in the discussion section |
| Salas-Salvadó 2008 | Mediterranean diet | Metabolic syndrome | NR | RR 0.90 (0.82 to 1.00) | ES generated (prevalence) with data presented in the Table 1 and Table 5 |

| | | | | | |
|---|---|---|---|---|---|
| Salas-Salvado 2014 | Olive Oil | Type 2 Diabetes | HR 0.60 (0.42 to 0.84) | RR 0.89 (0.82 to 0.96) | ES taken from Afshin 2014: dose-response analysis (per 4 servings/week); ES recalculated from 45mg/day to 10g/day |
| Salas-Salvadó 2014 | Nuts | Type 2 Diabetes | HR 0.82 (0.61 to 1.10) | RR 0.86 (0.68 to 1.08) | ES taken from Afshin 2014: dose-response analysis (per 4 servings/week) |
| Sichieri 2009 | Low dietary sugar | Body mass index | MD 0.10 (-0.06 to 0.10) | SMD 0.08 (-0.05 to 0.21) | MD converted to SMD with data presented in Table 2 |
| TOHP II 1997 | Low-sodium | All-cause mortality | NR | RR 0.67 (0.36 to 1.25) | ES generated with data presented in WHO 2012, Figure 3.19 |
| Walsh 2012 | Healthy diet | Preterm birth | NR | RR 0.39 (0.10 to 1.46) | ES generated with data presented in results section (fetal outcomes) |
| Walsh 2012 | Healthy diet | Birth weight (in kg) | MD 0.0286 (-0.0456 to 0.1028) | SMD 0.06 (-0.09 to 0.20) | MD converted to SMD with data presented in Table 2 |
| Walsh 2012 | Mediterranean diet | Gestational diabetes | NR | RR 0.71 (0.35 to 1.45) | ES generated with data presented in Table 3 |
| Whelton 1998 | Low-sodium | Cardiovascular disease | NR | RR 0.78 (0.52 to 1.18) | ES generated with data presented in WHO 2012, Figure 3.3 |
| Zhang 2008 | Folic acid | Breast cancer | RR 0.83 (0.60 to 1.14) | RR 0.83 (0.61 to 1.14) | HR recalculated to RR with data presented in Table 2 |
| Zhang 2008 | Folic acid | Colorectal cancer | RR 0.81 (0.43 to 1.50) | RR 0.82 (0.44 to 1.52) | HR recalculated to RR with data presented in Table 2 |
| Zhang 2008 | Folic acid | Pancreatic cancer | HR 1.49 (0.42 to 5.30) | RR 1.50 (0.42 to 5.31) | HR recalculated to RR with data presented in Table 2 |
| **Cohort studies** | | | | | |
| Bao 2013 | Nuts | Coronary Heart Disease | Highest vs. lowest category:<br>NHS HR 0.72 (0.55 to 0.94)<br>HPFS HR 0.71 (0.61 to 0.83) | RR 0.81 (0.75 to 0.88) | ES taken from Afshin 2014: dose-response analysis (per 4 servings/week); NHS and HPFS pooled with fixed effects model |
| Bernstein 2012 | Nuts | Stroke | Highest vs. lowest category:<br>NHS HR 0.86 (0.75 to 0.98)<br>HPFS HR 0.92 (0.77 to 1.09) | RR 0.90 (0.79 to 1.02) | ES taken from Afshin 2014: NHS and HPFS pooled with fixed effects model |
| Bertoia 2015 | Apples | Body weight (in kg) | NHS MD -0.65 (-0.73 to -0.56)<br>NHSII MD -0.39 (-0.48 to -0.29) | MD -0.53 (-0.59 to -0.47) | NHS and NHSII pooled with fixed effects model; insufficient data to convert MD to SMD |
| Cohen 2008 | Low-sodium | All-cause mortality | HR 0.94 (0.88 to 1.01) | RR 1.06 (0.99 to 1.14) | ES converted to low vs. high; HR=RR |
| Cohen 2008 | Low-sodium | Cardiovascular disease | HR 0.88 (0.77 to 1.01) | RR 1.14 (0.99 to 1.30) | ES converted to low vs. high; HR=RR |
| Cui 2008 | Vitamin C | Breast Cancer | Highest vs. lowest category:<br>RR 1.16 (1.04 to 1.30) | RR 1.08 (1.02 to 1.14) | dose-response per 500mg/day |
| Curhan 1997 | Calcium | Nephrolithiasis | Highest vs. lowest category:<br>RR 1.21 (0.96 to 1.52) | RR 1.59 (0.95 to 2.64) | dose-response per 1,600mg/day |
| Czeizel 2004 | Folic acid | Congenital heart defects | HR 0.60 (0.38 to 0.96) | RR 0.60 (0.38 to 0.96) | crude ES, recalculated HR to RR with data presented in Table 4 |
| Dong 2008 | Selenium | Oesophageal cancer | Highest vs. lowest category:<br>HR 0.27 (0.03 to 2.21) | RR 0.02 (0.00 to 1.94) | dose-response per 200µg/day; HR=RR |

| | | | | | |
|---|---|---|---|---|---|
| Egnell 2017 | Vitamin C | Colorectal cancer | Highest vs. lowest category:<br>HR 0.45 (0.14 to 1.41) | RR 0.00 (0.00 to 11.67) | dose-response per 500mg/day; HR=RR |
| Ferraro 2017 | Vitamin D | Nephrolithiasis | Highest vs. lowest category:<br>NHS HR 1.03 (0.71 to 1.51)<br>NHSII HR 1.38 (1.03 to 1.85)<br>HPFS HR 1.23 (0.81 to 1.86) | RR 1.10 (0.99 to 1.22) | dose-response per 1,000IU/day; NHS, NHS II and HPFS pooled with fixed effect model; HR=RR |
| Gresham 2016 | Healthy diet | Small for gestational age | OR 0.49 (0.19 to 1.25) | RR 0.42 (0.11 to 1.09) | ES converted OR to RR with ACR=0.079; risk observed in the control group of the corresponding RCT (Moses 2014) |
| Hansen 2013 | Selenium | Colorectal cancer | Lowest vs. highest category:<br>HR 1.25 (1.05 to 1.48) | RR 0.52 (0.32 to 0.86) | ES converted to high vs. low; dose-response per 200µg/day; HR=RR |
| Haugen 2009 | Vitamin D | Pre-eclampsia | Highest vs. lowest category:<br>OR 0.83 (0.68 to 1.03) | RR 0.68 (0.47 to 0.99) | dose-response per 2,000IU/day; converted OR to RR with ACR=0.077; risk observed in the control group of the corresponding RCT (Hollis 2011) |
| Hillesund 2014 | Healthy diet | Preterm birth | OR 0.91 (0.80 to 1.03) | RR 0.91 (0.80 to 1.03) | recalculated OR to RR with ACR=0.021; risk observed in the control group of the corresponding RCT (Walsh 2012) |
| InterAct 2011 | Olive Oil | Type 2 Diabetes | HR 0.97 (0.95 to 1.01) | RR 0.88 (0.81 to 0.95) | ES taken from Schwingshackl 2015: dose-response analysis (per 10g/day); men and women pooled with fixed effects model |
| Kirsh 2006 | Vitamin C | Prostate cancer | Highest vs. lowest category:<br>RR 1.01 (0.87 to 1.17) | RR 1.01 (0.94 to 1.09) | dose-response per 500mg/day |
| Kirsh 2006 | β-carotene | Prostate cancer | Highest vs. lowest category:<br>RR 0.82 (0.65 to 1.04) | RR 0.55 (0.10 to 2.92) | dose-response per 20,000µg/day (20mg/day) |
| Lawson 2007 | Multivitamins | Prostate cancer | Highest vs. lowest category:<br>RR 1.06 (0.97 to 1.17) | RR 1.03 (0.99 to 1.07) | ES recalculated to 7 times/week vs. none with data presented in Table 2 |
| Liu 2003 | Whole grain | Body weight | NR | MD -0.29 (-0.35 to -0.23)<br>SMD -0.12 (-0.14 to -0.10) | ES generated with data provided in Tables 1 and 2, MD to SMD |
| Ludwig 2001 | Low dietary sugar | Body mass index | MD 0.24 (0.10 to 0.39) | NA | insufficient data to convert MD to SMD |
| Maruti 2009 | Folic acid | Breast cancer | Highest vs. lowest category:<br>RR 1.00 (0.84 to 1.19) | RR 0.91 (0.35 to 2.36) | dose-response per 2,500µg/day |
| Pan 2013 | Nuts | Type 2 Diabetes | Highest vs. lowest category:<br>NHS HR 1.00 (0.87 to 1.14)<br>NHSII HR 1.02 (0.85 to 1.23) | RR 0.88 (0.83 to 0.94) | ES taken from Afshin 2014: dose-response analysis (per 4 servings/week); NHS and NHS II pooled with fixed effects model |
| Peters 2008 | Selenium | Prostate cancer | Highest vs. lowest category:<br>HR 0.90 (0.62 to 1.30) | RR 0.83 (0.37 to 1.89) | dose-response per 200µg/day; HR=RR |
| Robien 2007 | Vitamin D | Breast cancer | Highest vs. lowest category:<br>RR 0.89 (0.74 to 1.08) | RR 0.96 (0.91 to 1.01) | dose-response per 400IU/day |
| Rodriguez 2004 | Vitamin E | Prostate cancer | Highest vs. lowest category:<br>RR 0.98 (0.89 to 1.08) | RR 1.00 (0.97 to 1.03) | dose-response per 200IU/day |
| Schulze 2004 | Low dietary sugar | Body weight change | NR | MD -1.55 (-2.00 to -1.10)<br>SMD -0.25 (-0.32 to -0.17) | ES generated; converted MD to SMD |
| Skinner 2004 | Folic acid | Pancreatic cancer | Highest vs. lowest category:<br>NHS RR 1.26 (0.76 to 2.08) | RR 5.86 (0.25 to 139.27) | dose-response per 2,500µg/day |
| Slatore 2008 | Folic acid | Lung cancer | Highest vs. lowest category:<br>HR 0.97 (0.79 to 1.23) | RR 0.92 (0.40 to 2.11) | dose-response per 2,000µg/day; HR=RR |

| | | | | | |
|---|---|---|---|---|---|
| Slatore 2008 | Vitamin C | Lung cancer | Highest vs. lowest category:<br>HR 0.97 (0.76 to 1.23) | RR 0.95 (0.74 to 1.21) | dose-response per 500mg/day; HR=RR |
| Timmermans 2011 | Folic acid | Gestational hypertension | OR 1.24 (0.91 to 1.69) | RR 1.21 (0.92 to 1.57) | OR converted to RR with ACR=0.115; risk observed in the control group of the corresponding RCT (Walsh 2012) |
| Timmermans 2012 | Mediterranean diet | Birth weight | MD -72.0 (-110.8 to -33.3) | MD 0.07 (0.03 to 0.11) | ES converted (g to kg) and from low vs. high adherence to high vs low, data not sufficient to convert MD to SMD |
| Tortosa 2007 | Mediterranean diet | HDL-Cholesterol (mmol/l) | NR | MD 0.01 (-0.05 to 0.06)<br>SMD 0.02 (-0.10 to 0.14) | ES generated with data presented in Table 1, MD to SMD, converted from mg/dl to mmol/l |
| Tortosa 2007 | Mediterranean diet | Metabolic syndrome | OR 0.20 (0.06 to 0.63) | RR 0.39 (0.14 to 0.81) | OR converted to RR with ACR=0.606; risk observed in the control group of the corresponding RCT (Salas-Salvadó 2008) |
| Tortosa 2007 | Mediterranean diet | Systolic blood pressure | NR | MD 0.80 (-0.84 to 2.44)<br>SMD 0.06 (-0.06 to 0.18) | ES generated with data presented in Table 1, MD to SMD |
| Tortosa 2007 | Mediterranean diet | Trigylcerides (mmol/l) | NR | MD -0.02 (-0.08 to 0.03)<br>SMD -0.05 (-0.17 to 0.08) | ES generated with data presented in Table 1, MD to SMD, converted from mg/dl to mmol/l |
| Wang 2015 | Dairy | Systolic blood pressure | NR | MD -0.60 (-0.86 to -0.34)<br>SMD -0.25 (-0.35 to -0.14) | ES generated with data provided in Table 2 |
| Wang 2016 | Folic acid | Low birthweight | NR | RR 0.35 (0.05 to 2.49) | ES generated with data provided in Table 2 |
| Wang 2016 | Folic acid | Birth weight | NR | MD 0.08 (0.15 to 0.00)<br>SMD 0.18 (0.00 to 0.35) | ES generated with data provided in Table 2, MD to SMD |
| Wen 2016 | Folic acid | Pre-eclampsia | OR 0.58 (0.33 to 1.02) | RR 0.59 (0.34 to 1.02) | OR converted to RR with ACR=0.027; risk observed in the control group of the corresponding RCT (Charles 2005) |
| Yang 2016 | Calcium | Cardiovascular disease mortality | Highest vs. lowest category:<br>RR 0.84 (0.74 to 0.94) | RR 0.85 (0.78 to 0.92) | dose-response per 1,000mg/day |
| Zschäbitz 2013 | Folic acid | Colorectal cancer | Highest vs. lowest category:<br>HR 1.01 (0.74 to 1.39) | RR 0.89 (0.25 to 3.20) | dose-response per 2,500µg/day; HR=RR |

CI: confidence interval; ES: effect estimate; HDL: high density lipoprotein; HPFS: Health Professionals Follow-up Study; HR: hazard ratio; ITT: intention-to-treat analysis; MACCE: Major adverse cardiac and cerebrovascular events; MD: mean difference; NHS: Nurses' Health Study; NHS II: Nurses' Health Study II; NR: not reported; OR: odds ratio; PUFA: polyunsaturated fatty acid; RR: risk ratio; SMD: standardised mean difference; SR: systematic review; TOHP II: Trials of Hypertension Prevention, Phase II

**Table S2** Reasons for exclusion

| Reference systematic review(s) | Intervention/ Exposure | Outcome | Reason |
|---|---|---|---|
| Abdelhamid 2018a/ Chowdhury 2014a | Omega-3 fatty acids | Cardiovascular mortality/ coronary heart disease mortality | Ineligible comparison: Intake+Supplementation vs. Intake |
| Abdelhamid 2018a/ Chowdhury 2014a | Omega-3 fatty acids | Cardiovascular disease/ coronary heart disease | Ineligible comparison: Intake+Supplementation vs. Intake |
| Abdelhamid 2018a/ Pan 2012 | α-Linolenic acid | Cardiovascular disease | Ineligible comparison: Intake+Supplementation vs. Intake |
| Abdelhamid 2018a/ Schlesinger 2019 | Omega-3 fatty acids/ Fish | Body weight/ Weight gain | Ineligible comparison: Intake+Supplementation vs. Intake |
| Abdelhamid 2018a/ Wan 2017 | Omega-3 fatty acids | All-cause mortality | Ineligible comparison: Intake+Supplementation vs. Intake |
| Abdelhamid 2018a/ Wei 2018 | α-Linolenic acid | Cardiovascular mortality/ Coronary heart disease mortality | Ineligible comparison: Intake+Supplementation vs. Intake |
| Abdelhamid 2018a/ Wei 2018 | α-Linolenic acid | Coronary heart disease | Ineligible comparison: Intake+Supplementation vs. Intake |
| Abdelhamid 2018b/ Li 2020 | Polyunsaturated fat/ Linoleic acid | All-cause mortality | Ineligible comparison: Intake+Supplementation vs. Intake |
| Abdelhamid 2018b/ Chowdhury 2014a | Polyunsaturated fat/ Omega-6 fatty acids | Coronary heart disease | Ineligible comparison: Intake+Supplementation vs. Intake |
| Adler 2014/ Aburto 2013 | Low-sodium | All-cause mortality | Overlap with Aburto 2013 “All-cause mortality” |
| Adler 2014/ Aburto 2013 | Low-sodium | Cardiovascular mortality | Overlap with Aburto 2013 “Cardiovascular disease”/ highly correlated outcome |
| Adler 2014/ Aburto 2013 | Low-sodium | Cardiovascular disease | Overlap with Aburto 2013 “Cardiovascular disease” |
| Adler 2014/ Leyvraz 2018 | Low-sodium | Systolic blood pressure | Ineligible comparison: Intake vs. Intake+Status |
| Adler 2014/ Leyvraz 2018 | Low-sodium | Diastolic blood pressure | Ineligible comparison: Intake vs. Intake+Status |
| Aguilar-Cordero 2020 | Vitamin D | Pre-eclampsia | Ineligible comparison: Supplementation vs. Status |
| Al-Khudairy 2017/ Aune 2018 | Vitamin C | Major cardiovascular events/ Cardiovascular disease | Ineligible comparison: Supplementation vs. Intake |
| Al-Khudairy 2017/ Aune 2018 | Vitamin C | Cardiovascular mortality | Ineligible comparison: Supplementation vs. Intake |
| Al-Khudairy 2017/ Aune 2018 | Vitamin C | All-cause mortality | Ineligible comparison: Supplementation vs. Intake |
| Alexander 2017 | EPA and DHA | Cardiovascular disease | Ineligible comparison: Intake+Supplementation vs. Intake |
| Amegah 2017 | Vitamin D | Gestational length | Ineligible comparison: Supplementation vs. Status |
| Avenell 2014/ Feng 2017 | Vitamin D | Hip fracture | Ineligible comparison: Supplementation vs. Status |
| Avenell 2014/ Feng 2017 | Vitamin D | Any fracture | Ineligible comparison: Supplementation vs. Status |
| Azad 2017 | Non-nutritive sweeteners | Body mass index | Ineligible comparison: Intake+Supplementation vs. Intake |
| Bjelakovic 2012/ Aune 2018 | ß-Carotene | All-cause mortality | Ineligible comparison: Supplementation vs. Intake |
| Bjelakovic 2012/ Aune 2018 | Vitamin E | All-cause mortality | Ineligible comparison: Supplementation vs. Intake |
| Bjelakovic 2012/ Aune 2018 | Vitamin C | All-cause mortality | Ineligible comparison: Supplementation vs. Intake |
| Bjelakovic 2012/ Aune 2018 | Vitamin A | All-cause mortality | Ineligible comparison: Supplementation vs. Intake |

| | | | |
|---|---|---|---|
| Bjelakovic 2014a/<br>Chowdhury 2014b | Vitamin D | All-cause mortality | Ineligible comparison:<br>Supplementation vs. Status |
| Bjelakovic 2014a/<br>Chowdhury 2014b | Vitamin D | Cardiovascular mortality | Ineligible comparison:<br>Supplementation vs. Status |
| Bjelakovic 2014a/<br>Han 2019 | Vitamin D | Cancer mortality | Ineligible comparison:<br>Supplementation vs. Status |
| Bjelakovic 2014b/<br>Han 2019 | Vitamin D | Cancer occurrence/<br>Cancer incidence | Ineligible comparison:<br>Supplementation vs. Status |
| Bjelakovic 2014b/<br>Zhang 2015 | Vitamin D | Lung cancer | Ineligible comparison:<br>Supplementation vs. Intake |
| Chowdhury 2012 | Omega-3 fatty acids | Cerebrovascular disease | Ineligible comparison:<br>Supplementation vs. Intake |
| Chowdhury 2014a | α-Linolenic acid | Coronary disease | Ineligible comparison:<br>Supplementation vs. Intake |
| Chowdhury 2014a | Omega-6 fatty acids | Coronary disease | Ineligible comparison:<br>Intake+Supplementation vs. Intake |
| Chowdhury 2014b | Vitamin D | All-cause mortality | Ineligible comparison:<br>Supplementation vs. Status |
| Chung 2011 | Vitamin D | Colorectal cancer | Ineligible comparison:<br>Supplementation vs. Status |
| Chung 2011 | Vitamin D | Breast Cancer | Ineligible comparison:<br>Supplementation vs. Status |
| Cormick 2015/<br>Jayedi 2019 | Calcium | Systolic blood pressure/<br>hypertension | Impossibility to convert one outcome to the other one |
| Cormick 2015/<br>Jayedi 2019 | Calcium | Diastolic blood pressure/<br>hypertension | Impossibility to convert one outcome to the other one |
| De-Regil 2015/<br>Feng 2015 | Folic acid | Congenital cardiovascular anomalies/ congenital heart defect | Impossibility to convert one outcome to the other one |
| El Dib 2015/<br>Fernandez-Cao 2019 | Zinc | HOMA-IR/<br>Type 2 diabetes | Impossibility to convert one outcome to the other one |
| Filippini 2020 | Green Tea | Prostate cancer | Ineligible comparison:<br>Supplementation vs. Intake |
| Filippini 2020 | Green Tea | Endometrial cancer | Ineligible comparison:<br>Supplementation vs. Intake |
| Hartley 2013/<br>Schwingshackl 2017 | Fruit and vegetable/<br>fruit | Systolic blood pressure/<br>Hypertension | Impossibility to convert one outcome to the other one |
| Hartley 2013/<br>Schwingshackl 2017 | Fruit and vegetable/<br>vegetable | Diastolic blood pressure/<br>hypertension | Impossibility to convert one outcome to the other one |
| Hartley 2016/<br>Schwingshackl 2017 | Fibre/ whole grain | Systolic blood pressure/<br>hypertension | Impossibility to convert one outcome to the other one |
| Hartley 2016/<br>Schwingshackl 2017 | Fibre/ whole grain | Diastolic blood pressure/<br>hypertension | Impossibility to convert one outcome to the other one |
| Hofmeyr 2018/<br>Newberry 2014 | Calcium | Pre-eclampsia | Ineligible comparison:<br>Supplementation vs. Intake |
| Hofmeyr 2018/<br>Newberry 2014 | Calcium | High blood pressure | Ineligible comparison:<br>Supplementation vs. Intake |
| Hooper 2012/<br>Noto 2013 | Low-fat/<br>High carbohydrates | Cardiovascular mortality | Ineligible comparison:<br>Intake+Supplementation vs. Intake |
| Hooper 2012/<br>Seidelmann 2018 | Low-fat/<br>High carbohydrates | All-cause mortality | Ineligible comparison:<br>Intake+Supplementation vs. Intake |
| Hooper 2012/<br>Zhu 2019 | Low-fat | Combined cardiovascular events/ cardiovascular disease | Ineligible comparison:<br>Intake+Supplementation vs. Intake |
| Hooper 2012/<br>Sartorius 2018 | Low-fat/<br>High carbohydrates | Body weight/<br>obesity | Impossibility to convert one outcome to the other one (moreover intervention in the RCTs meta-analysis too different from the intervention in the CSs meta-analysis) |

| | | | |
|---|---|---|---|
| Hooper 2015a/<br>Sartorius 2018 | Low-fat/<br>High carbohydrates | Body weight/<br>obesity | Impossibility to convert one outcome to the other one (moreover intervention in the RCTs meta-analysis too different from the intervention in the CSs meta-analysis) |
| Hooper 2015b/<br>de Souza 2015 | Low-saturated fat | Combined cardiovascular events/<br>coronary heart disease | Overlap with Hooper 2015b/<br>de Souza 2015 "Cardiovascular disease mortality"/ highly correlated outcome |
| Hooper 2018/<br>Chowdhury 2014a | Omega-6 fatty acids | Combined cardiovascular events/ coronary heart disease | Ineligible comparison:<br>Intake+Supplementation vs. Intake |
| Hooper 2018/<br>Li 2020 | Omega-6 fatty acids/<br>linoleic acid | All-cause mortality | Ineligible comparison:<br>Intake+Supplementation vs. Intake |
| Hooper 2018/<br>Li 2020 | Omega-6 fatty acids/<br>linoleic acid | Cardiovascular mortality | Ineligible comparison:<br>Intake+Supplementation vs. Intake |
| Jiang 2019 | Vitamin E | Age related cataract | Ineligible comparison:<br>Supplementation vs. Intake |
| Jiang 2019 | ß-carotene | Age related cataract | Ineligible comparison:<br>Supplementation vs. Intake |
| Jin 2012 | Total flavonoids | Colorectal adenoma/<br>colorectal cancer | RCTs analysed as cohort studies |
| Jin 2012 | Isoflavonoes | Colorectal adenoma/<br>colorectal cancer | RCTs analysed as cohort studies |
| Jin 2012 | Flavonols | Colorectal adenoma/<br>colorectal cancer | RCTs analysed as cohort studies |
| Johnston 2019 | Red meat | Cardiovascular mortality | Missing information: Included studies not cited |
| Johnston 2019 | Red meat | Type 2 diabetes | Missing information: Included studies not cited |
| Johnston 2019 | Red meat | Colorectal cancer | Missing information: Included studies not cited |
| Jonker 2020 | Folic acid | Small for gestational age | Ineligible comparison:<br>Supplementation vs.<br>Intake+Supplementation |
| Keats 2019/<br>Wolf 2017 | Micronutrients/<br>Vitamins | Preterm birth | Overlap with Wolf 2017 "Preterm birth" |
| Keats 2019/<br>Wolf 2017 | Micronutrients/<br>Vitamins | Low birthweight | Overlap with Jonker 2020 "Low birthweight" |
| Keats 2019/<br>Wolf 2017 | Micronutrients/<br>Vitamins | Small gestational age | Overlap with Jonker 2020 "Low birthweight"/ highly correlated outcome |
| Kelly 2017/<br>Schwingshackl 2017 | Whole grain | Systolic blood pressure/<br>hypertension | Impossibility to convert one outcome to the other one |
| Kelly 2017/<br>Schwingshackl 2017 | Whole grain | Diastolic blood pressure/<br>hypertension | Impossibility to convert one outcome to the other one |
| Kong 2014 | Vitamins | Gastric cancer | Ineligible comparison:<br>Supplementation vs. Intake |
| Lin 2019 | Vitamin E | Bladder Cancer | Ineligible comparison:<br>Supplementation vs. Intake |
| Martinez-Gonzalez 2014 | Olive Oil | Coronary Heart Disease | Ineligible comparison:<br>Intake+Supplementation vs. Intake |
| Martinez-Gonzalez 2014 | Olive Oil | Stroke | Ineligible comparison:<br>Intake+Supplementation vs. Intake |
| Mathew 2012/<br>Jiang 2019 | ß-Carotene | Cataract | Ineligible comparison:<br>Supplementation vs. Intake |
| Mathew 2012/<br>Jiang 2019 | Vitamin E | Cataract | Ineligible comparison:<br>Supplementation vs. Intake |
| Mathew 2012/<br>Jiang 2019 | Vitamin C | Cataract | Ineligible comparison:<br>Supplementation vs. Intake |

| | | | |
|---|---|---|---|
| Miller 2014 | Low-calorie sweeteners | Body weight | Impossible to convert the effect estimate (correlation coefficient) |
| Mocellin 2017 | Vitamin B6 | Cancer | Ineligible comparison: Supplementation vs. Intake |
| Palacios 2019/ Hu 2018 | Vitamin D | Gestational diabetes | Ineligible comparison: Supplementation vs. Status |
| Palacios 2019/ Tous 2020 | Vitamin D | Preterm birth | Ineligible comparison: Supplementation vs. Status |
| Palacios 2019/ Tous 2020 | Vitamin D | Birth length | Ineligible comparison: Supplementation vs. Status |
| Palacios 2019/ Tous 2020 | Vitamin D | Birth weight | Ineligible comparison: Supplementation vs. Status |
| Palacios 2019/ Tous 2020 | Vitamin D | Head circumference at birth | Ineligible comparison: Supplementation vs. Status |
| Palacios 2019/ Yuan 2019 | Vitamin D | Pre-eclampsia | Ineligible comparison: Supplementation vs. Status |
| Picasso 2019 | Vegetarian diet | Triglycerides | No cohort included: Brestrich 1996 is a non-randomised intervention study |
| Picasso 2019 | Vegetarian diet | HDL-Cholesterol | No cohort included: Brestrich 1996 is a non-randomised intervention study |
| Rees 2013a/ Kastorini 2011 | Healthy diet/ Mediterranean diet | Systolic blood pressure | Overlap with Kastorini 2011 "Systolic blood pressure" |
| Rees 2013a/ Kastorini 2011 | Healthy diet/ Mediterranean diet | Diastolic blood pressure | Overlap with Kastorini 2011 "Systolic blood pressure"/ highly correlated outcome |
| Rees 2013b/ Jayedi 2018 | Selenium | All-cause mortality | Ineligible comparison: Supplementation vs. Intake |
| Rees 2013b/ Xiang 2019 | Selenium | Cardiovascular mortality | Ineligible comparison: Supplementation vs. Status |
| Rees 2013b/ Zhang 2016a | Selenium | Combined cardiovascular events/ cardiovascular disease | Ineligible comparison: Supplementation vs. Status |
| Rees 2019/ Kastorini 2011 | Mediterranean diet | HDL | Overlap with Kastorini 2011 "HDL-Cholesterol" |
| Rees 2019/ Kastorini 2011 | Mediterranean diet | Triglycerides | Overlap with Kastorini 2011 "Triglycerides" |
| Rees 2019/ Kastorini 2011 | Mediterranean diet | Systolic blood pressure | Overlap with Kastorini 2011 "Systolic blood pressure" |
| Rees 2019/ Rosato 2019 | Mediterranean diet | Cardiovascular mortality | Overlap with Grosso 2015 "Cardiovascular disease" |
| Rees 2019/ Rosato 2019 | Mediterranean diet | Combined cardiovascular events/ cardiovascular disease | Overlap with Grosso 2015 "Cardiovascular disease" |
| Rutjes 2018/ Doets 2013 | B-Vitamins/ Vitamin B12 | Dementia + mild cognitive impairment/ dementia | Ineligible comparison: Supplementation vs. Intake |
| Rutjes 2018/ Goodwill 2017 | Vitamin D | Dementia/ dementia + mild cognitive impairment | Ineligible comparison: Supplementation vs. Status |
| Sayehmiri 2018 | Selenium | Prostate cancer | Ineligible comparison: Supplementation vs. Intake+Supplementation |
| Setien-Suero 2016 | Homocysteine | Mini Mental State Examination Score | Impossible to convert the effect estimate (correlation coefficient) |
| Sydenham 2012/ Zhang 2016b | Omega-3 fatty acids | Mini-mental state examination/ dementia | Impossibility to convert one outcome to the other one |
| Thorne-Lyman 2012 | Vitamin D | Birth weight | Ineligible comparison: Supplementation vs. Intake+Supplementation |
| Trikalinos 2012 | Vitamin E | Cardiovascular Mortality | Ineligible comparison: Supplementation vs. Intake+Supplementation |

| | | | |
|---|---|---|---|
| Usinger 2012/ Soedamah-Muthu 2012 | Fermented milk | Systolic blood pressure/ hypertension | Impossibility to convert one outcome to the other one |
| Usinger 2012/ Soedamah-Muthu 2012 | Fermented milk | Diastolic blood pressure/ hypertension | Impossibility to convert one outcome to the other one |
| Vinceti 2018a | Selenium | Cancer | Ineligible comparison: Supplementation vs. Status |
| Vinceti 2018a | Selenium | Cancer mortality | Ineligible comparison: Supplementation vs. Intake |
| Vinceti 2018b | Selenium | Type 2 Diabetes | Ineligible comparison: Supplementation vs. Intake |
| Yang 2020 | Calcium | Cardiovascular disease | Ineligible comparison: Supplementation vs. Intake |
| Yao 2017/ Ben 2014 | Fibre | Colorectal adenoma | overlap with Yao 2017/ Aune 2011 "Colorectal cancer" |
| Yao 2019 | Vitamin D | Any fracture | Ineligible comparison: Supplementation vs. Status |
| Zhang 2016 | Selenium | Cardiovascular disease | Ineligible comparison: Supplementation vs. Status |
| Zhang 2018 | Vitamin D | Gestational diabetes mellitus | Ineligible comparison: Supplementation vs. Status |
| Zhang 2018 | Vitamin D | Fasting plasma glucose | Impossible to convert the effect estimate (correlation coefficient) |
| Zhou 2017 | Vitamin D | Preterm birth | Ineligible comparison: Supplementation vs. Status |

DHA: docosahexaenoic acid; EPA: eicosapentaenoic acid; HDL: high density lipoprotein; HOMA-IR: homeostasis model assessment of insulin resistance

**Table S3** Characteristics of included randomised controlled trials

| Reference/ country/ acronym | Description of population | Age Gender | Description of intervention/ dose/ co-interventions | Description of comparator | Description of outcome/ Assessment | Duration, Follow-up (years) | Study design |
|---|---|---|---|---|---|---|---|
| Armitage 2010/<br>UK/<br>SEARCH | Male and female participants/<br>with history of myocardial infarction/<br>partly with treated hypertension (42%)/<br>without history of cancer/<br>blood cholesterol levels >135mg/dl under statin medication or >174mg/dl without | 18-80/<br>mean: 64.2<br><br>♀ 17% | Folic acid supplementation/<br>2mg/day/<br>with Vitamin B12 (1mg/day)/<br>with simvastatin (20 or 80mg/day) | Placebo/<br>with simvastatin (20 or 80mg/day) | Lung cancer/<br><br>self-reported, medical records, cancer and death registries | 6.7 | 2x2 factorial |
| Baron 2015/<br>US | Male and female participants/<br>generally healthy/<br>with recently diagnosed adenomas (removed within 120 days before enrolment, no known colorectal polyps remaining)/ without familial colorectal cancer syndromes or serious intestinal disease/ without history of kidney stones or hyperparathyroidism/<br>partly obese (36%) | 45-75/<br>mean: 58.1<br><br>♀ 37% | Vitamin D supplementation/<br>1,000IU/day/<br>partly with calcium carbonate (1,200mg/day) | Placebo/<br>partly with calcium carbonate (1,200mg/day) | Adverse events: urolithiasis/<br><br>self-reported, medical records | 3-5 | 2x2 factorial |
| Barr 2000/<br>US/ | Male and female participants/<br>generally healthy/<br>without diabetes, chronic or life threatening diseases, serious abnormality indicated in medical history or physical examination, hypertension | 55-85/<br>mean: 65.2<br><br>♀ 65% | Advice to increase skim or 1% milk intake/ add 3 eight-ounce servings to their usual consumption of dairy products | Advice to maintain usual diets/ including consumption of fewer than 1.5 servings/day | Changes in systolic blood pressure/<br><br>sphymanometer | 0.25 | parallel |
| Brough 2010/<br>UK | Female participants/<br>pregnant (5-18 weeks of gestation)/<br>with singleton pregnancy/<br>without chronic disease | 16-42/<br>mean: 28.2<br><br>♀ 100% | Multivitamin and micronutrient supplementation/<br>Pregnacare including 20mg/day Fe and 400µg/day folic acid/<br>daily until delivery | Placebo/<br>with folic acid (400µg/day) until 12 weeks of gestation | Preterm birth/<br>infants born before 37 weeks of gestation (ultrasound)/<br><br>clinical records | ≤18 weeks of gestation till delivery | parallel |
| Brunner 2011/<br>US/<br>WHI | Female participants/<br>generally healthy/ postmenopausal/<br>without hypercalcaemia, renal calculi, corticosteroid or calcitriol use/ mostly obese or overweight (73%) | 50-79/<br>mean: 62<br><br>♀ 100% | Vitamin D supplementation/<br>400IU/day/<br>with calcium (1,000mg/day) | Placebo | Breast cancer/<br><br>self-report, clinical visits, medical records, cancer screenings | 7 | parallel |

| | | | | | | | |
|---|---|---|---|---|---|---|---|
| Burr 1989/<br>UK/<br>DART | Male participants/<br>at high risk for CVD/ recovering from an myocardial infarction/<br>mostly smokers (62%)/<br>partly with hypertension (24%) or angina (22%)/ without diabetes | <70/<br>mean: 56.6<br><br>♀ 0% | Dietary advice to increase PUFA/ reduce fat intake to 30% of total energy and increase the polyunsaturated/saturated ratio to 10/<br>partly with advice on fish or fibre consumption | No dietary advice on fat intake/ partly with advice on fish or fibre consumption | Major cardiovascular events/ CVD death, non-fatal MI, stroke<br><br>self-report, confirmed by medical records, reports of general practitioners, death certificates of next of kin or register entry | 2 | 2x2x2 factorial |
| Chai 2012/<br>US | Female participants/<br>generally healthy/ postmenopausal/ not receiving hormone therapy and other pharmacologic agents | mean:<br>IG 55.6<br>CG 57.5<br><br>♀ 100% | Dried apple/<br>75g/day | Dried plum/<br>100g/day | Body weight/<br><br>direct measurement | 1 | parallel |
| Charles 2005/<br>UK/<br>Aberdeen Folate Supplementation Trial | Female participants/<br>pregnant (~17 weeks of gestation)/<br>partly smoking (42%) | mean: 25.9<br><br>♀ 100% | Folic acid supplementation/<br>200µg/day or 5mg/day | Placebo | Pre-eclampsia/<br>hypertension with a diastolic blood pressure >90 mmHg on two separate occasions, with or without oedema, with albuminuria exceeding 0.25g/l/<br><br>obstetric records | <30 weeks of gestation till delivery | parallel (3 arms) |
| Christian 2003/<br>Nepal | Female participants/<br>pregnant (~11 weeks of gestation) | reproductive age<br><br>♀ 100% | Folic acid supplementation/<br>400g/day/<br>with vitamin A (1,000µg/day) | Vitamin A supplementation/<br>1,000µg/day | Low birthweight/<br><2,500g measured within 72 hours of birth | ~11 weeks of gestation till delivery | cluster |
| | | | | | Mean birthweight/<br><br>direct measurement | | |
| Czeizel 1994/<br>Hungary/<br>HOFPP | Female participants/<br>planning to get pregnant/<br>without delayed conception or infertility | reproductive age/<br>mean: 26.9<br><br>♀ 100% | Folic acid supplementation/<br>800µg/day/<br>multivitamins including 12 vitamins, 4 minerals and 3 trace elements/ one tablet/day/<br>one month before conception until 12 weeks of pregnancy | Trace element supplementation/<br>equal to trace element components of the intervention group/ one tablet a day | Neural-tube defect/<br><br>self-report, confirmed by physicians, home visits | 1 | parallel |
| Czeizel 1998/<br>Hungary/<br>HOFPP | Female participants/<br>planning to get pregnant/<br>without delayed conception or infertility | reproductive age/<br>mean: 26.9<br><br>♀ 100% | Multivitamin supplementation/<br>rich in folic acid (800µg/day)/<br>including 12 vitamins, 4 minerals and 3 trace elements/<br>1-3 months before conception | Trace element supplementation/<br>equal to trace element components of the intervention group/ one tablet a day | Major congenital abnormality: cardiovascular defects/<br><br>hospital records, medical records, autopsy reports | 1 | parallel |

| | | | | | | | |
|---|---|---|---|---|---|---|---|
| de Lorgeril 1998/ France/ Lions Diet Heart Study | Male and female participants/ with history of myocardial infarction (within 6 months of enrolment)/ without heart failure and hypertension and inability to complete an exercise test due to recurrent angina, ventricular arrhythmias, or atrioventricular block | <70/ mean: 53.5<br><br>♀ 10% | Advice by to adopt a Mediterranean-type diet/ experimental canola oil-based margarine rich in oleic and α-linolenic acids | No dietary advice apart from usual care/ follow the dietary advice given by their attending physicians (not involved in the study) and close to the step 1 prudent diet of the American Heart Association | Cancer mortality/<br><br>NR | IG 3.7<br>CG 3.9 | parallel |
| Esposito 2009/ Italy | Male and female participants/ with newly diagnosed type 2 diabetes/ overweight/ sedentary (<1 hour of physical activity/week) | 30-75/ mean: 52.2<br><br>♀ 51% | Dietary counselling to follow low-carbohydrate Mediterranean diet/ 30-50g of olive oil/ energy restriction to 1,500kcal/day for women and 1,800kcal/day for men/ guidance on increasing level of physical activity | Dietary counselling to follow low-fat diet/ based on American Heart Association guidelines: rich in whole grains and restricted additional fats, sweets, and high-fat snacks/ energy restriction to 1,500kcal/day for women and 1,800kcal/day for men/ guidance on increasing level of physical activity | HDL-Cholesterol/ in mmol/l/<br><br>laboratory analyses | 4 | parallel |
| | | | | | Triglycerides/ in mmol/l/<br><br>laboratory analyses | | |
| | | | | | Systolic blood pressure/<br><br>NR | | |
| Estruch 2018/ Spain/ PREDIMED | Male and female participants/ without CVD, but with type 2 diabetes mellitus or ≥3 cardiovascular risk factors/ community-dwelling | 55-80/ mean: 67<br><br>♀ 57% | Dietary counselling to follow Mediterranean diet/ supplementation with extra-virgin olive oil (4 tablespoons/ day) or nuts (30g of mixed nuts/day) | Dietary counselling to reduce intake of all types of fat/ leaflet explaining the low-fat diet (first 3 years); later individual visits to dieticians and group sessions every 3 months | All-cause mortality/<br><br>next of kin, physicians, medical records, register entry | 4.8 (median) | parallel (3 arms) |
| | | | | | Cardiovascular events/ myocardial infarction, stroke, death from cardiovascular causes/<br><br>self-report, physicians report, medical records, register entry | | |
| | | | Nuts/ provision of 30g of mixed nuts/day (15g of walnuts, 7.5g of hazelnuts, and 7.5g of almonds)/ main intervention: dietary counselling to follow Mediterranean diet | Dose-response: per 4 servings (28.4g)/ week of nuts | Myocardial infarction/ fatal and nonfatal events/<br><br>self-report, physicians report, medical records, register entry | | |
| | | | | | Stroke/<br><br>self-report, physicians report, medical records, register entry | | |

| | | | | | | | |
|---|---|---|---|---|---|---|---|
| Gaziano 2009/<br>US/<br>PHS II | Male participants/<br>physicians/<br>without history of prostate cancer, cirrhosis, active liver disease, serious illness/ not on anticoagulants | ≥50/<br>mean: 64.3<br><br>♀ 0% | Vitamin C supplementation/<br>500mg/day ascorbic acid/<br>partly with vitamin E (400IU on alternate days)/ partly with β-carotene (50mg on alternate days)/ partly with multivitamin | Placebo/<br>partly with vitamin E/<br>partly with β-carotene/<br>partly with multivitamin | Prostate cancer incidence/<br><br>self-reported, medical and clinical records | 8 | 2x2x2x2 factorial |
| | | | Vitamin E supplementation/<br>400IU α-tocopherol on alternate days/<br>partly with vitamin C (500mg/day)/ partly with β-carotene (50mg on alternate days)/ partly with multivitamin | Placebo/<br>partly with vitamin C/<br>partly with β-carotene/<br>partly with multivitamin | | | |
| Heinonen 1998/<br>Finland/<br>ATBC | Male participants/<br>smokers with ≥5 cigarettes/day/<br>without prior cancer, serious disease | 50-69/<br>mean: 57.1<br><br>♀ 0% | β-carotene supplementation/<br>20mg/day/<br>partly with α-tocopherol (50mg/day) | Placebo/<br>partly with α-tocopherol (50mg/day) | Prostate cancer incidence/<br><br>cancer and death registries, medical records | 6.1 (median) | 2x2 factorial |
| Hollis 2011/<br>US | Female participants/<br>pregnant (<16 weeks of gestation)/<br>singleton pregnancy/<br>without pre-existing calcium or parathyroid conditions or who required chronic diuretic or cardiac medication therapy | 17-44/<br>mean: 27<br><br>♀ 100% | Vitamin D supplementation/<br>1,600IU or 3,600IU/day depending on 25(OH)D level/<br>with multivitamin (containing 400IU/day Vitamin D3) | Placebo/<br>with multivitamin (containing 400IU/day Vitamin D3) | Pre-eclampsia or gestational hypertension/<br><br>self-report (questionnaire), medical records | 12-16 weeks of gestation till delivery | parallel (3 arms) |
| Howard 2006/<br>US | Female participants/<br>postmenopausal/<br>without history of cancer (except non-melanoma skin cancer) in the last 10 years, without type 1 diabetes/<br>for outcome: CVD mortality: without history of CVD | 50-79/<br>mean: 62.3<br><br>♀ 100% | Low-fat diet/<br>promote dietary change with the goals of reducing intake of total fat to 20% of energy intake by increasing intake of vegetables and fruits to at least 5 servings daily and of grains to at least 6 servings daily/<br>18 group sessions in the first year and quarterly maintenance sessions thereafter, led by nutritionists | Usual diet/<br>received a copy of the Dietary Guidelines for Americans and other health-related materials | All-cause mortality/<br><br>medical update questionnaire, medical records, death certificate | 8.1 | parallel |
| | | | | | Cardiovascular disease mortality/<br><br>medical update questionnaire, medical records, death certificate | | |
| Hsia 2007/<br>US/<br>WHI | Female participants/<br>postmenopausal/<br>partly with hypertension (30%), diabetes (6%), CVD (5%) at baseline | 50-79/<br>mean: 62.4<br><br>♀ 100% | Calcium supplementation/<br>1,000IU/day/<br>with Vitamin D3 (400IU/day) | Placebo | Coronary heart disease mortality/<br><br>medical records | 7 | parallel |

| | | | | | | | |
|---|---|---|---|---|---|---|---|
| Jackson 2006/<br>US/<br>WHI (subsample) | Female participants/<br>generally healthy/<br>postmenopausal/<br>without hypercalcemia, renal calculi, corticosteroid use, and calcitriol use | 50-79/<br>mean: 62.4<br><br>♀ 100% | Calcium supplementation/<br>500mg/day as calcium carbonate/<br>with Vitamin D2 (200IU/day) | Placebo | Fractures/<br>clinical fractures without ribs, sternum, skull, face, fingers, toes, cervical vertebrae/<br><br>medical records | 7 | parallel |
| Karp 2013/<br>US, Canada/<br>ECOG 5597 | Male and female participants/<br>with complete resection of stage I non-small-cell lung cancer/<br>without sign of new or recurrent (lung) cancers, without history of cancer history in past 5 years except localised non-melanoma skin cancer | 24-93/<br>mean: 66<br><br>♀ 51% | Selenium supplementation/<br>200µg/day as selenium yeast | Placebo | Oesophagus cancer/<br><br>pathology report/<br>physical exam, medical history, blood tests, chest x-ray/ central review of tumour sample | 4 | parallel |
| Kirke 1992/<br>Ireland | Female participants/<br>not pregnant at time of recruitment/<br>having had a baby with a neural tube defect | NR/<br>mean: 31.3<br><br>♀ 100% | Multivitamin supplementation/<br>periconceptional/<br>partly with folic acid (0.36mg/day) | Folic acid alone/<br>0.36mg/day | Stillbirth/<br><br>NR | ≥2 months before conception till delivery | parallel<br>(3 arms) |
| Lin 2009/<br>US/<br>WACS | Female participants/<br>health professionals/<br>postmenopausal or no intention to become pregnant/<br>at high risk of CVD: with history of CVD or with ≥3 coronary risk factors/<br>without history of cancer,<br>active liver disease or cirrhosis, chronic kidney failure, not current users of anticoagulants | ≥40/<br>mean: 60.4<br><br>♀ 100% | Vitamin C supplementation/<br>500mg/day/<br>partly with Vitamin E (600IU on alternate days)/<br>partly with ß-carotene (50mg on alternate days) | Placebo/<br>partly with Vitamin E/<br>partly with ß-carotene | Breast cancer/<br><br>self-report, next of kin, postal authorities, register entry, confirmed by pathology or cytology reports, otherwise clinical and radiological or laboratory marker evidence | 9.4 | 2x2x2 factorial |
| | | | | | Colorectal cancer/<br><br>self-report, next of kin, postal authorities, register entry, confirmed by pathology or cytology reports, otherwise clinical and radiological or laboratory marker evidence | | |
| | | | | | Lung cancer/<br><br>self-report, next of kin, postal authorities, register entry, confirmed by pathology or cytology reports, otherwise clinical and radiological or laboratory marker evidence | | |

| | | | | | | | |
|---|---|---|---|---|---|---|---|
| Lippman 2009/<br>US, Canada, Puerto Rico/<br>SELECT | Male participants/<br>generally healthy/<br>without prior diagnosis of prostate cancer/ serum prostate-specific antigen level of ≤4ng/ml/<br>digital rectal examination not suspicious for prostate cancer | ≥50 (African Americans)<br>≥55 (others)<br>mean: 62.6<br><br>♀ 0% | Selenium supplementation/<br>200µg/day from<br>L-selenomethionine | Placebo | Colorectal cancer/<br><br>self-report, confirmed by medical records and pathology reports | 5.46 (median) | 2x2 factorial (arms with Vitamin E not considered) |
| | | | | | Prostate cancer/<br><br>self-report, confirmed by medical records and pathology reports | | |
| Maki 2010/<br>US | Male and female participants/<br>generally healthy/<br>overweight or obese (BMI 25 to 40)/<br>without history of cardiac, renal, hepatic, endocrine, pulmonary, biliary, pancreatic, gastrointestinal or neurologic disorders, or cancer in the past 2 years | 20-65/<br>mean: 48.8<br><br>♀ ~78% | Provision of whole grains/<br>two portions/day/<br>whole grain oat ready-to-eat cereal as part of a dietary program for weight loss (including physical activity) | Provision of low fibre foods/<br>energy-matched low-fibre foods as part of a dietary program for weight loss | Body weight/<br><br>NR/ repeated measurements | 0.23 | parallel |
| Maraini 2008/<br>Italy/<br>CTNS | Male and female participants/<br>with early (70%) or no (30%) cataract | 55-75/<br>mean: 68<br><br>♀ 45% | Multivitamin and mineral supplementation/<br>including 13 vitamins and 17 minerals | Placebo | Cortical opacity/<br><br>annual lens photographs | 9 | parallel |
| | | | | | Nuclear opacity/<br><br>annual lens photographs | | |
| | | | | | Posterior subcapsular opacity/<br><br>annual lens photographs | | |
| Merchant 2005/<br>Tanzania | Female participants/<br>pregnant (12-27 weeks of gestation)/<br>HIV-infected | mean: 24.7<br><br>♀ 100% | Multivitamin supplementation/<br>including e.g. 0.8mg folic acid/<br>with folate (5mg), ferrous sulphate (400mg), chloroquine phosphate (500mg)/<br>partly with Vitamin A (30mg ß-carotene plus 5,000IU preformed vitamin A) | Placebo/<br>with folate (5mg), ferrous sulphate (400mg), chloroquine phosphate (500mg)/<br>partly with Vitamin A (30mg ß-carotene plus 5,000IU preformed vitamin A)/ | Gestational hypertension/<br>systolic RR≥140 mmHg or diastolic RR≥90 mmHg at any time during pregnancy<br><br>mercury sphygmomanometer, monthly measurements | <27 weeks till 3 months postpartum | 2x2 factorial |

| | | | | | | | |
|---|---|---|---|---|---|---|---|
| Meyer 2005/<br>France/<br>SU.VI.MAX | Male participants/<br>without severe health problems/<br>without prostate cancer | 45-60/<br>mean: 51.3<br><br>♀ 0% | Multivitamin supplementation/<br>including vitamin C (120mg/day), Vitamin E (30mg/day), β-carotene (6mg/day), selenium (100µg/day), zinc (20mg/day) | Placebo | Prostate cancer/<br><br>PSA test, rectal examination, transrectal ultrasound, prostate biopsy, pathology report | IG 8.8, CG 9.0 (median) | parallel |
| Moses 2014/<br>Australia/<br>PREGGIO | Female participants/<br>pregnant (<20 weeks of gestation)/<br>singleton pregnancy/<br>without known diabetes or previous gestational diabetes, special dietary needs, the presence of medical conditions that could compromise the metabolic status, or the use of medications that were likely to influence body weight | ≥18/<br>mean: 30<br><br>♀ 100% | Dietary counselling to follow a low–glycaemic index diet/<br>dietary education and provision of set of booklets that included information of choices for and serving sizes of carbohydrate-rich foods, with specific information on low–glycaemic index alternatives for relevant food groups | Dietary counselling to follow a conventional healthy diet/<br>dietary education and provision of set of booklets that included information on recommended foods and serving sizes as noted in the Australian Guide to Healthy Eating, not given any guidance on glycaemic index diet | Small for gestational age/<br>Birth weight <10th percentile<br><br>Medical records | <20 weeks till delivery | parallel |
| Pan 1997/<br>China/<br>The Da Qing IGT and Diabetes Study | Male and female participants/<br>with impaired glucose tolerance | ≥25/<br>mean: 45<br><br>♀ 47% | Dietary counselling to follow a healthy diet/<br>encouraged to consume more vegetables, control intake of alcohol, and reduce intake of simple sugars/<br>individual and group sessions | General information about diabetes and impaired glucose tolerance/<br>information brochures with general instructions for diet and/or increased leisure physical activities | All-cause mortality/<br><br>NR<br><br>Type 2 Diabetes/<br><br>repeated fasting glucose, oral glucose tests tolerance test | 6 | cluster<br>(4 arms)<br><br>(groups with exercise intervention were not considered) |
| Reid 2007/<br>UK | Female participants/<br>generally healthy/<br>not pregnant or lactating/ normal weight (BMI<25)/ not dieting or exercising to lose weight/<br>without dislike of popular sweet carbonated drinks/ | 20-55/<br>mean: 31.8<br><br>♀ 100% | Provision of soft drinks with sucrose sweetener/<br>partly labelled as "diet drinks" or misinformed (wrong label)/<br>receiving 4x250ml bottle/day (total: 1,800kJ) | Provision of soft drinks with artificial sweetener (aspartame)/<br>partly labelled as "diet drinks" or misinformed (wrong label)/<br>receiving 4x250ml bottle/day (total: 1,800kJ) | Body weight change/<br><br>digital scale | 0.1 | 2x2x2 factorial |
| Riggs 1998/<br>US | Female participants/<br>elderly/ postmenopausal for ≥10 years/<br>fully ambulatory/<br>without history of osteoporotic or vertebral fracture, renal lithiasis, impaired renal function, hypercalcemia or hypercalciuria | 61-70/<br>mean: 66.3<br><br>♀ 100% | Calcium supplementation/<br>1,600mg/day as citrate | Placebo | Side effects: renal stones/<br><br>NR | 4 | parallel |

| | | | | | | | |
|---|---|---|---|---|---|---|---|
| Salas-Salvadó 2008/<br>Spain/<br>PREDIMED<br>(subsample) | Male and female participants/<br>without CVD, but with type 2 diabetes or ≥3 cardiovascular risk factors/<br>partly with metabolic syndrome (61%)/<br>community-dwelling | 55-80/<br>mean: 67.4<br><br>♀ 54% | Dietary counselling to follow Mediterranean diet/<br>supplementation with extra-virgin olive oil (1l/week) or nuts (30g of mixed nuts/day) | Dietary counselling to reduce intake of all types of fat/<br>general oral and written recommendations/ leaflet explaining the low-fat diet | Metabolic syndrome prevalence/<br>according to updated Adult Treatment Panel III criteria/<br><br>anthropometric assessment (stadiometer, oscillometer, tape), laboratory analyses | 1 | parallel<br>(3 arms) |
| Salas-Salvadó 2014/<br>Spain/<br>PREDIMED<br>(subsample) | Male and female participants/<br>without CVD or type 2 diabetes at baseline, but with ≥3 cardiovascular risk factors/ community-dwelling | 50-80/<br>mean: 66.6<br><br>♀ 62% | Dietary counselling to follow Mediterranean diet/<br>supplementation with extra-virgin olive oil (50ml/day) or nuts (30g of mixed nuts/day) | Dietary counselling to reduce intake of all types of fat/<br>leaflet explaining the low-fat diet (first 3 years); later individual visits to dieticians and group sessions every 3 months | Type 2 Diabetes/<br>according to the American Diabetes Association criteria/<br><br>routine biochemical analyses, medical reports | 4.1 (median) | parallel<br>(3 arms) |
| | | | Extra-virgin olive oil/<br>provision of 50ml/day/ main intervention: dietary counselling to follow Mediterranean diet | Dose-response: per 10g/daily increase in olive oil | | | |
| | | | Nuts/ provision of 30g of mixed nuts/day (15g of walnuts, 7.5g of hazelnuts, and 7.5g of almonds)/<br>main intervention: dietary counselling to follow Mediterranean diet | Dose-response: per 4 servings (28.4g)/ week of nuts | | | |
| Schatzkin 2000/<br>US/<br>Polyp Prevention Trial | Male and female participants/<br>with one or more histologically confirmed colorectal adenomas (removed within six months before randomisation)/<br>no history of colorectal cancer, surgical resection of adenomas, bowel resection, the polyposis syndrome, or inflammatory bowel disease | ≥35/<br>mean: 61<br><br>♀ 35% | Dietary counselling to adopt a diet that was low in fat, high in fibre, fruits, and vegetables/<br>goal: 18g of dietary fibre per 1,000 kcal, 20% total calories from fat, 3.5 servings of fruits and vegetables per 1,000 kcal | Usual diet/<br>standard brochure on healthy eating, general dietary guidelines from the National Dairy Council/<br>no additional nutritional or behavioural information | Colorectal cancer<br><br>colonoscopy, review by central pathologists | 4 | parallel |

| | | | | | | | |
|---|---|---|---|---|---|---|---|
| Sesso 2012/<br>US/<br>PHS II | Male participants/<br>physicians/<br>partly with history of CVD (5%) and cancer (9.0%)/<br>without history of cirrhosis, active liver disease, not taking anticoagulants or reported serious illness that might preclude participation<br><br>for outcomes: myocardial infarction and stroke: without CVD | ≥50/<br>mean: 64.3<br><br>♀ 0% | Multivitamin and micronutrient supplementation/<br>including 13 vitamins and 17 minerals/<br>partly with vitamin E (400IU on alternate days)/ partly with Vitamin C (500mg/day)/ partly with β-carotene (50mg on alternate days) | Placebo/<br>partly with vitamin E/<br>partly with vitamin C/<br>partly with β-carotene | Cardiovascular death/<br><br>self-reported, next of kin, confirmed by medical records including autopsy records, death certificates, register entry | 11.3 | 2x2x2x2 factorial |
| | | | | | Myocardial infarction/<br><br>self-reported, next of kin, confirmed by medical records including autopsy records, death certificates, register entry | | |
| | | | | | Stroke/<br><br>self-reported, next of kin, confirmed by medical records including autopsy records, death certificates, register entry | | |
| Sichieri 2009/<br>Brazil | Male and female participants/<br>children/<br>fourth graders from public schools/<br>mostly from families of low socio-economic level | 9-12/<br>mean: 10.9<br><br>♀ 52.8 | Behavioural intervention to reduce intake of sugar sweetened beverages/<br>healthy lifestyle education programme with simple messages encouraging water consumption instead of sugar sweetened beverages | General health sessions and printed advice regarding healthy diets | Change in BMI/<br><br>portable scale | 0.67 | cluster |
| TOHP II 1997/<br>US/<br>TOHP II | Male and female participants/<br>generally healthy/<br>moderately overweight/<br>with high-normal diastolic RR,<br>not taking antihypertensive drugs | 30-54/<br>mean: 44<br><br>♀ 33.5 | Dietary advice to reduce sodium intake/<br>goal: mean sodium intake <70mmol/day/<br>partly with weight loss intervention | Usual Care/<br>no active intervention or weight loss alone | Other effects: number of deaths/<br><br>NR | 3-4 | 2x2 factorial |
| Toledo 2018/<br>Spain/<br>PREDIMED | Female participants/<br>without CVD, but with type 2 diabetes mellitus or ≥3 cardiovascular risk factor/ without prior diagnosis of breast cancer or probable breast tumours | 55-80/<br>mean: 67.7<br><br>♀ 100% | Dietary counselling to follow Mediterranean diet/<br>supplementation with extra-virgin olive oil (1l/week) or nuts (30g of mixed nuts/day) | Dietary counselling to reduce intake of all types of fat/<br>leaflet explaining the low-fat diet (first 3 years); later individual visits to dieticians and group sessions every 3 months | Breast cancer/<br><br>medical records, death certificates | 4.8 (median) | parallel |

| | | | | | | | |
|---|---|---|---|---|---|---|---|
| Walsh 2012/<br>Ireland/<br>ROLO | Female participants/<br>generally healthy/<br>pregnant (~13 weeks of gestation)/<br>singleton pregnancy/<br>secundigravid and having previously delivered a macrosomic infant weighing greater than 4 kg | ≥18/<br>mean: 32<br><br>♀ 100% | Dietary counselling to follow a healthy diet/low-glycaemic index diet/<br>dietary education session at ~16 weeks, and at 28 and 34 weeks/<br>written resources about low-glycaemic index diet | Routine antenatal care/<br>no formal dietary advice or specific advice about gestational weight gain | Preterm birth/<br>delivery at <37 weeks of gestation<br><br>Recording at delivery | <18 weeks of gestation till delivery | parallel |
| | | | | | Birth weight<br><br>Recording at delivery | | |
| | | | | | Gestational diabetes/<br>according to American Diabetes Association criteria<br><br>Repeat fasting blood glucose and glucose challenge testing at 28 weeks' gestation | | |
| Whelton 1998/<br>US/<br>TONE | Male and female participants/<br>elderly with hypertension, receiving antihypertensive medication/<br>partly overweight or obese (60%) | 60-80/<br>mean: 66.5<br><br>♀ 48% | Dietary counselling to restrict sodium intake/<br>goal: achieving and maintaining a 24-hour dietary sodium intake of 80mmol (1,800mg) or less;<br>withdrawal of antihypertensive medication after 3 months | Usual Care/<br>no study-related counselling in lifestyle change techniques/<br>meetings on topics unrelated to the goals of the trial;<br>withdrawal of antihypertensive medication after 3 months | Clinical cardiovascular disease complication/<br>myocardial infarction, angina, congestive heart failure, stroke, coronary artery bypass surgery, or coronary artery angioplasty/<br><br>Self-report, next of kin, physicians, review of physician records | 2.4 (median) | partial 2x2 factorial (groups with weight loss intervention were not considered) |
| Zhang 2008/<br>US/<br>WAFACS | Female participants/<br>health professionals/ postmenopausal or no intention of becoming pregnant/<br>at high risk of CVD: with history of CVD or with ≥3 coronary risk factors/<br>without history of cancer or any serious non-CVD illness | ≥40/<br>mean: 62.8<br><br>♀ 100% | Folic acid supplementation/<br>2.5mg/day/<br>with Vitamin B6 (50mg/day) and B12 (1mg/day)/<br><br>*parent trial (WACS):*<br>partly with vitamin C (500mg/day), vitamin E (600IU on alternate days), β-carotene (50mg on alternate days) | Placebo/<br><br>*parent trial (WACS):*<br>partly with vitamin C (500mg/day), vitamin E (600IU on alternate days), β-carotene (50mg on alternate days) | Breast Cancer/<br><br>self-report, next of kin, confirmed by medical records including autopsy reports, death certificates, register entry | 7.3 (mean)/<br>+1 additional year of follow-up | parallel |
| | | | | | Colon and rectum cancer/<br><br>self-report, next of kin, confirmed by medical records including autopsy reports, death certificates, register entry | | |

| | | | | | | | |
|---|---|---|---|---|---|---|---|
| | | | | | Pancreatic cancer/<br><br>self-report, next of kin, confirmed by medical records including autopsy reports, death certificates, register entry | | |

ATBC study: Alpha-Tocopherol, Beta-Carotene Cancer Prevention Study; BMI: body mass index; CG: control group; CTNS: Italian-American Clinical Trial of Nutritional Supplements and Age-Related Cataract; CVD: cardiovascular disease; DART: Diet and Reinfarction Trial; ECOG: Eastern Cooperative Oncology Group; HDL: high density lipoprotein; HIV: human immunodeficiency virus; HOFPP: Hungarian Optimal Family Planning programme; IG: intervention group; MI: myocardial infarction; NR: not reported; PHS II: Physicians' Health Study II; PREDIMED: Prevención con Dieta Mediterránea; PREGGIO: Pregnancy and Glycemic Index Outcomes study; PSA: prostate-specific antigen; PUFA: polyunsaturated fatty acid; ROLO: Randomised controlled trial of low glycaemic index diet in pregnancy to prevent macrosomia; SEARCH: Study of the Effectiveness of Additional Reductions in Cholesterol and Homocysteine; SELECT: Selenium and Vitamin E Cancer Prevention Trial; SU.VI.MAX: The supplémentation en vitamines et minéraux antioxydants trial; TOHP II: Trials of Hypertension Prevention, Phase II; TONE: Trial of Nonpharmacologic Interventions in the Elderly; UK: United Kingdom; US: United States; WACS: Women's Antioxidant Cardiovascular Study; WAFACS: Women´s Antioxidant and Folic Acid Cardiovascular Study; WHI: Women´s Health Initiative

**Table S4** Characteristics of included cohort studies

| Reference/ country/ acronym | Description of population | Age Gender | Description of exposure/ assessment | Description of comparison | Description of outcome/ Assessment | Follow-up (years) | Study design |
|---|---|---|---|---|---|---|---|
| Alvarez-Alvarez 2017/<br>Spain/<br>SUN | Male and female population/<br>university graduates | ≥35/<br>mean: 38.2<br><br>♀ 60% | Mediterranean diet/<br>validated FFQ/ at baseline/<br>MED-score | High vs. Low/<br>6-9 vs. ≤2 points | All-cause mortality/<br><br>Next of kin, work's associates, postal authorities, register entry | 10.3 | prospective |
| Bailey 2015/<br>US/<br>NHANES III | Male and female population/<br>general population/<br>without history of CVD or chronic kidney disease/<br>partly with hypertension (36%), diabetes (11%) or hyperlipidaemia (30%)/ partly smoking (30%) | ≥40/<br>mean: 57<br><br>♀ 54% | Multivitamin and minerals supplementation/<br>≥3 vitamins and ≥1 minerals/<br>interviews, showing containers of supplements/ at baseline | High vs. Low/<br>>3 years of use vs. non-use | Cardiovascular mortality/<br><br>Probabilistic match to the death certificate records from the National Death Index | 18.7 | prospective |
| Bao 2013/<br>US/<br>HPFS | Male population/<br>health professionals/<br>without CVD, diabetes or cancer | 40-75/<br><br>♀ 0% | Nuts intake/<br>validated FFQ/ at baseline, updated every 2-4 years | Dose-response/<br>per 4 servings (28.4g) per week | Heart disease death/<br><br>National Death Index, postal authorities, next of kin/ review of death certificates, medical reports | 24 | prospective |
| Bao 2013/<br>US/<br>NHS | Female population/<br>nurses/<br>without CVD, diabetes or cancer/<br>mostly postmenopausal (60-68%) | 34-59/<br><br>♀ 100% | Nuts intake/<br>validated FFQ/ at baseline, updated every 2-4 years | Dose-response/<br>per 4 servings (28.4g) per week | Heart disease death/<br><br>National Death Index, postal authorities, next of kin/ review of death certificates, medical reports | 30 | prospective |
| Bernstein 2012/<br>US/<br>HPFS | Male population/<br>health professionals/<br>without CVD, diabetes or cancer | 40-75/<br><br>♀ 0% | Nuts intake/<br>validated FFQ/ at baseline, updated every 2-4 years | Dose-response/<br>per 4 servings (28.4g) per week | Stroke/<br><br>Self-report/ confirmed by medical records, autopsy reports/ state vital records, National Death Index, postal authorities, next of kin | 22 | prospective |
| Bernstein 2012/<br>US/<br>NHS | Female population/<br>nurses/<br>without CVD, diabetes or cancer | 30-55/<br><br>♀ 100% | Nuts intake/<br>validated FFQ/ at baseline, updated every 2-4 years | Dose-response/<br>per 4 servings (28.4g) per week | Stroke/<br><br>Self-report/ confirmed by medical records, autopsy reports/ state vital records, National Death Index, postal authorities, next of kin | 26 | prospective |

| | | | | | | | |
|---|---|---|---|---|---|---|---|
| Bertoia 2015/<br>US/<br>NHS | Female population/<br>nurses/<br>without chronic disease (e.g. CVD, diabetes, cancer) | 40-65/<br>mean: 48.7<br><br>♀ 100% | Apples and pears intake/<br>validated FFQ/<br>at baseline, update every 4 years | Dose-response:<br>per serving/day | Body weight change/<br><br>Self-report | 24 | prospective |
| Bertoia 2015/<br>US/<br>NHS II | Female population/<br>nurses/<br>without chronic disease (e.g. CVD, diabetes, cancer) | 27-44/<br>mean: 36.4<br><br>♀ 100% | Apples and pears intake/<br>validated FFQ/<br>at baseline, update every 4 years | Dose-response:<br>per serving/day | Body weight change/<br><br>Self-report | 16 | prospective |
| Buckland 2009/<br>Spain/<br>EPIC-Spain | Male and female population/<br>generally healthy/<br>mostly active blood donors (55-60%) | 29-69/<br>mean: 49.3<br><br>♀ 62% | Mediterranean diet/<br>validated dietary history questionnaire/ interview/<br>rMED score | High vs. Low/<br>11-18 vs. 0-6 points | Coronary heart disease/<br><br>Self-report, hospital discharge database, myocardial infarction registry, mortality registry/<br>review of medical records and medico-legal necropsy reports | 10.4 | prospective |
| Buckland 2013/<br>various European countries/<br>EPIC | Female population/<br>general population/ without history of cancer/ partly postmenopausal (46%) | 35-70 (mostly)/<br>mean: 50.8<br><br>♀ 100% | Mediterranean diet/<br>validated FFQ or diet history questionnaires/ country-specific/ interview/<br>arMED score | High vs. Low/<br>11-18 vs. 0-6 points | Primary invasive breast cancer/<br><br>Cancer registries or combination of methods (health insurance records, cancer and pathology registries, self-report, next-of-kin) | 11 | prospective |
| Catov 2011/<br>Denmark/<br>Danish National Birth Cohort | Female population/<br>pregnant (~11 weeks of gestation)/<br>partly smoking during pregnancy (16%) | ≤25 13%<br>26-30 42%<br>31-35 32%<br>≥36 13%<br><br>♀ 100% | Multivitamins supplementation/<br>periconceptional (4 weeks before through 14 weeks after the last menstrual period)/<br>tabular format | Use vs. Non-Use | Preterm birth/<br>delivery <37 week of gestation<br><br>gestational age via early ultrasound in >90% of cases, otherwise self-report of last menstrual period | 5-24 weeks of gestation till delivery | prospective |
| Chiuve 2012/<br>US/<br>HPFS | Male population/<br>health professionals/<br>without CVD, diabetes or cancer | 40-75/<br>mean: 53<br><br>♀ 0% | Healthy diet/<br>validated FFQ/ at baseline, updated every 4 years/<br>HEI-2005 | High vs. Low/<br>Highest vs. Lowest quintile | Type 2 diabetes/<br><br>self-report/ confirmed by validated supplementary questionnaire | 22 | prospective |
| Chiuve 2012/<br>US/<br>NHS | Female population/<br>nurses/<br>without CVD, diabetes or cancer | 38-63/<br>mean: 50<br><br>♀ 100% | Healthy diet/<br>validated FFQ/ at baseline, updated every 2-4 years/<br>HEI-2005 | High vs. Low/<br>Highest vs. Lowest quintile | Type 2 diabetes/<br><br>self-report/ confirmed by validated supplementary questionnaire | 24 | prospective |

| | | | | | | | |
|---|---|---|---|---|---|---|---|
| Cohen 2008/<br>US/<br>NHANES III | Male and female population/<br>general population/ non-institutionalised/<br>without history of CVD/ | ≥30/<br>mean: 48<br><br>♀ 55 | Dietary sodium intake/<br>24h dietary recall/ interview/<br>at baseline | Dose-response:<br>per 1,000mg/day<br>(43.48mmol) | All-cause mortality/<br><br>National Death Index | 8.7 | prospective |
| | | | | | Cardiovascular disease mortality/<br><br>National Death Index | | |
| Cui 2008/<br>US/<br>WHI-OS | Female population/<br>postmenopausal/ without history of breast cancer/ without medical condition predictive of a survival time of <3 years | 50-79/<br>mean: 63.5<br><br>♀ 100 | Vitamin C supplementation/<br>standardised record/ at baseline | Dose-response:<br>per 500mg/day | Invasive breast cancer/<br><br>self-report/ confirmed by medical record or pathology report | 7.6 | prospective |
| Curhan 1997/<br>US/<br>NHS | Female population/<br>nurses/<br>without history of kidney stones | 34-59/<br>mean: 52.7<br><br>♀ 100 | Calcium supplementation/<br>validated FFQ/ at baseline, updated every 2-4 years | Dose-response:<br>per 1,600mg/day | Kidney stone/<br><br>self-report/ confirmed by supplementary questionnaire, medical records | 12 | prospective |
| Czeizel 2004/<br>Hungary/<br>Cohort-Controlled Trial of the Hungarian Periconceptional Service (HPS) | Female population/<br>supplemented cohort: participants of the HPS, planning to get pregnant/<br>unsupplemented cohort:<br>pregnant (8-12 weeks of gestation), not using multivitamins or folic acid during the periconceptional period | ≤19 1%<br>20-29 74%<br>30-39 24%<br>≥40 1%/<br>mean: 27.4<br><br>♀ 100% | Folic acid-containing multivitamin supplementation/<br>supplemented cohort: 0.8mg/day, with verified compliance/<br>unsupplemented cohort:<br>questionnaire by interview | Use vs. Non-Use | Cardiovascular congenital abnormalities/<br>ventricular septal defect, other conotruncal defect and atrial septal defect type II/<br><br>Medical records, autopsy reports | pre-conceptional or week 8-12 till delivery or fatal death | cohort-controlled trial |
| Dong 2008/<br>US/<br>The Seattle Barrett's Esophagus Program | Male and female population/ with histologically confirmed Barrett's oesophagus/ without history of oesophageal cancer | 30-54 30%<br>55-69 43%<br>≥70 27%<br><br>♀ 19% | Selenium supplementation/<br>validated questionnaire on supplement use/ at baseline and subsequent follow-up exams | Dose-response:<br>per 200µg/day | Oesophageal adenocarcinoma<br><br>endoscopy, biopsy | 5 | prospective |
| Egnell 2017/<br>France/<br>NutriNet-Santé cohort | Male and female population/<br>general population | ≥45/<br>mean: 57.1<br><br>♀ NR | Vitamin C supplementation/<br>web-based questionnaire/<br>at baseline | Dose-response:<br>per 500mg/day | Colon or rectum cancer/<br><br>self-report/ confirmed by medical records, physicians contact | 7 | prospective |
| Ferraro 2017/<br>US/<br>HPFS | Male population/<br>health professionals/<br>without history of kidney stones or malignancy (except non-melanoma skin cancer) | 40-75/<br><br>♀ 0% | Vitamin D supplementation/<br>validated FFQ/ at baseline, updated every 4 years | Dose-response:<br>per 1,000IU/day | Kidney stones/<br><br>self-report/ confirmed by medical records | ≤26 | prospective |

| | | | | | | | |
|---|---|---|---|---|---|---|---|
| Ferraro 2017/<br>US/<br>NHS | Female population/<br>nurses/<br>without history of kidney stones or malignancy (except non-melanoma skin cancer) | 30-55/<br><br>♀ 100% | Vitamin D supplementation/<br>validated FFQ/ at baseline, updated every 4 years | Dose-response:<br>per 1,000IU/day | Kidney stones/<br><br>self-report/ confirmed by medical records | ≤26 | prospective |
| Ferraro 2017/<br>US/<br>NHS II | Female population/<br>nurses/<br>without history of kidney stones or malignancy/ partly with history of diabetes or hypertension | 25-42/<br><br>♀ 100% | Vitamin D supplementation/<br>validated FFQ/ at baseline, updated every 4 years | Dose-response:<br>per 1,000IU/day | Kidney stones/<br><br>self-report/ confirmed by medical records | ≤20 | prospective |
| Gresham 2016/<br>Australia/<br>ALSWH | Female population/<br>general population/<br>preconceptional or pregnant in survey 3 or 5 | 20-25 or<br>31-36<br><br>♀ 100% | Healthy Diet/<br>validated FFQ/<br>at baseline, updated every 3-4 years/ Australian Recommended Food Score/ | High vs. Low/<br>40-63 vs. 4-24 points | Low birthweight/<br><2,500g<br><br>self-report | preconception till delivery/<br>3-9 years | prospective |
| Guasch-Ferre 2015/<br>Spain/<br>PREDIMED | Male and female population/<br>without CVD, but with type 2 diabetes mellitus or ≥3 cardiovascular risk factors | 55-80/<br>mean: 67<br><br>♀ 57% | PUFA intake/<br>validated FFQ/ interviews/<br>at baseline, updated yearly/<br>Spanish food-composition tables | High vs. Low/<br>9% of energy (median) vs. 4.2% of energy (median) | Cardiovascular events/<br><br>self-report, physicians report, medical records, National Death Index | 6 (median) | prospective (based on trial data) |
| Hansen 2013/<br>Denmark/<br>Diet, Cancer and Health Study | Male and female population/<br>general population/<br>without diagnosis of cancer (registered in the Danish Cancer Registry) | 50-64/<br>median: 56<br><br>♀ 59% | Selenium supplementation/<br>validated FFQ/<br>at baseline | Dose-response/<br>per 200µg/day | Colorectal cancer<br><br>register entry (Danish cancer registry, Danish Pathology Database) | 13 | prospective |
| Haugen 2009/<br>Norway/<br>Norwegian Mother and Child Cohort Study | Female population/<br>pregnant (~15 weeks of gestation)/<br>nulliparous | <20 4%<br>20-29 58%<br>30-39 37%<br>≥40 1%/<br><br>♀ 100% | Vitamin D supplementation/<br>validated FFQ/<br>at baseline | Dose-response/<br>per 2,000IU/day | Pre-eclampsia/<br>blood pressure >140/90 after 20 weeks' gestation, and proteinuria > +1 dipstick on at least 2 occasions<br><br>register entry (Medical Birth Registry of Norway) | ~15 weeks of gestation till delivery | prospective |
| Hillesund 2014/<br>Norway/<br>Norwegian Mother and Child Cohort Study | Female population/<br>pregnant (~22 weeks of gestation)/<br>with singleton pregnancy | mean: 30.1<br><br>♀ 100% | New Nordic Diet/<br>validated FFQ/ at baseline/<br>New Nordic Diet Score | High vs. Low/<br>6-10 vs. 0-3 points | Preterm birth/<br>delivery between 22-37 weeks of gestation<br><br>register entry (Medical Birth Registry of Norway) | ~22 weeks of gestation till delivery | prospective |

| | | | | | | | |
|---|---|---|---|---|---|---|---|
| InterAct 2011/<br>various European countries/<br>EPIC | Male and female population/<br>apparently healthy/ without diabetes | 25-70/<br>mean: 52.4<br><br>♀ 62% | Mediterranean diet/<br>validated FFQ or dietary history questionnaire/ country-specific/ at baseline/ rMED score | High vs. Low/<br>11-18 vs. 0-6 points | Type 2 diabetes mellitus/<br><br>self-report, linkage to care, drug, diabetes and pharmaceutical registers, hospital admissions, mortality data (at least two sources) | <15 | case-cohort |
| | | | Olive oil intake/<br>validated FFQ or dietary history questionnaire/ country-specific/ at baseline/ | Dose-response: per 10g/daily increase in olive oil | | | |
| Kirsh 2006/<br>US/<br>PLCO Trial (screening arm) | Male participants/<br>without history of cancer (except non-melanoma skin cancer) | 55-74/<br>mean: 63.3<br><br>♀ 0% | Vitamin C supplementation/<br>FFQ/ at baseline | Dose-response:<br>per 500mg/day | Prostate cancer incidence/<br><br>self-report/ confirmed by medical or pathology records and death certificates/ screening (serum PSA testing, digital rectal examination) | 4.2 (mean)/<br>8 (max) | prospective (based on trial data) |
| | | | ß-carotene supplementation/<br>FFQ/ at baseline | Dose-response:<br>per 20mg/day | | | |
| Lassale 2016/<br>various European countries/<br>EPIC | Male and female population/<br>without CVD, diabetes or previous cancer | 25-70/<br>mean: 50.8<br><br>♀ 71% | Mediterranean diet/<br>validated FFQ or diet history questionnaires/ country-specific/ interview/<br>MED score (0-9 points) | High vs. Low/<br>Highest vs. Lowest Quartile | Cancer mortality/<br><br>cancer registries or combination of methods (health insurance records, cancer and pathology registries, next-of-kin) | 10 | prospective |
| Lawson 2007/<br>US/<br>NIH-AARP Diet and Health Study | Male population/<br>without cancer (except non-melanoma skin cancer) | 50-71/<br>mean: 62.2<br><br>♀ 0% | Multivitamins supplementation/<br>FFQ (not validated)/ at baseline | High vs. Low/<br>≥7 times per week vs. never | Prostate cancer/<br><br>probabilistic linkage to eight state cancer registries | 5 | prospective |
| Leosdottir 2005_women/<br>Sweden/<br>MDC | Female population/<br>general population | mean: 57.5<br><br>♀ 100% | Saturated fat intake/<br>validated dietary questionnaire and 7-day menu diary | Low vs. High/<br>12.2% vs. 21.8% of energy intake | All-cause mortality/<br><br>register entry (local and national registries) | 6.6 | prospective |
| | | | | | Cardiovascular disease mortality/<br><br>register entry (local and national registries) | | |
| Liu 2003/<br>US/<br>NHS | Female population/<br>nurses/<br>without CVD, diabetes, or cancer | 38-63/<br><br>♀ 100% | Whole grains intake/<br>validated FFQ/ at baseline, updated every 2-4 years | High vs. Low/<br>0.9 servings/1,000kcal/day (median) vs. -5.9 servings/1,000kcal/day (median) | Body weight change/<br><br>Self-report | 12 | prospective |

| | | | | | | | |
|---|---|---|---|---|---|---|---|
| Ludwig 2001/<br>US/<br>Data obtained as part of the Planet Health intervention and evaluation project | Male and female population/<br>public school children (class 6 or 7)/<br>ethnically diverse | 11-12/<br>mean: 11.7<br><br>♀ 48% | Sugar-sweetened drink intake/<br>validated youth FFQ/ at baseline | Dose-response:<br>per increase in one daily serving | Change in BMI/<br><br>direct measurement/ stadiometer and portable electronic scale | 1.6 | prospective |
| Maruti 2009/<br>US/<br>VITAL | Female population/<br>postmenopausal/<br>without history of breast cancer | 50-76/<br>mean: 62<br><br>♀ 100% | Folic acid supplementation/<br>validated questionnaire (VITAL supplement questionnaire)/<br>at baseline | Dose-response:<br>per 2.5mg/day | Breast cancer/<br><br>register entry (Seattle–Puget Sound SEER registry) | 5 | prospective |
| Michels 2005/<br>US/<br>HPFS | Male population/<br>health professionals/<br>without cancer (except non-melanoma skin cancer) | 40-75/<br><br><br>♀ 0% | Dietary fibre intake/<br>validated FFQ/ at baseline, updated every 4 years/<br>AOAC method | High vs. Low/<br>>14.0g/1,000cal vs. <8.0g/1,000cal | Colorectal cancer/<br><br>self-report, next of kin/ confirmed by medical records, pathology reports/ non-respondents: National Death Index, postal service | 14 | prospective |
| Michels 2005/<br>US/<br>NHS | Female population/<br>nurses/<br>without cancer (except non-melanoma skin cancer) | 38-63/<br><br>♀ 100% | Dietary fibre intake/<br>validated FFQ/ at baseline, updated every 2-4 years/<br>AOAC method | High vs. Low/<br>>14.0g/1,000cal vs. <8.0g/1,000cal | Colorectal cancer/<br><br>self-report, next of kin/ confirmed by medical records, pathology reports/ non-respondents: National Death Index, postal service | 16 | prospective |
| Milton 2006/<br>US/<br>AREDS cohort | Male and female population/<br>with at least one natural lens/ without severe or last-stage lens opacities/ | <65 23%<br>65-69 34%<br>≥70 43%/<br><br>♀ 56% | Multivitamins/Minerals supplementation/<br>continue usage after trial | Use vs. Non-Use | Cortical opacity/<br><br>film slit-lamp camera, retro-illumination camera lens photographs | 6.3 (median) | prospective (based on trial data) |
| | | | | | Nuclear opacity/<br><br>film slit-lamp camera, retro-illumination camera lens photographs | | |
| | | | | | Posterior subcapsular opacity/<br><br>film slit-lamp camera, retro-illumination camera lens photographs | | |

| | | | | | | | |
|---|---|---|---|---|---|---|---|
| Milunsky 1989/<br>US | Female population/<br>pregnant (~16 weeks of gestation)/<br>undergoing maternal serum a-fetoprotein screening or amniocentesis | <20 2%<br>20-29 44%<br>30-39 52%<br>≥40 2%/<br><br>♀ 100% | Folic acid-containing multivitamins supplementation/<br>interview/ at baseline | Use vs. Non-Use | Neural-tube defects/<br>spina bifida, anencephaly, encephalocele alone or in combination with other defects<br><br>physician report or self-report | ~16 weeks of gestation till delivery or fatal death | prospective |
| Nohr 2014/<br>Denmark/<br>Danish National Birth Cohort | Female population/<br>pregnant (~11 weeks of gestation)/<br>partly smoking during pregnancy (16%) | <25 13%<br>25-29 42%<br>31-34 33%<br>≥35 12%/<br><br>♀ 100% | Multivitamins supplementation/<br>periconceptional (4 weeks before through 14 weeks after the last menstrual period)/<br>tabular format | Use vs. Non-Use | Late fetal death/<br>a non-deliberate demise of an intrauterine pregnancy (≥20 completed weeks of gestation)/<br><br>register entry (Civil Registration Register, National Birth Register, National Hospital Discharge Register) | ~11 weeks of gestation till delivery or fatal death | prospective |
| Pan 2013/<br>US/<br>NHS | Female population/<br>nurses/<br>without CVD, diabetes or cancer | 52-77/<br><br>♀ 100% | Nuts intake/<br>including walnuts, peanuts and other tree nuts/<br>validated FFQ/ at baseline, updated every 2-4 years | Dose-response:<br>per 4 servings (28.4g) per week | Type 2 diabetes mellitus/<br>American Diabetes Association criteria 1997<br><br>Self-report/ confirmed by supplementary questionnaire | 10 | prospective |
| Pan 2013/<br>US/<br>NHS II | Female population/<br>nurses/<br>without CVD, diabetes or cancer | 35-52/<br><br>♀ 100% | Nuts intake/<br>including walnuts, peanuts and other tree nuts/<br>validated FFQ/ at baseline, updated every 4 years | Dose-response:<br>per 4 servings (28.4g) per week | Type 2 diabetes mellitus/<br>American Diabetes Association criteria 1997<br><br>Self-report/ confirmed by supplementary questionnaire | 10 | prospective |
| Peters 2008/<br>US/<br>VITAL | Male population/<br>without prostate cancer | 50-76/<br><br>♀ 0% | Selenium supplementation/<br>validated questionnaire (VITAL supplement questionnaire)/<br>at baseline | Dose-response:<br>per 200µg/d | Prostate cancer/<br><br>register entry (Seattle–Puget Sound SEER registry) | 2-4 | prospective |
| Prentice 2013/<br>US/<br>WHI-OS | Female population/<br>postmenopausal/ without history of breast cancer/ without reported urinary tract stones | 50-79/<br><br>♀ 100% | Calcium and Vitamin D supplementation/<br>record of nutrients from participants' vitamin bottles, standardised interview/ at baseline and 3-year clinic visit | Use vs. Non-Use | Fractures/<br><br>Self-report/ confirmed by medical records | 7.2 | prospective |

| | | | | | | | |
|---|---|---|---|---|---|---|---|
| Rautiainen 2016/<br>US/<br>PHS | Male population/<br>physicians/<br>without CVD and cancer | 40-84/<br>mean: 52.9<br><br>♀ 0% | Multivitamin supplementation/<br>questionnaire on supplement use/ at baseline | Use vs. Non-Use | Myocardial infarction/<br><br>self-report/ confirmed by endpoint committee of physicians | 12.2 | prospective |
| | | | | | Stroke/<br><br>self-report/ confirmed by endpoint committee of physicians | | |
| Robien 2007/<br>US/<br>IWHS | Female population/<br>postmenopausal/<br>without cancer (except non-melanoma skin cancer) | 55-69/<br>mean: 61.5<br><br>♀ 100% | Vitamin D supplementation/<br>validated FFQ/ at baseline | Dose-response:<br>per 400IU/day | Breast cancer<br><br>registry entry (State Health Registry of Iowa, part of the SEER) | 18 | prospective |
| Rodriguez 2004/<br>US/<br>CPS-II Nutrition Cohort | Male population/<br>without cancer (except non-melanoma skin cancer) | 50-74/<br><60 25%<br>60-69 58%<br>70-79 16%<br>≥80 1%/<br><br>♀ 0% | Vitamin E supplementation/<br>FFQ (modification of the brief HHHQ)/ at baseline | Dose-response:<br>per 400IU/day | Prostate cancer incidence/<br><br>self-report/ confirmed by medical records, state cancer registries, death certificate, National Death Index | 7-8 | prospective |
| Schulze 2004/<br>US/<br>NHS II | Female population/<br>nurses/<br>without CVD, diabetes or cancer (except non-melanoma skin cancer) | 24-44/<br><br>♀ 100% | Sugar-sweetened soft drink intake/<br>validated FFQ/ at baseline, updated every 4 years | Low vs. High/<br>Reduced intake vs. Constant high | Mean weight change/<br><br>self-report | 8 | prospective |
| Skinner 2004/<br>US/<br>NHS | Female population/<br>nurses/ without cancer (except non-melanoma skin cancer) | 38-58/<br>mean: 50.8<br><br>♀ 100% | Folic acid supplementation/<br>validated FFQ/<br>at baseline, updated every 2-4 years | Dose-response:<br>per 2.5mg/day | Pancreatic cancer/<br><br>self-report/ confirmed by medical records, pathology records, next of kin, National Death Index, postal service | 16 | prospective |
| Slatore 2008/<br>US/<br>VITAL | Male and female population/<br>without history lung cancer/<br>partly history of prior cancer (14%), COPD (4%) or asthma (10%) | 50-76/<br>mean: 61.9<br><br>♀ 52% | Folic acid supplementation/<br>validated questionnaire (VITAL supplement questionnaire)/<br>at baseline | Dose-response:<br>per 2mg/day | Lung cancer/<br><br>register entry (Seattle–Puget Sound SEER registry) | 4.1 | prospective |
| | | | Vitamin C supplementation/<br>validated questionnaire (VITAL supplement questionnaire)/<br>at baseline | Dose-response:<br>per 500mg/day | | | |

| | | | | | | | |
|---|---|---|---|---|---|---|---|
| Timmermans 2011/<br>the Netherlands/<br>Generation R Study | Female population/<br>pregnant (~12 weeks of gestation)/<br>with singleton pregnancy/<br>without chronic hypertension, diabetes mellitus, hypercholesterolemia, chronic heart disorders, systemic lupus erythematosus | mean: 29.9/<br><br>♀ 100% | Folic acid supplementation/<br>questionnaire/ at baseline | Use vs. Non-use | Gestational hypertension/<br>blood pressure ≥140/90mmHg after 20 weeks of gestation<br><br>automated digital oscillometric sphygmanometer/ medical records | ~12 weeks of gestation till delivery | prospective |
| Timmermans 2012/<br>Denmark/<br>Generation R Study | Female population/<br>pregnant (~13.5 weeks of gestation)/<br>with live-born singleton pregnancy | mean: 31.3/<br><br>♀ 100% | Mediterranean diet/<br>(modified version of a) validated FFQ/ at baseline | High vs. Low/<br>Lowest vs. Highest tertile | Birth weight/<br><br>medical records | <18 weeks of gestation till delivery | prospective |
| Tobias 2012/<br>US/<br>NHS II | Female population/<br>nurses/<br>with singleton live birth/<br>without previous gestational diabetes/<br>without CVD, cancer or diabetes | 22-44/<br><br>♀ 100% | Mediterranean diet/<br>validated FFQ/ at baseline, updated every 4 years/<br>aMED score | High vs. Low/<br>6.6 points (mean) vs. 1.6 points (mean) | Gestational diabetes mellitus/<br><br>self-report of physician diagnosis | (10) | prospective |
| Tortosa 2007/<br>Spain/<br>SUN | Male and female population/<br>university graduates/<br>without metabolic syndrome, diabetes, hypertension, hypercholesterolemia, or hypertriglyceridemia, BMI <30 | NR<br><br>♀ NR | Mediterranean diet/<br>validated FFQ/ at baseline, updated every two years (not considered in analysis)/<br>MED-score (0-9 points) | High vs. Low/<br>6-9 vs. 0-2 points | Metabolic syndrome/<br>International Diabetes Federation criteria/<br><br>self-report | 6.2 (median) | prospective |
| | | | | | HDL-Cholesterol/<br><br>self-report | | |
| | | | | | Systolic blood pressure/<br><br>self-report | | |
| | | | | | Triglycerides/<br><br>self-report | | |
| Wang 2015/<br>US/<br>FHS offspring cohort | Male and female population/<br>without hypertension and not using anti-hypertensive medication | mean: 51.9<br><br>♀ 57.6 | Dairy intake/<br>validated FFQ/ at baseline, updated at 1-3 examination visits (not held at a regular interval) | High vs. Low/<br>≥3 vs. <1 servings/day | Systolic blood pressure/<br>mean annualised change in mmHg/<br><br>standardised examination/<br>random-zero sphygmomanometer | 14.6 (median) | prospective |

| | | | | | | | |
|---|---|---|---|---|---|---|---|
| Wang 2016/<br>China/<br>MABC | Female population/<br>pregnant/ with singleton pregnancy/<br>with live born infant/<br>without diabetes, chronic hypertension | mean: 26.4<br><br>♀ 100% | Folic acid + iron supplementation/<br>interview/ updated in every trimester of pregnancy | Use vs. Non-use<br>(in 2 and 3 trimester)/ use defined as taking >2 times/week | Low birthweight/<br><2,500g/<br><br>Hospital delivery logs<br><br>Birth weight/<br><br>Hospital delivery logs | first prenatal examination till delivery | prospective |
| Wen 2016/<br>Canada/<br>OaK birth cohort | Female population/<br>pregnant (<20 weeks of gestation) | <25 2%<br>25-29 40%<br>30-34 37%<br>≥35 21%/<br><br>♀ 100% | Folic acid supplementation/<br>interview | Use vs. Non-use | Pre-eclampsia/<br>gestational hypertension and proteinuria/<br><br>Self-report, medical charts reviewed by study investigators | <20 weeks of gestation till delivery | prospective |
| Yang 2016/<br>US/<br>CPS-II Nutrition Cohort | Female population/<br>without CVD or cancer/<br>partly overweight or obese (46%)/<br>partly with history of hypertension (31%), high cholesterol (54%), or diabetes (5%) | 50-74 (whole sample)/<br><60 38%<br>60-64 27%<br>65-69 21%<br>≥70 14%/<br><br>♀ 100% | Calcium supplementation/<br>validated FFQ/ at baseline, updated after 6 and 10 years | Dose-response:<br>per 1,000mg/day | Cardiovascular disease mortality/<br><br>National Death Index | 17.5 | prospective |
| Yu 2014/<br>China/<br>SMHS | Male population/<br>general population/<br>without cancer | 40-74/<br><br>♀ 0% | Healthy diet/<br>validated FFQ/ at baseline/<br>mAHEI index | High vs. Low/<br>Highest vs. Lowest quartile | All-cause mortality/<br><br>register entry (Shanghai Cancer registry and Shanghai Vital Statistics Registry), home visits | 6.5 | prospective |
| Yu 2014/<br>China/<br>SWHS | Female population/<br>general population/<br>without cancer | 40-70/<br><br>♀ 100% | Healthy diet/<br>validated FFQ/ at baseline/<br>mAHEI index | High vs. Low/<br>Highest vs. Lowest quartile | All-cause mortality/<br><br>register entry (Shanghai Cancer registry and Shanghai Vital Statistics Registry), home visits | 12 | prospective |
| Zschäbitz 2013/<br>US/<br>WHI-OS | Female population/<br>postmenopausal/ without medical condition predictive of a survival time of <3 years | 50-79/<br>mean: 63.5<br><br>♀ 100% | Folic acid supplementation/<br>inventory-type questionnaire/ standardised interview, record of nutrients from participants' vitamin bottles/ at baseline | Dose-response:<br>per 2.5mg/day | Colorectal cancer incidence/<br><br>self-report, review of medical records and clinic follow-up visit | 11 (median) | prospective |

ALSWH: Australian Longitudinal Study on Women's Health; AOAC: Association of Official Analytical Chemists; AREDS: Age-Related Eye Disease Study; arMED: Alternative/adapted relative Mediterranean diet score; BMI: body mass index; COPD: chronic obstructive pulmonary disease; CPS-II: Cancer Prevention Study II; CVD: cardiovascular disease; EPIC: European

Prospective Investigation into Cancer and Nutrition; FFQ: food-frequency questionnaire; FHS: Framingham Heart Study; HDL: high-density lipoprotein; HEI-2005: Healthy Eating Index-2005; HHHQ: Health Habits and History Questionnaire; HPFS: Health Professionals Follow-up Study; HPS: Hungarian Periconceptional Service; IWHS: Iowa Women's Health Study; MABC: Ma'anshan-Anhui Birth Cohort Study; mAHEI: modified Alternative Healthy Eating Index; MDC: Malmö Diet and Cancer Study; MED: Mediterranean diet; NHANES III: Third National Health and Nutrition Examination Survey; NHS: Nurses' Health Study; NHS II: Nurses' Health Study II; NIH-AARP: National Institutes of Health and formerly the American Association of Retired Persons; NR: not reported; OaK: The Ottawa and Kingston birth cohort; PHS: Physicians' Health Study cohort; PLCO: Prostate, Lung, Colorectal, and Ovarian Cancer Screening Trial; PREDIMED: Prevención con Dieta Mediterránea; PSA: prostate-specific antigen; PUFA: polyunsaturated fatty acid; rMED: relative Mediterranean diet score; SEER: Surveillance, Epidemiology, and End Results; SMHS: Shanghai Men's Health Study; SUN: Seguimiento Universidad de Navarra dynamic cohort; SWHS: Shanghai Women's Health Study; US: United States of America; VITAL: VITamins And Lifestyle cohort; WHI-OS: Women's Health Initiative Observational Study cohort

**Table S5** Description of study design pairs

| | | | Randomised controlled trials | | | | Cohort studies | | | |
|---|---|---|---|---|---|---|---|---|---|---|
| **Reference (Systematic review)** | **Intervention/ Exposure** | **Outcome** | **Reference** | **Participants/ Cases** | **Comparison/ Dose** | **Summary measure and effect estimate (95% CI)** | **Reference** | **Participants/ Cases** | **Comparison/ Dose** | **Summary measure and effect estimate (95% CI)** |
| Abdelhamid 2018b/ Zhu 2019 | PUFA | Major adverse cardiac and cerebrovascular events | Burr 1989 | 2,033/ 670 | High vs. Low | RR 0.99 (0.87 to 1.12) | Guasch-Ferre 2016 | 7,038/ 336 | High vs. Low | HR 0.68 (0.48 to 0.96) |
| Aburto 2013 | Low-sodium | All-cause mortality | TOHP II 1997 | 2,382/ 16 | Low vs. High | RR 0.67 (0.36 to 1.25) | Cohen 2008 | 8,699/ 1,150 | Dose-response/ per 1,000mg/day increase | HR 1.06 (0.99 to 1.14) |
| Aburto 2013 | Low-sodium | Cardiovascular disease | Whelton 1998 | 663/ 82 | Low vs. High | RR 0.78 (0.52 to 1.18) | Cohen 2008 | 8,669/ 436 | Dose-response/ per 1,000mg/day increase | HR 1.14 (0.99 to 1.30) |
| Afshin 2014 | Nuts | Coronary Heart Disease | Estruch 2018 | 7,447/ 69 | Dose-response/ per 4 servings (28.4g) | RR 0.79 (0.55 to 1.14) | Bao 2013_NHS | 76,464/ 2,208 | Dose-response/ per 4 servings (28.4g) | RR 0.80 (0.69 to 0.93) |
| | | | | | | | Bao 2013_HPFS | 42,498/ 2,698 | Dose-response/ per 4 servings (28.4g) | RR 0.82 (0.74 to 0.90) |
| Afshin 2014 | Nuts | Stroke | Estruch 2018 | 7,447/ 90 | Dose-response/ per 4 servings (28.4g) | RR 0.62 (0.44 to 0.87) | Bernstein 2012_NHS | 84,010/ 2,633 | Dose-response/ per 4 servings (28.4g) | RR 0.81 (0.66 to 1.00) |
| | | | | | | | Bernstein 2012_HPFS | 43,150/ 1,397 | Dose-response/ per 4 servings (28.4g) | RR 0.95 (0.82 to 1.11) |
| Afshin 2014 | Nuts | Type 2 Diabetes | Salas-Salvadó 2014 | 3,541/ 273 | Dose-response/ per 4 servings (28.4g) | RR 0.86 (0.68 to 1.08) | Pan 2013_NHS | 58,063/ 5,121 | Dose-response/ per 4 servings (28.4g) | RR 0.91 (0.84 to 0.98) |
| | | | | | | | Pan 2013_NHS II | 79,893/ 4,098 | Dose-response/ per 4 servings (28.4g) | RR 0.83 (0.75 to 0.92) |
| Bjelakovic 2014b/ Hossain 2019 | Vitamin D | Breast cancer | Brunner 2011 | 36,282/ 1,028 | High vs. Low/ 400IU/day | RR 0.96 (0.85 to 1.09) | Robien 2007 | 34,321/ 2,440 | Dose-response/ per 400IU/day | RR 0.96 (0.91 to 1.01) |
| Bolland 2015 | Calcium | All fractures | Jackson 2006 | 36,282/ 4,260 | High vs. Low/ 500mg/day | RR 0.97 (0.92 to 1.03) | Prentice 2013 | 39,037/ 5,516 | Use vs. Non-use | HR 1.07 (1.01 to 1.14) |
| Chung 2016 | Calcium | Cardiovascular disease mortality | Hsia 2007 | 36,282/ 258 | High vs. Low/ 1,000IU/day | RR 1.01 (0.79 to 1.29) | Yang 2016 | 73,079/ 5,712 | Dose-response/ per 1,000mg/day | RR 0.85 (0.78 to 0.92) |
| DeRegil 2015/ Blencowe 2010 | Folic acid | Neural tube defect | Czeizel 1994 | 4,862/ 6 | High vs. Low/ 800µg/day | RR 0.07 (0.00 to 1.32) | Milunsky 1989 | 22,657/ 49 | Use vs. Non-use | RR 0.29 (0.15 to 0.55) |

| | | | | | | | | | | |
|---|---|---|---|---|---|---|---|---|---|---|
| Ding 2017 | Dairy | Systolic blood pressure | Barr 2000 | 200/ NA | High vs. Low | MD 1.00 (-1.26 to 3.26)/ SMD 0.12 (-0.16 to 0.4) | Wang 2015 | 2,075/ NA | High vs. Low | MD -0.60 (-0.86 to -0.34)/ SMD -0.25 (-0.35 to -0.14) |
| Feng 2015 | Folic acid | Congenital heart defects | Czeizel 1998 | 4,862/ 30 | High vs. Low/ 0.8mg/day | RR 0.48 (0.23, 1.03) | Czeizel 2004 | 6,112/ 81 | Use vs. Non-use | OR 0.60 (0.38 to 0.96) |
| Fu 2021 | Vitamin C | Lung cancer | Lin 2009 | 7,627/ 48 | High vs. Low/ 500mg/day | RR 1.84 (1.14 to 2.97) | Slatore 2008 | 77,126/ 521 | Dose-response/ per 500mg/day | HR 0.95 (0.74 to 1.21) |
| Fu 2021 | Vitamin C | Breast Cancer | Lin 2009 | 7,627/ 257 | High vs. Low/ 500mg/day | RR 1.11 (0.87 to 1.41) | Cui 2008 | 84,805/ 2,879 | Dose-response/ per 500mg/d | RR 1.08 (1.02 to 1.14) |
| Fu 2021 | Vitamin C | Colorectal cancer | Lin 2009 | 7,627/ 44 | High vs. Low/ 500mg/day | RR 0.76 (0.42 to 1.38) | Egnell 2017 | 38,658/ 120 | Dose-response/ per 500mg/day | HR 0.00 (0.00 to 11.67) |
| Gayer 2019 | Apples | Body weight | Chai 2012 | 100/ NA | High vs. Low/ 75g/day | MD -2.00 (-5.79 to 1.79) | Bertoia 2015 | 35,408/ NA | Dose-response/ per serving/day | MD -0.53 (-0.59 to -0.47) |
| Grosso 2015 | Mediterranean diet | Cardiovascular disease | Estruch 2018 | 7,447/ 288 | High vs. Low | RR 0.81 (0.64 to 1.02) | Buckland 2009 | 40,757/ 606 | High vs. Low | HR 0.60 (0.47 to 0.77) |
| Hemmingsen 2017/ Schwingshackl 2018 | Healthy diet | All-cause mortality | Pan 1997 | 263/ 6 | High vs. Low | RR 1.02 (0.21 to 4.98) | Yu 2014 | 134,455/ 7,302 | High vs. Low | HR 0.74 (0.63 to 0.86) |
| Hemmingsen 2017/ Schwingshackl 2018 | Healthy diet | Type 2 Diabetes | Pan 1997 | 263/ 147 | High vs. Low | RR 0.65 (0.52 to 0.81) | Chiuve 2012 | 112,524/ 8,337 | High vs. Low | HR 0.82 (0.76 to 0.89) |
| Hooper 2015b/ de Souza 2015 | Low-fat diet | All-cause mortality | Howard 2006 | 48,835/ 2,404 | Low vs. High | RR 0.98 (0.90 to 1.06) | Leosdottir 2005_women | 17,035/ 522 | Low vs. High | RR 1.12 (0.81 to 1.56) |
| Hooper 2015b/ de Souza 2015 | Low-fat diet | Cardiovascular disease mortality | Howard 2006 | 46,558/ 428 | Low vs. High | RR 0.99 (0.81 to 1.20) | Leosdottir 2005_women | 17,035/ 57 | Low vs. High | RR 1.82 (0.85 to 3.85) |
| Hyppönen 2013 | Vitamin D | Pre-eclampsia | Hollis 2011 | 350/ 18 | High vs. Low/ 1,600IU/day or 3,600IU/day | RR 0.50 (0.20 to 1.23) | Haugen 2009 | 23,423/ 1,267 | Dose-response/ per 2,000IU/day | RR 0.66 (0.45 to 0.99) |
| Jonker 2020 | Folic acid | Birth weight | Christian 2003 | 1,313/ NA | High vs. Low/ 400g/day | MD 0.02 (-0.07 to 0.03)/ SMD 0.00 (-0.11 to 0.11) | Wang 2016 | 2,644/ NA | Use vs. Non-use | MD 0.08 (-0.15 to 0.00)/ SMD 0.18 (0.00 to 0.35) |
| Jonker 2020 | Folic acid | Low birthweight | Christian 2003 | 1,313/ 559 | High vs. Low/ 400g/day | RR 0.96 (0.85 to 1.09) | Wang 2016 | 2,644/ 51 | Use vs. Non-use | RR 0.35 (0.05 to 2.49) |
| Kastorini 2011 | Mediterranean diet | Metabolic syndrome | Salas-Salvadó 2008 | 1,224/ 695 | High vs. Low | RR 0.90 (0.82 to 1.00) | Tortosa 2007 | 2,563/ 56 | High vs. Low | RR 0.39 (0.14 to 0.81) |
| Kastorini 2011 | Mediterranean diet | HDL-Cholesterol | Esposito 2009 | 215/ NA | High vs. Low | MD 0.07 (0.02 to 0.14)/ SMD 1.19 (0.90 to 1.48) | Tortosa 2007 | 2,563/ NA | High vs. Low | MD 0.01 (-0.05 to 0.06)/ SMD 0.02 (-0.10 to 0.14) |

| | | | | | | | | | | |
|---|---|---|---|---|---|---|---|---|---|---|
| Kastorini 2011 | Mediterranean diet | Trigylcerides | Esposito 2009 | 215/<br>NA | High vs. Low | MD -0.21 (-0.36 to 0.02)/<br>SMD -0.99 (-1.28 to -0.71) | Tortosa 2007 | 2563/<br>NA | High vs. Low | MD -0.02 (-0.08 to 0.03)/<br>SMD -0.05 (-0.17 to 0.08) |
| Kastorini 2011 | Mediterranean diet | Systolic blood pressure | Esposito 2009 | 215/<br>NA | High vs. Low | MD -1.50 (-4.50 to 1.20)/<br>SMD -0.76 (-1.03 to -0.48) | Tortosa 2007 | 2563/<br>NA | High vs. Low | MD 0.80 (-0.84 to 2.44)/<br>SMD 0.06 (-0.06 to 0.18) |
| Kelly 2017/<br>Ye 2012 | Whole grain | Body weight | Maki 2010 | 173/<br>NA | High vs. Low/<br>two portions à 40g/day | MD -0.40 (-0.49 to -0.31)/<br>SMD -1.33 (-1.66 to -1.00) | Liu 2003 | 74,091/<br>NA | High vs. Low | MD -0.29 (-0.35 to 0.23)/<br>SMD -0.12 (-0.14 to -0.1) |
| Kim 2018 | Multivitamins/<br>Minerals | Cardiovascular disease mortality | Sesso 2012 | 14,641/<br>829 | High vs. Low | HR 0.95 (0.83 to 1.09) | Bailey 2015 | 8,599/<br>1,636 | High vs. Low | HR 0.65 (0.49 to 0.85) |
| Kim 2018 | Multivitamins/<br>Minerals | Coronary Heart Disease | Sesso 2012 | 14,641/<br>585 | High vs. Low | HR 0.93 (0.79 to 1.09) | Rautiainen 2016 | 18,530/<br>815 | Use vs. Non-use | HR 0.88 (0.74 to 1.04) |
| Kim 2018 | Multivitamins/<br>Minerals | Stroke | Sesso 2012 | 14,641/<br>546 | High vs. Low | HR 1.06 (0.89 to 1.25) | Rautiainen 2016 | 18,530/<br>670 | Use vs. Non-use | HR 0.94 (0.79 to 1.13) |
| Lin 2020 | Vitamin D | Nephrolithiasis | Baron 2015 | 2,259/<br>47 | High vs. Low/<br>1,000IU/day | RR 0.68 (0.38 to 1.21) | Ferraro 2017 | 193,551/<br>6,576 | Dose-response/<br>per 1,000IU/day | RR 1.10 (0.99 to 1.22) |
| Lin 2020 | Calcium | Nephrolithiasis | Riggs 1998 | 236/<br>1 | High vs. Low/<br>1,600mg/day | RR 0.33 (0.01 to 7.96) | Curhan 1997 | 91,731/<br>864 | Dose-response/<br>per 1,600IU/day | RR 1.59 (0.95 to 2.64) |
| Moazzen 2018 | Folic acid | Colorectal cancer | Zhang 2008 | 5,442/<br>40 | High vs. Low/<br>2.5mg/day | RR 0.82 (0.44 to 1.52) | Zschäbitz 2013 | 88,045/<br>1,003 | Dose-response/<br>per 2.5mg/day | HR 0.89 (0.25 to 3.20) |
| Morze 2021 | Mediterranean diet | Cancer mortality | de Lorgeril 1998 | 605/<br>7 | High vs. Low | RR 0.75 (0.17 to 3.33) | Lassale 2016 | 451,256/<br>7,475 | High vs. Low | HR 0.82 (0.77 to 0.88) |
| Morze 2021 | Mediterranean diet | Breast cancer | Toledo 2018 | 4,152/<br>35 | High vs. Low | RR 0.41 (0.19 to 0.86) | Buckland 2013 | 335,062/<br>10,225 | High vs. Low | HR 0.94 (0.88 to 1.00) |
| Rees 2019/<br>Soltani 2019 | Mediterranean diet | All-cause mortality | Estruch 2018 | 7,447/<br>348 | High vs. Low | RR 1.01 (0.81 to 1.25) | Alvarez-Alvarez 2017 | 19,467/<br>305 | High vs. Low | HR 0.79 (0.51 to 1.22) |
| Schwingshackl 2015 | Mediterranean diet | Type 2 Diabetes | Salas-Salvado 2014 | 3,541/<br>273 | High vs. Low | HR 0.69 (0.53 to 0.91) | InterAct 2011 | 15,798/<br>749 | High vs. Low | RR 0.88 (0.79 to 0.97) |
| Schwingshackl 2017 | Olive Oil | Type 2 Diabetes | Salas-Salvado 2014 | 2,301/<br>181 | High vs. Low/<br>10g/day | HR 0.60 (0.42 to 0.84) | InterAct 2011 men | 11,543/<br>NR | Dose-response/<br>per 10g/day | RR 0.89 (0.80 to 0.99) |
| | | | | | | | InterAct 2011 women | 15,500/<br>NR | Dose-response/<br>per 10g/day | RR 0.86 (0.77 to 0.97) |
| Stratton 2011 | Multivitamins | Prostate cancer | Meyer 2005 | 5,034/<br>103 | High vs. Low | RR 0.90 (0.62 to 1.33) | Lawson 2007 | 29,5344/<br>10,241 | High vs. Low | RR 1.03 (0.99 to 1.07) |
| Stratton 2011 | Vitamin E | Prostate cancer | Gaziano 2009 | 13,983/<br>1,008 | High vs. Low/<br>400IU on alternate days | RR 0.96 (0.85 to 1.08) | Rodriguez 2004 | 72,704/<br>4,281 | Dose-response/<br>per 400IU on alternate day | RR 1.00 (0.97 to 1.03) |

| | | | | | | | | | | |
|---|---|---|---|---|---|---|---|---|---|---|
| Stratton 2011 | Vitamin C | Prostate cancer | Gaziano 2009 | 13,983/<br>1,008 | High vs. Low/<br>500mg/day | RR 1.01 (0.90 to 1.14) | Kirsh 2006 | 29361/<br>1,338 | Dose-response/<br>per 500mg/day | RR 1.01 (0.94 to 1.09) |
| Stratton 2011 | β-carotene | Prostate cancer | Heinonen 1998 | 29,133/<br>246 | High vs. Low/<br>20mg/day | RR 1.24 (0.96 to 1.59) | Kirsh 2006 | 29,361/<br>1,338 | Dose-response/<br>per 20mg/day | RR 0.55 (0.10 to 2.92) |
| Te Morenga 2013 | Low dietary sugar | Body weight change | Reid 2007 | 133/<br>NA | Low vs. High | MD -1.37 (-2.35 to -0.39)/<br>SMD -0.47 (-0.81 to -0.12) | Schulze 2004 | 51603/<br>NA | Low vs. High | MD -1.55 (-2.00 to -1.10)/<br>SMD -0.25 (-0.32 to -0.17) |
| Te Morenga 2013 | Low dietary sugar | Body mass index | Sichieri 2009 | 927/<br>NA | High vs. Low | MD 0.10 (-0.06 to 0.10) | Ludwig 2001 | 548/<br>NA | Dose-response/<br>per one serving/day | MD 0.24 (0.10 to 0.39) |
| Tieu 2017/<br>Chia 2019 | Healthy diet | Preterm birth | Walsh 2012 | 759/<br>11 | High vs. Low | RR 0.39 (0.10 to 1.46) | Hillesund 2014 | 72,072/<br>2,127 | High vs. Low | RR 0.91 (0.80 to 1.03) |
| Tieu 2017/<br>Chia 2019 | Healthy diet | Small for gestational age | Moses 2014 | 576/<br>42 | High vs. Low | RR 0.86 (0.48 to 1.54) | Gresham 2016 | 1,907/<br>62 | High vs. Low | RR 0.42 (0.11 to 1.09) |
| Tieu 2017/<br>Chia 2019 | Healthy diet | Birth weight | Walsh 2012 | 759/<br>NA | High vs. Low | MD 0.03 (-0.05 to 0.10) | Timmermans 2012 | 3,207/<br>NA | High vs. Low | MD 0.07 (0.03 to 0.11) |
| Tieu 2017/<br>Mijatovic-Vukas 2018 | Mediterranean diet | Gestational diabetes | Walsh 2012 | 721/<br>30 | High vs. Low | RR 0.71 (0.35 to 1.45) | Tobias 2012 | 15,254/<br>872 | High vs. Low | RR 0.76 (0.60 to 0.95) |
| Vinceti 2018a | Selenium | Oesophageal cancer | Karp 2013 | 1,561/<br>1 | High vs. Low/<br>200µg/day | RR 1.50 (0.06 to 36.86) | Dong 2008 | 339/<br>37 | Dose-response/<br>per 200µg/d | HR 0.02 (0.00 to 1.94) |
| Vinceti 2018a | Selenium | Colorectal cancer | Lippman 2009 | 17,448/<br>123 | High vs. Low/<br>200µg/day | RR 1.04 (0.73 to 1.48) | Hansen 2013 | 54,208/<br>972 | Dose-response/<br>per 200µg/d | HR 0.52 (0.32 to 0.86) |
| Vinceti 2018a | Selenium | Prostate cancer | Lippman 2009 | 17,448/<br>848 | High vs. Low/<br>200µg/day | RR 1.03 (0.90 to 1.18) | Peters 2008 | 34,600/<br>818 | Dose-response/<br>per 200µg/d | HR 0.83 (0.37 to 1.89) |
| Wien 2012 | Folic acid | Lung cancer | Armitage 2010 | 12,064/<br>238 | High vs. Low/<br>2mg/day | RR 0.95 (0.74 to 1.22) | Slatore 2008 | 77,126/<br>521 | Dose-response/<br>per 2mg/day | HR 0.92 (0.40 to 2.11) |
| Wien 2012 | Folic acid | Pancreatic cancer | Zhang 2008 | 5,442/<br>10 | High vs. Low/<br>2.5mg/day | RR 1.50 (0.42 to 5.31) | Skinner 2004 | 77,640/<br>139 | Dose-response/<br>per 2,5mg/day | RR 5.86 (0.25 to 139,27) |
| Wien 2012 | Folic acid | Breast cancer | Zhang 2008 | 5,442/<br>154 | High vs. Low/<br>2.5mg/day | RR 0.83 (0.61 to 1.14) | Maruti 2009 | 35,023/<br>738 | Dose-response/<br>per 2,5mg/day | RR 0.91 (0.35 to 2.36) |
| Wolf 2017 | Multivitamins | Preterm birth | Brough 2010 | 353/<br>17 | High vs. Low | RR 1.09 (0.43 to 2.77) | Catov 2011 | 33,288/<br>1,617 | Use vs. Non-use | HR 0.89 (0.80 to 0.99) |
| Wolf 2017 | Multivitamins | Stillbirth | Kirke 1992 | 261/<br>2 | High vs. Low | RR 2.43 (0.12 to 50.05) | Nohr 2014 | 35,914/<br>206 | Use vs. Non-use | HR 0.82 (0.62 to 1.10) |
| Yang 2016 | Folic acid | Gestational hypertension | Merchant 2005 | 955/<br>89 | High vs. Low/<br>0.8mg/day | RR 0.63 (0.42 to 0.94) | Timmermans 2011 | 5,993/<br>301 | Use vs. Non-use | RR 1.21 (0.92 to 1.57) |
| Yao 2017/<br>Aune 2011 | Fibre | Colorectal cancer | Schatzkin 2000 | 1,905/<br>14 | High vs. Low | RR 2.47 (0.78 to 7.85) | Michels 2005_pooled | 124,226/<br>1,596 | High vs. Low | HR 0.94 (0.74 to 1.20) |

| | | | | | | | | | | |
|---|---|---|---|---|---|---|---|---|---|---|
| Yu 2021 | Folic acid | Pre-eclampsia | Charles 2005 | 2,819/ 66 | High vs. Low/ 200µg/day or 5mg/day | RR 0.61 (0.34 to 1.08) | Wen 2016 | 7,669/ 245 | Use vs. Non-use | RR 0.59 (0.34 to 1.02) |
| Zhao 2014 | Multivitamins/ Minerals | Nuclear cataract | Maraini 2008 | 1,020/ 202 | High vs. Low | HR 0.66 (0.50 to 0.88) | Milton 2006 | 4,590/ NR | Use vs. Non-use | RR 0.75 (0.61 to 0.91) |
| Zhao 2014 | Multivitamins/ Minerals | Cortical cataract | Maraini 2008 | 1,020/ 214 | High vs. Low | HR 0.78 (0.60 to 1.02) | Milton 2006 | 4,590/ NR | Use vs. Non-use | RR 0.91 (0.68 to 1.22) |
| Zhao 2014 | Multivitamins/ Minerals | Posterior subcapsular cataract | Maraini 2008 | 1,020/ 111 | High vs. Low | HR 2.00 (1.35 to 2.98) | Milton 2006 | 4,590/ NR | Use vs. Non-use | RR 1.02 (0.77 to 1.36) |

CI: confidence interval; HDL: high density lipoprotein; HPFS: Health Professionals Follow-up Study; HR: hazard ratio; MD: mean difference; NA: not applicable; NHS: Nurses' Health Study; NHS II: Nurses' Health Study II; OR: odds ratio; PUFA: polyunsaturated fatty acids; RR: risk ratio; SMD: standardised mean difference; TOHP II: Trials of Hypertension Prevention, Phase II

**Table S6** Population (P), Intervention/Exposure (I/E), Control (C), and Outcome (O) similarity

| BoE-pair | Intervention/ Exposure | Outcome | Population | Intervention/ Exposure and Control | Outcome | Overall |
|---|---|---|---|---|---|---|
| Armitage 2010/ Slatore 2008 | Folic acid | Lung cancer | 1 | 1 | 1 | 1 |
| Baron 2015/ Ferraro 2017 | Vitamin D | Nephrolithiasis | 2 | 1 | 1 | 2 |
| Barr 2000/ Wang 2015 | Dairy | Systolic blood pressure | 1 | 2 | 1 | 2 |
| Brough 2010/ Catov 2011 | Multivitamins | Preterm birth | 2 | 2 | 1 | 2 |
| Brunner 2011/ Robien 2007 | Vitamin D | Breast cancer | 1 | 1 | 1 | 1 |
| Burr 1989/ Guasch-Ferre 2015 | PUFA | Major adverse cardiac and cerebrovascular events | 2 | 1 | 1 | 2 |
| Chai 2012/ Bertoia 2015 | Apples | Body weight | 1 | 2 | 1 | 2 |
| Charles 2005/ Wen 2016 | Folic acid | Pre-eclampsia | 2 | 2 | 1 | 2 |
| Christian 2003/ Wang 2016 | Folic acid | Birth weight | 1 | 2 | 1 | 2 |
| Christian 2003/ Wang 2016 | Folic acid | Low birthweight | 1 | 2 | 1 | 2 |
| Czeizel 1994/ Milunsky 1989 | Folic acid | Neural tube defect | 1 | 2 | 1 | 2 |
| Czeizel 1998/ Czeizel 2004 | Folic acid | Congenital heart defects | 1 | 2 | 1 | 2 |
| de Lorgeril 1998/ Lassale 2016 | Mediterranean diet | Cancer mortality | 2 | 1 | 1 | 2 |
| Esposito 2009/ Tortosa 2007 | Mediterranean diet | HDL-Cholesterol | 2 | 1 | 1 | 2 |
| Esposito 2009/ Tortosa 2007 | Mediterranean diet | Trigylcerides | 2 | 1 | 1 | 2 |
| Esposito 2009/ Tortosa 2007 | Mediterranean diet | Systolic blood pressure | 2 | 1 | 1 | 2 |
| Estruch 2018/ Alvarez-Alvarez 2017 | Mediterranean diet | All-cause mortality | 2 | 1 | 1 | 2 |
| Estruch 2018/ Bao 2013 | Nuts | Coronary Heart Disease | 2 | 1 | 2 | 2 |
| Estruch 2018/ Bernstein 2012 | Nuts | Stroke | 2 | 1 | 1 | 2 |
| Estruch 2018/ Buckland 2009 | Mediterranean diet | Cardiovascular disease | 2 | 1 | 2 | 2 |
| Gaziano 2009/ Rodriguez 2004 | Vitamin E | Prostate cancer | 1 | 1 | 1 | 1 |
| Gaziano 2009/ Kirsh 2006 | Vitamin C | Prostate cancer | 1 | 1 | 1 | 1 |
| Heinonen 1998/ Kirsh 2006 | β-carotene | Prostate cancer | 2 | 1 | 1 | 2 |
| Hsia 2007/ Yang 2016 | Calcium | Cardiovascular disease mortality | 2 | 1 | 2 | 2 |
| Hollis 2011/ Haugen 2009 | Vitamin D | Pre-eclampsia | 1 | 1 | 2 | 2 |
| Howard 2006/ Leosdottir 2005 | Low-fat diet | All-cause mortality | 1 | 1 | 1 | 1 |

| | | | | | | |
|---|---|---|---|---|---|---|
| Howard 2006/ Leosdottir 2005 | Low-fat diet | Cardiovascular disease mortality | 1 | 1 | 1 | 1 |
| Jackson 2006/ Prentice 2013 | Calcium | All fractures | 1 | 2 | 1 | 2 |
| Karp 2013/ Dong 2008 | Selenium | Oesophageal cancer | 1 | 1 | 1 | 1 |
| Kirke 1992/ Nohr 2014 | Multivitamins | Stillbirth | 2 | 2 | 1 | 2 |
| Lin 2009/ Cui 2008 | Vitamin C | Breast Cancer | 2 | 1 | 1 | 2 |
| Lin 2009/ Egnell 2017 | Vitamin C | Colorectal cancer | 2 | 1 | 1 | 2 |
| Lin 2009/ Slatore 2008 | Vitamin C | Lung cancer | 1 | 1 | 1 | 1 |
| Lippman 2009/ Hansen 2013 | Selenium | Colorectal cancer | 1 | 1 | 1 | 1 |
| Lippman 2009/ Peters 2008 | Selenium | Prostate cancer | 1 | 1 | 1 | 1 |
| Maki 2010/ Liu 2003 | Whole grain | Body weight | 2 | 1 | 1 | 2 |
| Maraini 2008/ Milton 2006 | Multivitamins/ Minerals | Nuclear cataract | 3 | 2 | 1 | 3 |
| Maraini 2008/ Milton 2006 | Multivitamins/ Minerals | Cortical cataract | 3 | 2 | 1 | 3 |
| Maraini 2008/ Milton 2006 | Multivitamins/ Minerals | Posterior subcapsular cataract | 3 | 2 | 1 | 3 |
| Merchant 2005/ Timmermans 2011 | Folic acid | Gestational hypertension | 2 | 2 | 1 | 2 |
| Meyer 2005/ Lawson 2007 | Multivitamins | Prostate cancer | 1 | 2 | 1 | 2 |
| Moses 2014/ Gresham 2016 | Healthy diet | Small for gestational age | 1 | 2 | 1 | 2 |
| Pan 1997/ Chiuve 2012 | Healthy diet | Type 2 Diabetes | 2 | 1 | 1 | 2 |
| Pan 1997/ Yu 2014 | Healthy diet | All-cause mortality | 2 | 1 | 1 | 2 |
| Reid 2007/ Schulze 2004 | Low dietary sugar | Body weight change | 1 | 1 | 1 | 1 |
| Riggs 1998/ Curhan 1997 | Calcium | Nephrolithiasis | 1 | 1 | 1 | 1 |
| Salas-Salvadó 2008/ Tortosa 2007 | Mediterranean diet | Metabolic syndrome | 3 | 1 | 1 | 3 |
| Salas-Salvado 2014/ InterAct 2011 | Mediterranean diet | Type 2 Diabetes | 2 | 1 | 1 | 2 |
| Salas-Salvado 2014/ InterAct 2011 | Olive Oil | Type 2 Diabetes | 2 | 1 | 1 | 2 |
| Salas-Salvadó 2014/ Pan 2013 | Nuts | Type 2 Diabetes | 2 | 1 | 1 | 2 |
| Schatzkin 2000/ Michels 2005 | Fibre | Colorectal cancer | 3 | 1 | 1 | 3 |
| Sesso 2012/ Bailey 2015 | Multivitamins/ Minerals | Cardiovascular disease mortality | 1 | 2 | 1 | 2 |
| Sesso 2012/ Rautiainen 2016 | Multivitamins/ Minerals | Coronary Heart Disease | 1 | 2 | 1 | 2 |
| Sesso 2012/ Rautiainen 2016 | Multivitamins/ Minerals | Stroke | 1 | 2 | 1 | 2 |
| Sichieri 2009/ Ludwig 2001 | Low dietary sugar | Body mass index | 1 | 1 | 1 | 1 |
| TOHP II 1997/ Cohen 2008 | Low-sodium | All-cause mortality | 2 | 1 | 1 | 2 |

| | | | | | | |
|---|---|---|---|---|---|---|
| Toledo 2018/ Buckland 2013 | Mediterranean diet | Breast cancer | 2 | 1 | 1 | 2 |
| Walsh 2012/ Hillesund 2014 | Healthy diet | Preterm birth | 2 | 2 | 1 | 2 |
| Walsh 2012/ Timmermans 2012 | Healthy diet | Birth weight | 2 | 2 | 1 | 2 |
| Walsh 2012/ Tobias 2012 | Mediterranean diet | Gestational diabetes | 2 | 2 | 1 | 2 |
| Whelton 1998/ Cohen 2008 | Low-sodium | Cardiovascular disease | 2 | 1 | 2 | 2 |
| Zhang 2008/ Maruti 2009 | Folic acid | Breast cancer | 2 | 1 | 1 | 2 |
| Zhang 2008/ Skinner 2004 | Folic acid | Pancreatic cancer | 2 | 1 | 1 | 2 |
| Zhang 2008/ Zschäbitz 2013 | Folic acid | Colorectal cancer | 2 | 1 | 1 | 2 |
| | | **1 = more or less identical, N (%)** | **26 (40.6)** | **42 (65.6)** | **59 (92.2)** | **13 (20.3)** |
| | | **2 = similar but not identical, N (%)** | **33 (51.6)** | **22 (34.4)** | **5 (7.8)** | **46 (71.9)** |
| | | **3 = broadly similar, N (%)** | **5 (7.8%)** | **0 (0.0)** | **0 (0.0)** | **5 (7.8)** |

HDL: high density lipoprotein; PUFA: polyunsaturated fatty acids; TOHP II: Trials of Hypertension Prevention, Phase II

**Table S7** Overview of adjustments made in multivariable analysis in the included cohort studies

| Reference | Exposure | Outcome | Basis-Confounder | | | | | | | | Others | Comment |
|---|---|---|---|---|---|---|---|---|---|---|---|---|
| | | | Age | Sex | SES/ education | Smoking | Alcohol | Physical activity | Energy intake | Weight/ BMI | | |
| Alvarez-Alvarez 2017 | Mediterranean diet | All-cause mortality | X | X | X | X | X | T | X | X | year entering the cohort, family history of CVD, diabetes and hypertension at baseline, hypercholesterolemia, depression, following a special diet at baseline | |
| Bailey 2015 | Multivitamins/Minerals | Cardiovascular disease mortality | X | X | X | X | X | T | T | T | race/ ethnicity, hypertension or use of an antihypertension medication, high cholesterol or use of lipid-lowering medication, HDL cholesterol, diabetes mellitus, aspirin use in the previous 30 days; stratified by birth cohort | |
| Bao 2013/ HPFS | Nuts | Coronary heart disease | X | NA | | X | X | X | X | X | Caucasian, physical examination for screening purposes, multivitamin use, current aspirin use, family history of diabetes mellitus, family history of myocardial infarction, family history of cancer, history of diabetes mellitus, history of hypertension, history of hypercholesterolemia, intake of red/ processed meat, fruits, and vegetables | |
| Bao 2013/ NHS | Nuts | Coronary heart disease | X | NA | | X | X | X | X | X | Caucasian, physical examination for screening purposes, multivitamin use, current aspirin use, family history of diabetes mellitus, family history of myocardial infarction, family history of cancer, history of diabetes mellitus, history of hypertension, history of hypercholesterolemia, intake of red/ processed meat, fruits, and vegetables, menopausal status and hormone use | |
| Bernstein 2012/ HPFS | Nuts | Stroke | X | NA | | X | X | X | X | X | dietary protein sources, intakes of cereal fibre, fruit and vegetables, trans-fatty acids, parental history of early myocardial infarction, multivitamin use, vitamin E supplement use, aspirin use; stratified by time period | |
| Bernstein 2012/ NHS | Nuts | Stroke | X | NA | | X | X | X | X | X | dietary protein sources, intakes of cereal fibre, fruit and vegetables, trans-fatty acids, menopausal status and hormone replacement, parental history of early myocardial infarction, years of multivitamin use, vitamin E supplement use, aspirin use; stratified by time period | |
| Bertoia 2015/ NHS | Apples | Body weight | X | NA | | X | X | X | | X | hours of sitting or watching TV, hours of sleep, fried potatoes, juice, whole grains, refined grains, fried foods, nuts, whole-fat dairy, low-fat dairy, sugar sweetened beverages, sweets, processed meats, non-processed meats, trans fat, seafood | Energy intake was considered as mediating factor, and thus not included in the model. |

| | | | | | | | | | | | | |
|---|---|---|---|---|---|---|---|---|---|---|---|---|
| Bertoia 2015/ NHS II | Apples | Body weight | X | NA | | X | X | X | | X | hours of sitting or watching TV, hours of sleep, fried potatoes, juice, whole grains, refined grains, fried foods, nuts, whole-fat dairy, low-fat dairy, sugar sweetened beverages, sweets, processed meats, non-processed meats, trans fat, seafood | Energy intake was considered as mediating factor, and thus not included in the model. |
| Buckland 2009 | Mediterranean diet | Cardiovascular disease | X | X | X | X | NA | X | X | X | centre, presence of diabetes, hyperlipidaemia, hypertension | Alcohol consumption is part of the Mediterranean diet scoring method. |
| Buckland 2013 | Mediterranean diet | Breast Cancer | X | NA | X | X | X | X | X | X | centre, height, age at menarche, oral contraception use, breastfeeding, age at first full-term pregnancy, menopausal status, use of hormone replacement therapy, saturated fat intake | |
| Catov 2011 | Multivitamins | Preterm birth | X | NA | X | X | | | | X | parity | |
| Chiuve 2012/ HPFS | Healthy diet | Type 2 diabetes | X | NA | | X | NA | X | X | X | aspirin, vitamin E supplementation, family history of myocardial infarction, family history of colon cancer; history of hypercholesterolemia; history of hypertension | Alcohol consumption is part of the AHEI-2010 scoring method. |
| Chiuve 2012/ NHS | Healthy diet | Type 2 diabetes | X | NA | | X | NA | X | X | X | aspirin, vitamin E supplementation, family history of myocardial infarction, family history of colon cancer; history of hypercholesterolemia; history of hypertension | Alcohol consumption is part of the AHEI-2010 scoring method. |
| Cohen 2008 | Low-sodium | All-cause mortality | X | X | X | X | X | X | X | X | race, added table salt, history of diabetes, history of cancer, systolic blood pressure, cholesterol, dietary potassium, treatment for hypertension | |
| Cohen 2008 | Low-sodium | Cardiovascular disease | X | X | X | X | X | X | X | X | race, added table salt, history of diabetes, history of cancer, systolic blood pressure, cholesterol, dietary potassium, treatment for hypertension | |
| Cui 2008 | Vitamin C | Breast Cancer | X | NA | X | X | X | X | X | X | ethnicity, age at menarche, age at menopause, parity, age at first full-term pregnancy, oral contraceptive use, postmenopausal hormone use, dietary folate intake, hysterectomy, bilateral oophorectomy, history of benign breast disease, family history of breast cancer, dietary vitamin C intake | |
| Curhan 1997 | Calcium | Nephrolithiasis | X | NA | | | X | | | X | intake of supplemental calcium, dietary calcium, animal protein, potassium, sucrose, fluid | The variables considered in these models were selected on the basis of factors that published reports had indicated were related to formation of calcium stones |
| Czeizel 2004 | Folic acid | Congenital heart defects | | NA | | | | | | | Birth order, chronic maternal disorders, history of previous unsuccessful pregnancies: fetal and infant deaths or congenital abnormalities | Participants were matched by age, socioeconomic status, place of residence, and year of pregnancy. |
| Dong 2008 | Selenium | Oesophageal cancer | X | X | | X | | | | T | fruit and vegetable consumption, percent energy from fat, waist-to-hip ratio, nonsteroidal anti-inflammatory drug use | |

| | | | | | | | | | | | | |
|---|---|---|---|---|---|---|---|---|---|---|---|---|
| Egnell 2017 | Vitamin C | Colorectal cancer | X | X | X | X | X | X | X | X | height, numbers of dietary records, family history of cancer | |
| Ferraro 2017 | Vitamin D | Nephrolithiasis | X | NA | | | X | | | X | region of residence, history of diabetes, history of hypertension, use of thiazides, use of calcium supplements, intake of calcium, sodium, potassium, magnesium, animal protein, fructose, oxalate, vitamin C, caffeine, fluids, dietary vitamin D | Results were provided for HPFS, NHS and NHS II separately. |
| Gresham 2016 | Healthy diet | Small gestational age | X | NA | X | X | | X | | X | area of residence, parity | |
| Guasch-Ferre 2015 | PUFA | Major adverse cardiac and cerebrovascular events | X | X | X | X | X | X | X | X | intervention group, fibre, protein intake, dietary cholesterol, baseline diabetes, hypertension, hypercholesterolemia, family history of coronary heart disease, use of antihypertensive medication, use of oral antidiabetic agents, use of lipid-lowering drugs | |
| Hansen 2013 | Selenium | Colorectal cancer | X | X | X | X | X | X | | X | use of nonsteroidal anti-inflammatory drugs, intake of red and processed meat | |
| Haugen 2009 | Vitamin D | Pre-eclampsia | X | NA | X | X | | | | X | height, season of childbirth | |
| Hillesund 2014 | Healthy diet | Birth weight | X | NA | X | X | | X | X | X | parity, any diabetes, chronic hypertension | |
| InterAct 2011 | Mediterranean diet | Type 2 Diabetes | X | X | X | X | NA | X | X | X | stratified by centre | Alcohol consumption is part of the Mediterranean diet scoring method. |
| InterAct 2011 | Olive oil | Type 2 Diabetes | X | X | X | X | NA | X | X | X | stratified by centre, rMED components (mutually adjusted) | Alcohol consumption is part of the Mediterranean diet scoring method. |
| Kirsh 2006 | β-carotene | Prostate cancer | X | NA | | X | | X | X | X | race, study centre, family history of prostate cancer, total fat intake, red meat intake, diabetes, aspirin use, number of prostate cancer screening examinations during the follow-up period | |
| Kirsh 2006 | Vitamin C | Prostate cancer | X | NA | | X | | X | X | X | race, study centre, family history of prostate cancer, total fat intake, red meat intake, diabetes, aspirin use, number of prostate cancer screening examinations during the follow-up period | |
| Lassale 2016 | Mediterranean diet | Cancer mortality | X | X | X | X | NA | X | T | X | study centre, dietary score at baseline | |
| Lawson 2007 | Multivitamins | Prostate cancer | X | NA | X | X | X | X | X | X | height, family history of prostate cancer, race, marital status, personal history of diabetes, use of supplemental calcium, zinc, vitamin E, energy-adjusted daily dietary intakes of tomato products, fish, red meat, α-linolenic acid, calcium, vitamin D, zinc, tocopherol | |

| | | | | | | | | | | | | |
|---|---|---|---|---|---|---|---|---|---|---|---|---|
| Leosdottir 2005/ women | Low-fat | All-cause mortality | X | NA | X | X | X | X | X | X | marital status, fibre intake | |
| Leosdottir 2005/ women | Low-fat | Cardiovascular mortality | X | NA | X | X | X | X | X | X | marital status, fibre intake | |
| Ludwig 2001 | Low dietary sugar | Body mass index | X | X | | NA | NA | X | X | X | Triceps-skinfold thickness, ethnicity, schools, diet (percent energy from fat at baseline, energy adjusted fruit-juice intake at baseline, and change in these variables from baseline to follow-up); time spent watching television and videos, and change in time spent watching television and videos | |
| Maruti 2009 | Folic acid | Breast Cancer | X | NA | | | X | X | | X | race, family history of breast cancer, mammography within 2 y preceding baseline, history of breast biopsy, age at menarche, age at first birth, age at menopause, years of combined oestrogen and progestin postmenopausal hormone use, height | |
| Michels 2005/ HPFS | Fibre | Colorectal cancer | X | NA | | X | X | X | X | X | time period, family history of colorectal cancer, history of sigmoidoscopy or colonoscopy, height, regular aspirin use, duration of aspirin use, multivitamin supplement use, dietary folate, red meat consumption, processed meat, glycaemic load, calcium, methionine | |
| Michels 2005/ NHS | Fibre | Colorectal cancer | X | NA | | X | X | X | X | X | time period, family history of colorectal cancer, history of sigmoidoscopy or colonoscopy, height, regular aspirin use, duration of aspirin use, multivitamin supplement use, dietary folate, red meat consumption, processed meat, glycaemic load, calcium, methionine, menopausal status, postmenopausal hormone use | |
| Milton 2006 | Multivitamins/Minerals | Cortical cataract | X | X | X | X | | | | | race, type-specific lens status, age-related eye disease study treatment, anti-inflammatory drug use, run-in compliance, propensity score | |
| Milton 2006 | Multivitamins/Minerals | Nuclear cataract | X | X | X | X | | | | | race, type-specific lens status, age-related eye disease study treatment, anti-inflammatory drug use, run-in compliance, propensity score | |
| Milton 2006 | Multivitamins/Minerals | Posterior subcapsular cataract | X | X | X | X | | | | | race, type-specific lens status, age-related eye disease study treatment, anti-inflammatory drug use, run-in compliance, propensity score | |
| Milunsky 1989 | Folic acid | Neural-tube defect | | NA | | | | | | | / | unadjusted effect estimate |
| Nohr 2014 | Multivitamins | Stillbirth | | NA | | | | | | | / | unadjusted effect estimate |
| Pan 2013/ NHS | Nuts | Type 2 Diabetes | X | NA | | X | X | X | X | X | questionnaire-cycle, race, family history of diabetes, postmenopausal status, menopausal hormone use, use of multivitamin, other dietary variables (whole grains, fruits, vegetables, fish, red meat, coffee, and sugar-sweetened beverages) | |

| | | | | | | | | | | | | |
|---|---|---|---|---|---|---|---|---|---|---|---|---|
| Pan 2013/ NHS II | Nuts | Type 2 Diabetes | X | NA | | X | X | X | X | X | questionnaire-cycle, race, family history of diabetes, postmenopausal status, menopausal hormone use, use of multivitamin, other dietary variables (whole grains, fruits, vegetables, fish, red meat, coffee, and sugar-sweetened beverages) | |
| Peters 2013 | Selenium | Prostate cancer | X | NA | X | T | T | T | | T | family history of prostate cancer, benign prostatic hyperplasia, multivitamin use, stratified on PSA screening in the 2 years before baseline | |
| Prentice 2013 | Calcium and Vitamin D | All fractures | X | NA | | X | | | | X | non-white ethnicity, number of falls, family history of fracture, personal history of fracture, bisphosphate use | |
| Rautiainen 2016 | Multivitamins/M inerals | Coronary heart disease | X | NA | | X | X | X | | X | randomly assigned treatment (aspirin and ß-carotene), family history of myocardial infarction, diabetes history, hypertension history, hypercholesterolemia history, fruit and vegetable intake | |
| Rautiainen 2016 | Multivitamins/M inerals | Stroke | X | NA | | X | X | X | | X | randomly assigned treatment (aspirin and ß-carotene), family history of myocardial infarction, diabetes history, hypertension history, hypercholesterolemia history, fruit and vegetable intake | |
| Robien 2007 | Vitamin D | Breast cancer | X | NA | X | X | X | X | X | X | age at menarche, age at menopause, first degree relative with breast cancer, oestrogen use, age at first live birth, number of live births, live on a farm, mammogram history, daily fat intake | |
| Rodriguez 2004 | Vitamin E | Prostate cancer | X | NA | X | X | | | X | X | race, energy adjusted calcium, total fat, lycopene intake, family history of prostate cancer, PSA history | |
| Schulze 2004 | Low dietary sugar | Body weight change | X | NA | | X | X | X | | X | Cereal fibre intake, total fat intake, changes in confounders over time, baseline energy intake from non-soda sources and changes over time, baseline intake of red meat, French fries, processed meat, sweets, snacks, vegetables, and fruits, and changes over time, postmenopausal hormone use, oral contraceptive use | |
| Skinner 2004 | Folic acid | Pancreatic cancer | X | NA | | X | | T | X | X | height, diabetes, time period | alcohol consumption evaluated as mediating factor |
| Slatore 2008 | Folic acid | Lung cancer | X | X | T | X | | | | T | / | |
| Slatore 2008 | Vitamin C | Lung cancer | X | X | T | X | | | | T | / | |
| Timmermans 2011 | Folic acid | Gestational hypertension | X | NA | X | X | X | | | X | gestational age, parity, ethnicity, antenatal care | |
| Timmermans 2012 | Mediterranean diet | Birth weight | X | NA | X | X | NA | | | X | parity, height, folic acid use, sex of child, gestational age | Alcohol consumption is part of the Mediterranean diet scoring method. |

| | | | | | | | | | | | | |
|---|---|---|---|---|---|---|---|---|---|---|---|---|
| Tobias 2012 | Mediterranean diet | Gestational diabetes | X | NA | | X | NA | X | X | X | gravidity, sedentary time, parental history of type 2 diabetes | Alcohol consumption is part of the Mediterranean diet scoring method. |
| Tortosa 2007 | Mediterranean diet | Metabolic syndrome | X | X | | X | NA | X | X | NA | diabetes, hypertension, hypercholesterolemia, hypertriglyceridemia, metabolic syndrome | Weight/Obesity is part of the outcome. |
| Tortosa 2007 | Mediterranean diet | HDL-Cholesterol | X | X | | | NA | | | | / | Alcohol consumption is part of the Mediterranean diet scoring method. |
| Tortosa 2007 | Mediterranean diet | Systolic blood pressure | X | X | | | NA | | | | / | Alcohol consumption is part of the Mediterranean diet scoring method. |
| Tortosa 2007 | Mediterranean diet | Trigylcerides | X | X | | | NA | | | | / | Alcohol consumption is part of the Mediterranean diet scoring method. |
| Wang 2015 | Dairy | Systolic blood pressure | X | X | | X | NA | X | X | X | measurements of systolic or diastolic blood pressure at the beginning of each exam interval, diabetic status, the ratio of total cholesterol and HDL-cholesterol, TAG concentrations, use of anti-hyperlipidaemia/ hormone-replacement therapy/ oral contraceptive medications at the beginning of each exam interval, caffeinated coffee intake, modified DGAI score within each exam interval | Alcohol consumption is part of DGAI-scoring method. |
| Wang 2016 | Folic acid | Low birthweight | | NA | | | | | | | / | unadjusted effect estimate |
| Wang 2016 | Folic acid | Birth weight | | NA | | | | | | | / | unadjusted effect estimate |
| Wen 2016 | Folic acid | Pre-eclampsia | X | NA | X | X | | | | X | previous health problem (chronic hypertension, history of preeclampsia, diabetes), parity | |
| Yang 2016 | Calcium | Cardiovascular disease mortality | X | NA | X | X | X | X | X | X | whole grain, red and processed meats, total folate, dietary calcium intake, hormone replacement therapy | |
| Yu 2014/ SMHS | Healthy diet | All-cause mortality | X | NA | X | X | NA | X | X | X | multivitamin use; waist-to-hip ratio; history of cardiovascular disease, diabetes, or hypertension | alcohol consumption is part of the AHEI-2010 scoring method |
| Yu 2014/ SWHS | Healthy diet | All-cause mortality | X | NA | X | X | NA | X | X | X | multivitamin use; menopausal status and hormone therapy; waist-to-hip ratio; history of cardiovascular disease, diabetes, or hypertension | alcohol consumption is part of the AHEI-2010 scoring method |
| Zschäbitz 2013 | Folic acid | Colorectal cancer | X | NA | T | X | | X | T | X | race/ ethnicity, medical history of colonoscopy, postmenopausal hormone therapy use | alcohol consumption evaluated as mediating factor |

AHEI-2010: Alternate Healthy Eating Index 2010; BMI: body mass index; CVD: cardiovascular disease; DGAI: Dietary Guidelines Adherence Index; HDL: high density lipoprotein; HPFS: Health Professionals Follow-up Study; NA: not applicable; NHS: Nurses' Health Study; NHS II: Nurses' Health Study II; PSA: prostate-specific antigen; PUFA: polyunsaturated fatty acid; rMED: relative Mediterranean diet score; T: tested; TAG: triacylglycerol

**Table S8** Univariable meta-regression for PI/ECO similarity across pairs with binary outcomes

| **Variable** | **Estimate** | **95% confidence interval** |
|---|---|---|
| Intercept | 0.95 | 0.68 to 1.34 |
| PI/ECO_overall* | 1.03 | 0.86 to 1.22 |

PI/ECO: population, intervention/exposure, comparator, outcome;
One-level increase reflects less matching pairs ("more or less identical" to "similar but not identical"; "similar but not identical" to "broadly similar").

**Table S9** Multivariable meta-regression for PI/ECO similarity (by domain) across pairs with binary outcomes

| **Variable** | **Estimate** | **95% confidence interval** |
|---|---|---|
| Intercept | 0.95 | 0.55 to 1.64 |
| PI/ECO_population* | 0.99 | 0.85 to 1.16 |
| PI/ECO_intervention* | 1.02 | 0.82 to 1.27 |
| PI/ECO_outcome* | 1.03 | 0.76 to 1.41 |

PI/ECO: population, intervention/exposure, comparator, outcome;
One-level increase reflects less matching pairs in the respective domain ("more or less identical" to "similar but not identical"; "similar but not identical" to "broadly similar").

**Table S10** Univariable meta-regression for risk of bias rating with the RoB 2 tool across pairs with binary outcomes

| **Variable** | **Estimate** | **95% confidence interval** |
|---|---|---|
| Intercept | 1.19 | 0.89 to 1.58 |
| RoB2_overall* | 0.90 | 0.77 to 1.06 |

RoB 2: Risk of Bias 2 tool;
* One-level increase reflects an increased susceptibility to potential sources of bias according to the Risk of Bias 2 tool (low risk to some concerns, some concerns to high risk).

**Table S11** Univariable meta-regression for risk of bias rating with the ROBINS-E tool across pairs with binary outcomes

| **Variable** | **Estimate** | **95% confidence interval** |
|---|---|---|
| Intercept | 1.16 | 0.73 to 1.84 |
| ROBINS-E_overall* | 0.94 | 0.78 to 1.13 |

ROBINS-E: Risk Of Bias In Non-randomised Studies - of Exposure tool;
* One-level increase reflects an increased susceptibility to potential sources of bias according to the ROBINS-E tool (low risk to some concerns, some concerns to high risk).

**Table S12** Multivariable meta-regression for PI/ECO similarity and risk of bias rating across pairs with binary outcomes

| **Variable** | **Estimate** | **95% confidence interval** |
|---|---|---|
| Intercept | 1.54 | 0.91 to 3.50 |
| PI/ECO_overall | 1.05 | 0.83 to 1.18 |
| RoB2_overall | 0.87 | 0.74 to 1.06 |
| ROBINS-E_overall | 0.89 | 0.72 to 1.06 |

PI/ECO: population, intervention/exposure, comparator, outcome; RoB 2: Risk of Bias 2 tool; ROBINS-E: Risk Of Bias In Non-randomised Studies - of Exposure tool

**Table S13** Overlaps between study design pairs

| Randomised controlled trials that were included in two or more study design pairs: |
|---|
| Christian 2003 |
| Esposito 2009 |
| The Da Qing IGT and Diabetes Study |
| The Italian-American Clinical Trial of Nutritional Supplements and Age-Related Cataract (CTNS) |
| The Prevención con Dieta Mediterránea study (PREDIMED) |
| The Randomised controlled trial of low glycaemic index diet in pregnancy to prevent macrosomia (ROLO study) |
| The Physicians' Health Study II (PHS II) |
| The Selenium and Vitamin E Cancer Prevention Trial (SELECT) |
| The Women's Antioxidant Cardiovascular Study (WACS) |
| The Women's Antioxidant and Folic Acid Cardiovascular Study (WAFACS) |
| The Women's Health Initiative (WHI) |

| Cohort studies that were included in two or more study design pairs: |
|---|
| The Age-Related Eye Disease Study |
| The Cancer Prevention Study II Nutrition Cohort |
| The Danish National Birth cohort |
| The European Prospective Investigation into Cancer and Nutrition cohort study (EPIC) |
| The Health Professionals Follow-up study (HPFS) |
| The Norwegian Mother and Child Cohort Study |
| The Third National Health and Nutrition Examination Survey (NHANES III) |
| The Nurses Health Study (NHS) |
| The Nurses Health Study II (NHS II) |
| The Seguimiento Universidad de Navarra (SUN) dynamic cohort |
| The VITamins And Lifestyle cohort (VITAL) |
| The Women's Health Initiative Observational Study cohort (WHI-OS) |

**Figure S1** Risk of bias in individual randomised controlled trials

| Study | D1 | D2 | D3 | D4 | D5 | Overall |
|---|---|---|---|---|---|---|
| | Risk of bias domains | | | | | |
| Armitage 2010 / Folic acid / Lung cancer | + | + | + | + | + | + |
| Baron 2015 / Vitamin D / Nephrolithiasis | + | + | + | + | + | + |
| Barr 2000 / Dairy / Systolic blood pressure | + | + | - | + | - | - |
| Brough 2010 / Multivitamins / Preterm birth | + | - | - | + | - | - |
| Brunner 2011 / Vitamin D / Breast cancer | + | + | + | + | + | + |
| Burr 1989 / PUFA / MACCE | + | - | - | + | - | - |
| Chai 2012 / Apples / Body weight | - | X | - | - | - | X |
| Charles 2005 / Folic acid / Pre-eclampsia | - | + | + | + | - | - |
| Christian 2003 / Folic acid / Low birthweight | - | + | - | + | - | - |
| Christian 2003 / Folic acid / Mean birthweight | - | + | - | + | - | - |
| Czeizel 1994 / Folic acid / Neural tube defect | - | + | - | + | - | - |
| Czeizel 1998 / Folic acid / Cardiovascular defects | - | - | - | - | - | - |
| De Lorgeril 1999 / Mediterranean diet / Cancer mortality | + | - | - | + | - | - |
| Esposito 2009 / Mediterranean diet / HDL-Cholesterol | + | + | X | + | - | X |
| Esposito 2009 / Mediterranean diet / Systolic blood pressure | + | + | X | - | - | X |
| Esposito 2009 / Mediterranean diet / Triglycerides | + | + | X | + | - | X |
| Estruch 2018 / Mediterranean diet / All-cause mortality | - | - | + | + | + | - |
| Estruch 2018 / Nuts / Coronary heart disease | - | - | + | + | + | - |
| Estruch 2018 / Nuts / Stroke | - | - | + | + | + | - |
| Estruch 2018 / Mediterranean diet / Cardiovascular disease | - | - | + | + | + | - |
| Gaziano 2009 / Vitamin C / Prostate cancer | + | + | + | + | + | + |
| Gaziano 2009 / Vitamin E / Prostate cancer | + | + | + | + | + | + |
| Heinonen 1998 / ß-Carotene / Prostate cancer | + | + | - | + | + | - |
| Hollis 2011 / Vitamin D / Pre-eclampsia | + | + | - | + | - | - |
| Howard 2006 / Low-fat diet / All-cause mortality | - | - | + | + | - | - |
| Howard 2006 / Low-fat diet / Cardiovascular disease mortality | - | - | + | + | - | - |
| Hsia 2007 / Calcium / Cardiovascular disease mortality | + | + | - | + | - | - |
| Jackson 2006 / Calcium / All fractures | + | + | - | + | + | - |
| Karp 2013 / Selenium / Oesophageal cancer | + | + | - | + | - | - |
| Kirke 1992 / Multivitamins / Stillbirth | + | - | - | - | - | - |
| Lin 2009 / Vitamin C / Breast cancer | - | + | + | + | + | - |
| Lin 2009 / Vitamin C / Colorectal cancer | - | + | + | + | + | - |
| Lin 2009 / Vitamin C / Lung cancer | - | + | + | + | + | - |
| Lippman 2009 / Selenium / Colorectal cancer | + | + | + | + | + | + |

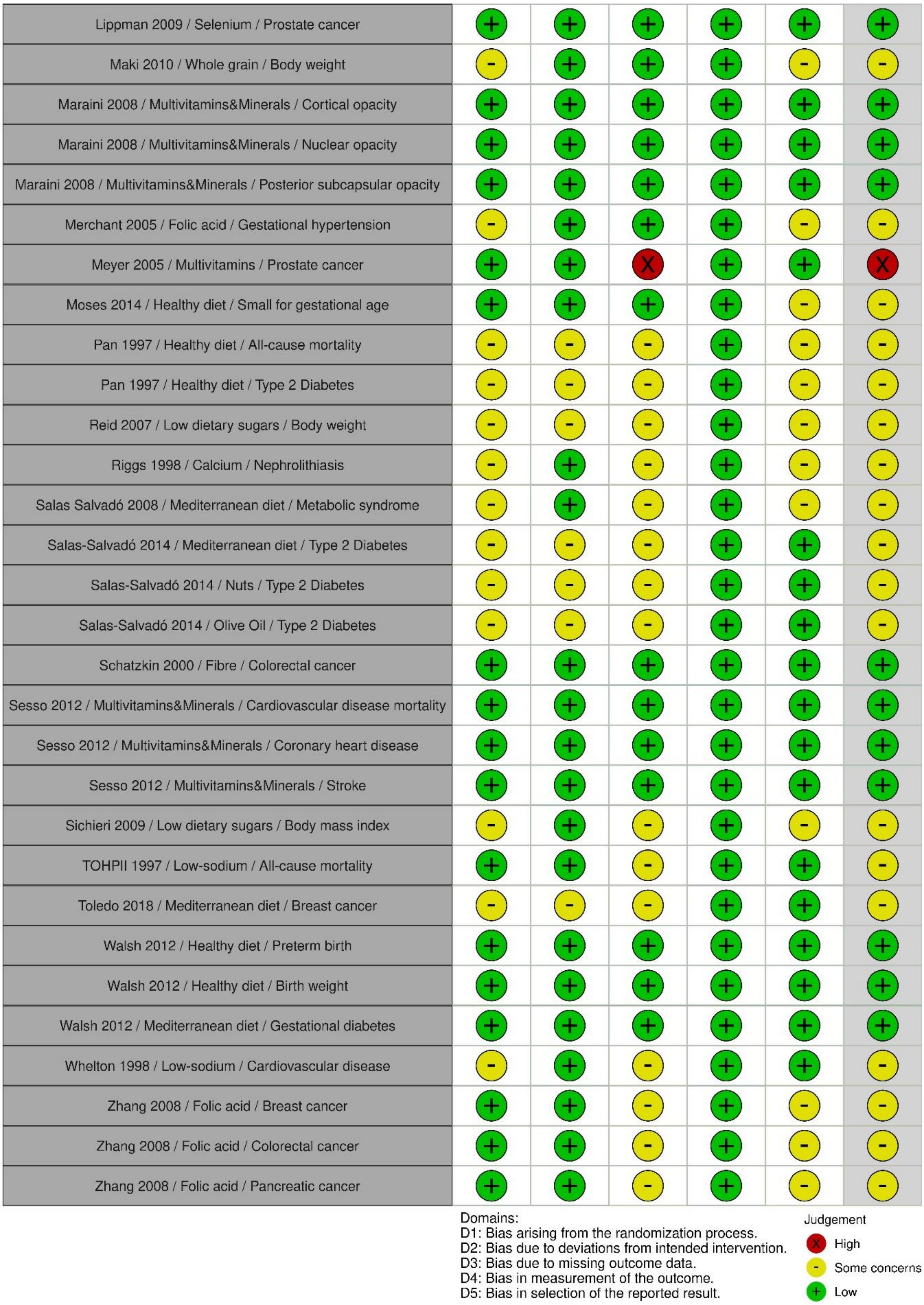


HDL: high density lipoprotein; MACCE: major adverse cardiac and cerebrovascular events; PUFA: polyunsaturated fatty acids; TOHP II: Trials of Hypertension Prevention, Phase II

**Figure S2** Risk of bias in randomised controlled trials (summary plot)

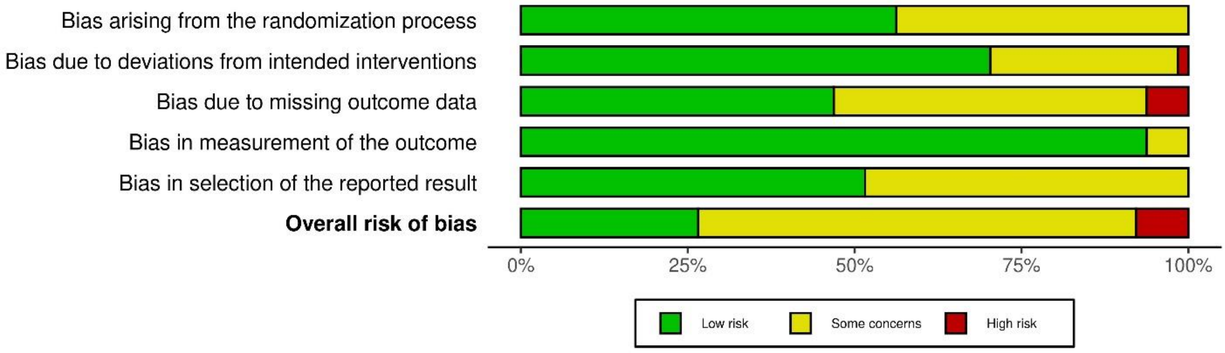


**Figure S3** Risk of bias in individual cohort studies

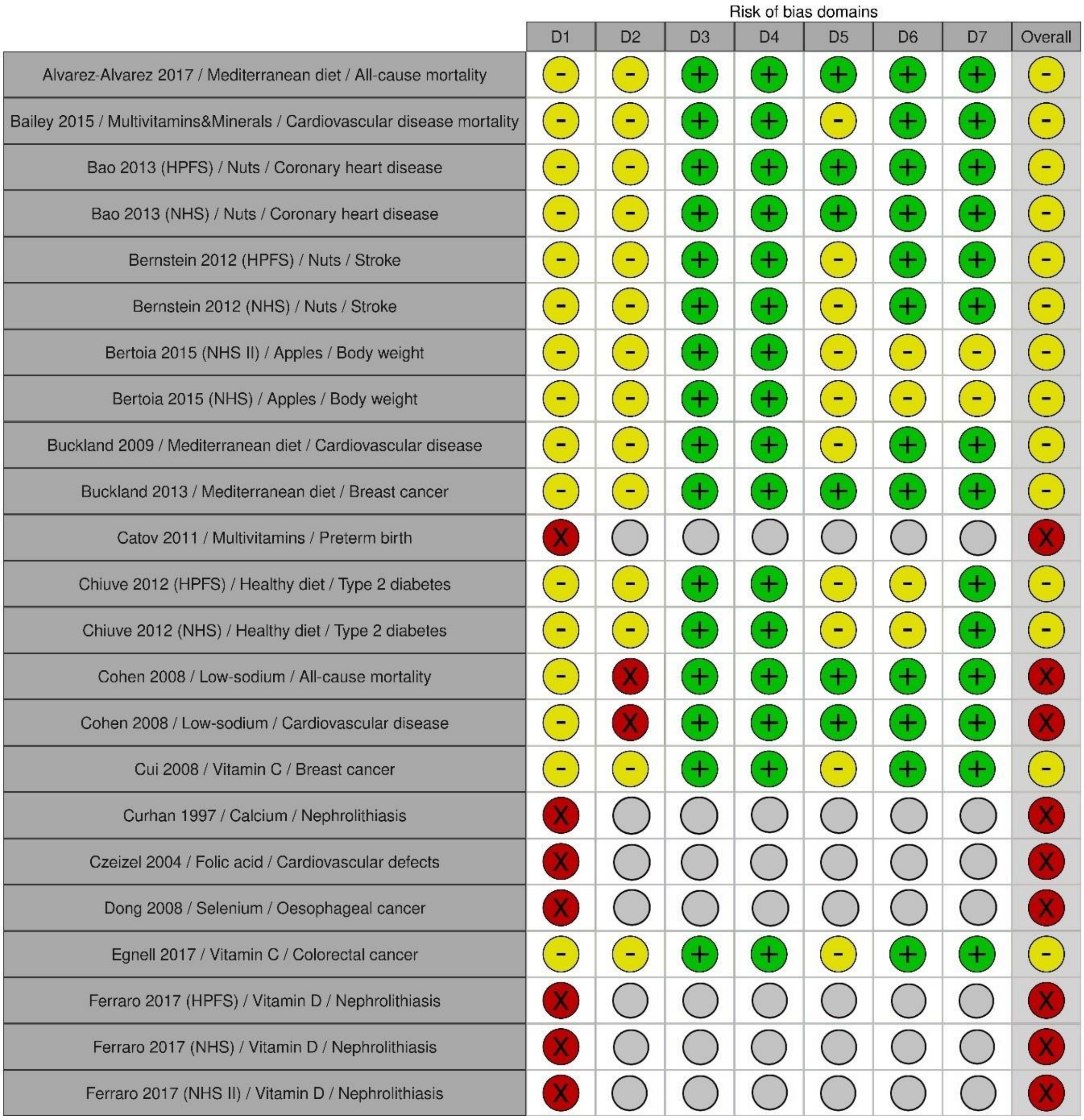

| Study | | | | | | | | |
|---|---|---|---|---|---|---|---|---|
| Gresham 2016 / Healthy diet / Small gestational age | X | | | | | | | X |
| Guasch-Ferre 2015 / PUFA / MACCE | - | - | + | + | - | + | + | - |
| Hansen 2014 / Selenium / Colorectal cancer | - | - | + | + | + | + | + | - |
| Haugen 2009 / Vitamin D / Pre-eclampsia | X | | | | | | | X |
| Hillesund 2014 / Healthy diet / Preterm birth | X | | | | | | | X |
| InterAct 2011 / Mediterranean diet / Type 2 Diabetes | - | - | + | + | - | + | + | - |
| InterAct 2011 / Olive Oil / Type 2 Diabetes | - | - | + | + | - | + | + | - |
| Kirsh 2006 / ß-Carotene / Prostate cancer | X | | | | | | | X |
| Kirsh 2006 / Vitamin C / Prostate cancer | X | | | | | | | X |
| Lassale 2016 / Mediterranean diet / Cancer mortality | - | - | + | + | - | + | + | - |
| Lawson 2007 / Multivitamins / Prostate cancer | - | - | + | + | - | + | + | - |
| Leosdottir 2005 / Low-fat diet / All-cause mortality | - | - | + | + | - | + | + | - |
| Leosdottir 2005 / Low-fat diet / Cardiovascular mortality | - | - | + | + | - | + | + | - |
| Liu 2003 / Whole grain / Body weight | - | - | + | + | - | X | + | X |
| Ludwig 2001 / Low dietary sugars / Body mass index | - | - | + | + | - | + | - | - |
| Maruti 2009 / Folic acid / Breast cancer | X | | | | | | | X |
| Michels 2005 (HPFS) / Fibre / Colorectal cancer | - | - | + | + | - | + | + | - |
| Michels 2005 (NHS) / Fibre / Colorectal cancer | - | - | + | + | - | + | + | - |
| Milton 2006 / Multivitamins&Minerals / Cortical opacity | X | | | | | | | X |
| Milton 2006 / Multivitamins&Minerals / Nuclear opacity | X | | | | | | | X |
| Milton 2006 / Multivitamins&Minerals / Posterior subcapsular opacity | X | | | | | | | X |
| Milunsky 1989 / Folic acid / Neural tube defect | ! | | | | | | | ! |
| Nohr 2014 / Multivitamins / Stillbirth | ! | | | | | | | ! |
| Pan 2013 (NHS II) / Nuts / Type 2 Diabetes | - | - | + | + | + | - | + | - |
| Pan 2013 (NHS) / Nuts / Type 2 Diabetes | - | - | + | + | + | - | + | - |
| Peters 2008 / Selenium / Prostate cancer | - | - | + | + | + | + | + | - |
| Prentice 2013 / Calcium+Vitamin D / All fractures | X | | | | | | | X |
| Rautiainen 2016 / Multivitamins&Minerals / Coronary heart disease | - | X | + | + | - | + | + | X |
| Rautiainen 2016 / Multivitamins&Minerals / Stroke | - | X | + | + | - | + | + | X |
| Robien 2007 / Vitamin D / Breast cancer | - | - | + | + | + | + | + | - |
| Rodriguez 2004 / Vitamin E / Prostate cancer | X | | | | | | | X |
| Schulze 2004 / Low dietary sugar / Body weight | X | | | | | | | X |
| Skinner 2004 / Folic acid / Pancreatic cancer | - | - | + | + | - | + | + | - |
| Slatore 2008 / Folic acid / Lung cancer | X | | | | | | | X |
| Slatore 2008 / Vitamin C / Lung cancer | X | | | | | | | X |
| Timmermans 2011 / Folic acid / Gestational diabetes | X | | | | | | | X |
| Timmermans 2012 / Healthy diet / Birth weight | X | | | | | | | X |

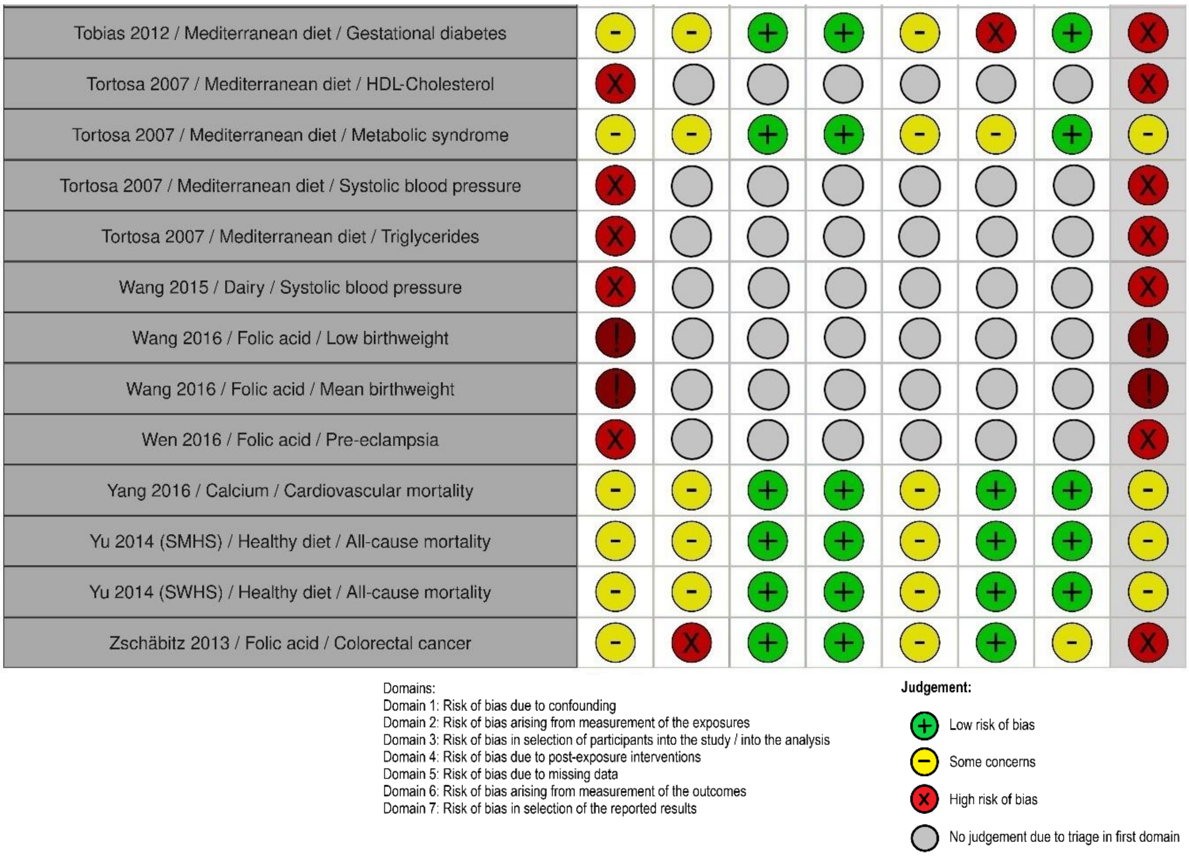


HDL: high density lipoprotein; HPFS: Health Professionals Follow-up Study; MACCE: major adverse cardiac and cerebrovascular events; NHS: Nurses' Health Study; NHS II: Nurses' Health Study II; PUFA: polyunsaturated fatty acid; SMHS: Shanghai Men’s Health Study; SWHS: Shanghai Women’s Health Study

**Figure S4** Risk of bias in cohort studies (summary plot)

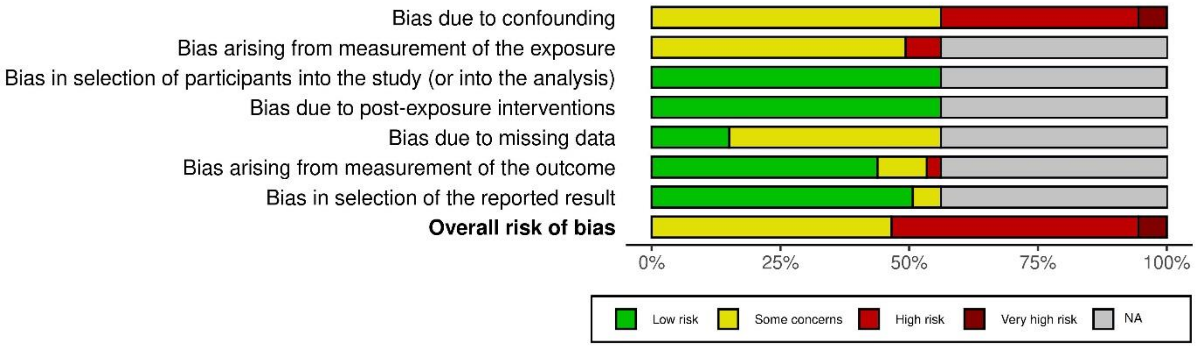

**Figure S5** Forest plot of the comparison between bodies of evidence from randomised controlled trials versus those from cohort studies for continuous outcomes using difference of standardised mean difference

| Reference Pairs | Intervention/Exposure | Outcome | SMD (RCT) | 95%−CI | SMD (cohort) | 95%−CI | DSMD | 95%−CI | Weight |
|---|---|---|---|---|---|---|---|---|---|
| Barr 2000 / Wang 2015 | Dairy | Systolic blood pressure | 0.12 | [−0.16; 0.4] | −0.25 | [−0.35; −0.14] | 0.37 | [ 0.07; 0.67] | 14.3% |
| Christian 2003 / Wang 2016 | Folic acid | Birth weight | 0.00 | [−0.11; 0.11] | 0.18 | [0.00; 0.35] | −0.18 | [−0.39; 0.03] | 14.6% |
| Esposito 2009 / Tortosa 2007 | Mediterranean diet | HDL−cholesterol | 1.19 | [0.90; 1.48] | 0.02 | [−0.10; 0.14] | 1.17 | [ 0.86; 1.48] | 14.3% |
| Esposito 2009 / Tortosa 2007 | Mediterranean diet | Triglycerides | −0.99 | [−1.28; −0.71] | −0.05 | [−0.17; 0.08] | −0.94 | [−1.25; −0.63] | 14.3% |
| Esposito 2009 / Tortosa 2007 | Mediterranean diet | Systolic blood pressure | −0.76 | [−1.03; −0.48] | 0.06 | [−0.06; 0.18] | −0.82 | [−1.12; −0.52] | 14.3% |
| Maki 2010 / Liu 2003 | Whole grain | Body weight | −1.33 | [−1.66; −1.00] | −0.12 | [−0.14; −0.10] | −1.21 | [−1.54; −0.88] | 14.2% |
| Reid 2007 / Schulze 2004 | Low dietary sugar | Body weight | −0.47 | [−0.81; −0.12] | −0.25 | [−0.32; −0.17] | −0.22 | [−0.57; 0.13] | 14.1% |
| **Random effects model** | | | | | | | **−0.26** | **[−0.87; 0.35]** | **100.0%** |
| **Prediction interval** | | | | | | | | **[−2.50; 1.98]** | |

Heterogeneity: $I^2 = 96\%$, $\tau^2 = 0.6623$, $p < 0.01$

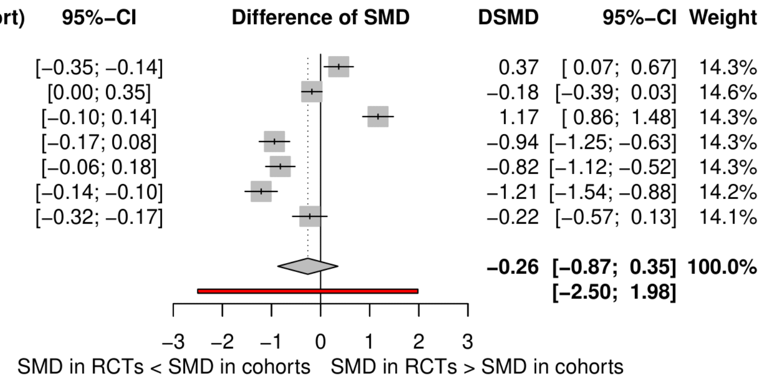


CI: confidence interval; DSMD: difference of standardised mean differences; HDL: high density lipoprotein; RCT: randomised controlled trial; SMD: standardised mean difference

**Figure S6** Forest plot of the comparison between study design pairs with binary outcomes / subgroup analysis by dietary intervention/exposure

| Reference Pairs | Intervention/Exposure | Outcome | RR (RCT) | 95%–CI | RR (cohort) | 95%–CI | RRR | 95%–CI |
|---|---|---|---|---|---|---|---|---|
| ***(Micro–)nutrients*** | | | | | | | | |
| Armitage 2010 / Slatore 2008 | Folic acid | Lung cancer | 0.95 | [0.74; 1.22] | 0.92 | [0.40; 2.11] | 1.04 | [0.44; 2.47] |
| Baron 2015 / Ferraro 2017 | Vitamin D | Nephrolithiasis | 0.68 | [0.38; 1.21] | 1.10 | [0.99; 1.22] | 0.62 | [0.34; 1.11] |
| Brough 2010 / Catov 2011 | Multivitamins | Preterm birth | 1.09 | [0.43; 2.77] | 0.89 | [0.80; 0.99] | 1.22 | [0.48; 3.13] |
| Brunner 2011 / Robien 2007 | Vitamin D | Breast Cancer | 0.96 | [0.85; 1.09] | 0.96 | [0.91; 1.01] | 1.00 | [0.87; 1.14] |
| Burr 1989 / Guasch–Ferre 2015 | PUFA | Major cardiovascular events | 0.99 | [0.87; 1.12] | 0.68 | [0.48; 0.96] | 1.46 | [1.01; 2.11] |
| Charles 2005 / Wen 2016 | Folic acid | Pre–eclampsia | 0.61 | [0.34; 1.08] | 0.59 | [0.34; 1.02] | 1.04 | [0.47; 2.31] |
| Christian 2003 / Wang 2016 | Folic acid | Low birthweight | 0.96 | [0.85; 1.09] | 0.35 | [0.05; 2.49] | 2.74 | [0.39; 19.43] |
| Czeizel 1994 / Milunsky 1989 | Folic acid | Neural tube defect | 0.07 | [0.00; 1.32] | 0.29 | [0.15; 0.55] | 0.26 | [0.01; 4.89] |
| Czeizel 1998 / Czeizel 2004 | Folic acid | Congenital heart defects | 0.48 | [0.23; 1.03] | 0.60 | [0.38; 0.96] | 0.80 | [0.33; 1.93] |
| Gaziano 2009 / Kirsh 2006 | Vitamin C | Prostate cancer | 1.01 | [0.90; 1.14] | 1.01 | [0.94; 1.09] | 1.00 | [0.87; 1.15] |
| Gaziano 2009 / Rodriguez 2004 | Vitamin E | Prostate cancer | 0.96 | [0.85; 1.08] | 1.00 | [0.97; 1.03] | 0.96 | [0.85; 1.09] |
| Heinonen 1998 / Kirsh 2006 | ß–carotene | Prostate cancer | 1.24 | [0.96; 1.59] | 0.55 | [0.10; 2.92] | 2.24 | [0.41; 12.36] |
| Hollis 2011 / Haugen 2009 | Vitamin D | Pre–eclampsia | 0.50 | [0.20; 1.23] | 0.68 | [0.47; 0.99] | 0.74 | [0.28; 1.97] |
| Hsia 2007 / Yang 2016 | Calcium | Cardiovascular disease mortality | 1.01 | [0.79; 1.29] | 0.85 | [0.78; 0.92] | 1.19 | [0.92; 1.54] |
| Jackson 2006 / Prentice 2013 | Calcium | Fractures | 0.97 | [0.92; 1.03] | 1.07 | [1.01; 1.14] | 0.91 | [0.83; 0.98] |
| Karp 2013 / Dong 2008 | Selenium | Oesophageal cancer | 1.50 | [0.06; 36.86] | 0.02 | [0.00; 1.94] | 75.00 | [0.28; 20175.93] |
| Kirke 1992 / Nohr 2014 | Multivitamins | Stillbirth | 2.43 | [0.12; 50.05] | 0.82 | [0.62; 1.10] | 2.96 | [0.14; 61.35] |
| Lin 2009 / Cui 2008 | Vitamin C | Breast cancer | 1.11 | [0.87; 1.41] | 1.08 | [1.02; 1.14] | 1.03 | [0.80; 1.32] |
| Lin 2009 / Egnell 2017 | Vitamin C | Colorectal cancer | 0.76 | [0.42; 1.38] | 0.00 | [0.00; 11.67] | 760.00 | [0.06; 9833094.45] |
| Lin 2009 / Slatore 2008 | Vitamin C | Lung cancer | 1.84 | [1.14; 2.97] | 0.95 | [0.74; 1.21] | 1.94 | [1.13; 3.32] |
| Lippman 2009 / Hansen 2013 | Selenium | Colorectal cancer | 1.04 | [0.73; 1.48] | 0.52 | [0.32; 0.86] | 2.00 | [1.09; 3.67] |
| Lippman 2009 / Peters 2008 | Selenium | Prostate cancer | 1.03 | [0.90; 1.18] | 0.83 | [0.37; 1.89] | 1.24 | [0.54; 2.84] |
| Maraini 2008 / Milton 2006 | Multivitamins/Minerals | Nuclear cataract | 0.66 | [0.50; 0.88] | 0.75 | [0.61; 0.91] | 0.88 | [0.62; 1.24] |
| Maraini 2008 / Milton 2006 | Multivitamins/Minerals | Cortical cataract | 0.78 | [0.60; 1.02] | 0.91 | [0.68; 1.22] | 0.86 | [0.58; 1.27] |
| Maraini 2008 / Milton 2006 | Multivitamins/Minerals | Posterior subcapsular cataract | 2.00 | [1.35; 2.98] | 1.02 | [0.77; 1.36] | 1.96 | [1.20; 3.19] |
| Merchant 2005 / Timmermans 2011 | Folic acid | Gestational hypertension | 0.63 | [0.42; 0.94] | 1.21 | [0.92; 1.57] | 0.52 | [0.32; 0.85] |
| Meyer 2005 / Lawson 2007 | Multivitamins | Prostate cancer | 0.90 | [0.62; 1.33] | 1.03 | [0.99; 1.07] | 0.87 | [0.60; 1.28] |
| Riggs 1998 / Curhan 1997 | Calcium | Nephrolithiasis | 0.33 | [0.01; 7.96] | 1.59 | [0.95; 2.64] | 0.21 | [0.01; 6.10] |
| Schatzkin 2000 / Michels 2005 | Fiber | Colorectal cancer | 2.47 | [0.78; 7.85] | 0.94 | [0.74; 1.20] | 2.63 | [0.81; 8.55] |
| Sesso 2012 / Bailey 2015 | Multivitamins/Minerals | Cardiovascular disease mortality | 0.95 | [0.83; 1.09] | 0.65 | [0.49; 0.85] | 1.46 | [1.07; 1.99] |
| Sesso 2012 / Rautiainen 2016 | Multivitamins/Minerals | Coronary heart disease | 0.93 | [0.79; 1.09] | 0.88 | [0.74; 1.04] | 1.06 | [0.84; 1.34] |
| Sesso 2012 / Rautiainen 2016 | Multivitamins/Minerals | Stroke | 1.06 | [0.89; 1.25] | 0.94 | [0.79; 1.13] | 1.13 | [0.88; 1.44] |
| TOHP II 1997 / Cohen 2008 | Low–sodium | All–cause mortality | 0.67 | [0.36; 1.25] | 1.06 | [0.99; 1.14] | 0.63 | [0.34; 1.18] |
| Whelton 1998 / Cohen 2008 | Low–sodium | Cardiovascular disease | 0.78 | [0.52; 1.18] | 1.14 | [0.99; 1.30] | 0.68 | [0.44; 1.05] |
| Zhang 2008 / Maruti 2009 | Folic acid | Breast cancer | 0.83 | [0.61; 1.14] | 0.91 | [0.35; 2.36] | 0.91 | [0.33; 2.50] |
| Zhang 2008 / Skinner 2004 | Folic acid | Pancreatic cancer | 1.50 | [0.42; 5.31] | 5.86 | [0.25; 139.27] | 0.26 | [0.01; 7.72] |
| Zhang 2008 / Zschäbitz 2013 | Folic acid | Colorectal cancer | 0.82 | [0.44; 1.52] | 0.89 | [0.25; 3.20] | 0.92 | [0.22; 3.79] |
| **Random effects model** | | | | | | | **1.04** | **[0.93; 1.17]** |
| **Prediction interval** | | | | | | | | **[0.65; 1.65]** |
| Heterogeneity: $I^2 = 45\%$, $\tau^2 = 0.0487$, $p < 0.01$ | | | | | | | | |
| ***Food group*** | | | | | | | | |
| Estruch 2018 / Bao 2013 | Nuts | Coronary heart disease | 0.79 | [0.55; 1.14] | 0.81 | [0.75; 0.88] | 0.98 | [0.67; 1.42] |
| Estruch 2018 / Bernstein 2012 | Nuts | Stroke | 0.62 | [0.44; 0.87] | 0.90 | [0.79; 1.02] | 0.69 | [0.48; 0.99] |
| Salas–Salvado 2014 / InterAct 2011 | Olive oil | Type 2 diabetes | 0.89 | [0.82; 0.96] | 0.88 | [0.81; 0.95] | 1.01 | [0.90; 1.13] |
| Salas–Salvado 2014 / Pan 2013 | Nuts | Type 2 diabetes | 0.86 | [0.68; 1.08] | 0.88 | [0.83; 0.94] | 0.98 | [0.77; 1.24] |
| **Random effects model** | | | | | | | **0.95** | **[0.83; 1.09]** |
| **Prediction interval** | | | | | | | | **[0.60; 1.50]** |
| Heterogeneity: $I^2 = 23\%$, $\tau^2 = 0.0063$, $p = 0.27$ | | | | | | | | |
| ***Dietary pattern*** | | | | | | | | |
| de Lorgeril 1998 / Lassale 2016 | Mediterranean diet | Cancer mortality | 0.75 | [0.17; 3.33] | 0.82 | [0.77; 0.88] | 0.91 | [0.21; 4.05] |
| Estruch 2018 / Alvarez–Alvarez 2017 | Mediterranean diet | All–cause mortality | 1.01 | [0.81; 1.25] | 0.79 | [0.51; 1.22] | 1.28 | [0.79; 2.08] |
| Estruch 2018 / Buckland 2009 | Mediterranean diet | Cardiovascular disease | 0.81 | [0.64; 1.02] | 0.60 | [0.47; 0.77] | 1.35 | [0.96; 1.90] |
| Howard 2006 / Leosdottir 2005 | Low–fat diet | All–cause mortality | 0.98 | [0.90; 1.06] | 1.12 | [0.81; 1.56] | 0.87 | [0.62; 1.22] |
| Howard 2006 / Leosdottir 2005 | Low–fat diet | Cardiovascular disease mortality | 0.99 | [0.81; 1.20] | 1.82 | [0.85; 3.85] | 0.54 | [0.25; 1.19] |
| Moses 2014 / Gresham 2016 | Healthy diet | Small for gestational age | 0.86 | [0.48; 1.54] | 0.42 | [0.11; 1.09] | 2.05 | [0.57; 7.42] |
| Pan 1997 / Chiuve 2012 | Healthy diet | Type 2 diabetes | 0.65 | [0.52; 0.81] | 0.82 | [0.76; 0.89] | 0.79 | [0.63; 1.00] |
| Pan 1997 / Yu 2014 | Healthy diet | All–cause mortality | 1.02 | [0.21; 4.98] | 0.74 | [0.63; 0.86] | 1.38 | [0.28; 6.76] |
| Salas–Salvadó 2008 / Tortosa 2007 | Mediterranean diet | Metabolic syndrome | 0.90 | [0.82; 1.00] | 0.39 | [0.14; 0.81] | 2.32 | [0.96; 5.61] |
| Salas–Salvado 2014 / InterAct 2011 | Mediterranean diet | Type 2 diabetes | 0.69 | [0.53; 0.91] | 0.88 | [0.79; 0.97] | 0.78 | [0.59; 1.05] |
| Toledo 2018 / Buckland 2013 | Mediterranean diet | Breast cancer | 0.41 | [0.19; 0.86] | 0.94 | [0.88; 1.00] | 0.44 | [0.20; 0.93] |
| Walsh 2012 / Hillesund 2014 | Healthy diet | Preterm birth | 0.39 | [0.10; 1.46] | 0.91 | [0.80; 1.03] | 0.43 | [0.11; 1.65] |
| Walsh 2012 / Tobias 2012 | Mediterranean diet | Gestational diabetes | 0.71 | [0.35; 1.45] | 0.76 | [0.60; 0.95] | 0.93 | [0.44; 1.97] |
| **Random effects model** | | | | | | | **0.93** | **[0.74; 1.17]** |
| **Prediction interval** | | | | | | | | **[0.48; 1.80]** |
| Heterogeneity: $I^2 = 46\%$, $\tau^2 = 0.0753$, $p = 0.04$ | | | | | | | | |
| Test for subgroup differences: $\chi^2_2 = 1.26$, df = 2 ($p = 0.53$) | | | | | | | | |

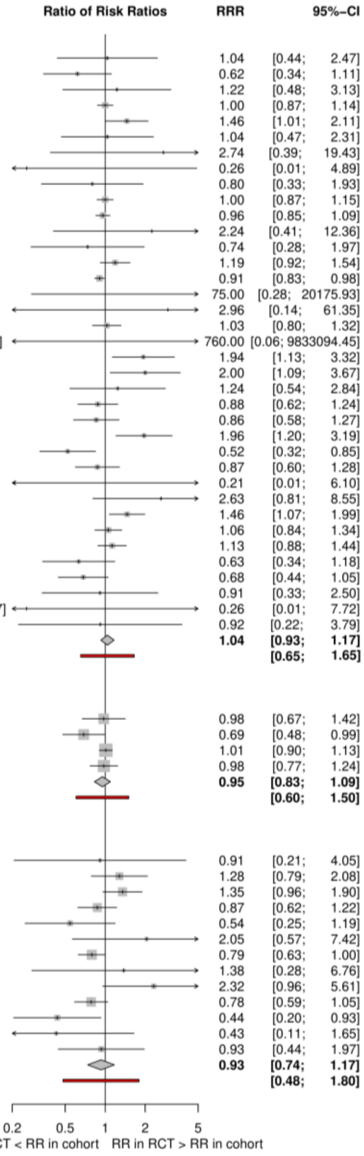


CI: confidence interval; PUFA: polyunsaturated fatty acid; RCT: randomised controlled trial; RR: risk ratio; RRR: ratio of risk ratios; TOHP II: Trials of Hypertension Prevention, Phase II

**Figure S7** Forest plot of the comparison between study design pairs with binary outcomes / subgroup analysis by type of intake

| Reference Pairs | Intervention/Exposure | Outcome | RR (RCT) | 95%–CI | RR (cohort) | 95%–CI | RRR | 95%–CI |
|---|---|---|---|---|---|---|---|---|
| ***Intake*** | | | | | | | | |
| Burr 1989 / Guasch–Ferre 2015 | PUFA | Major cardiovascular events | 0.99 | [0.87; 1.12] | 0.68 | [0.48; 0.96] | 1.46 | [1.01; 2.11] |
| de Lorgeril 1998 / Lassale 2016 | Mediterranean diet | Cancer mortality | 0.75 | [0.17; 3.33] | 0.82 | [0.77; 0.88] | 0.91 | [0.21; 4.05] |
| Estruch 2018 / Alvarez–Alvarez 2017 | Mediterranean diet | All–cause mortality | 1.01 | [0.81; 1.25] | 0.79 | [0.51; 1.22] | 1.28 | [0.79; 2.08] |
| Estruch 2018 / Bao 2013 | Nuts | Coronary heart disease | 0.79 | [0.55; 1.14] | 0.81 | [0.75; 0.88] | 0.98 | [0.67; 1.42] |
| Estruch 2018 / Bernstein 2012 | Nuts | Stroke | 0.62 | [0.44; 0.87] | 0.90 | [0.79; 1.02] | 0.69 | [0.48; 0.99] |
| Estruch 2018 / Buckland 2009 | Mediterranean diet | Cardiovascular disease | 0.81 | [0.64; 1.02] | 0.60 | [0.47; 0.77] | 1.35 | [0.96; 1.90] |
| Howard 2006 / Leosdottir 2005 | Low–fat diet | All–cause mortality | 0.98 | [0.90; 1.06] | 1.12 | [0.81; 1.56] | 0.87 | [0.62; 1.22] |
| Howard 2006 / Leosdottir 2005 | Low–fat diet | Cardiovascular disease mortality | 0.99 | [0.81; 1.20] | 1.82 | [0.85; 3.85] | 0.54 | [0.25; 1.19] |
| Moses 2014 / Gresham 2016 | Healthy diet | Small for gestational age | 0.86 | [0.48; 1.54] | 0.42 | [0.11; 1.09] | 2.05 | [0.57; 7.42] |
| Pan 1997 / Chiuve 2012 | Healthy diet | Type 2 diabetes | 0.65 | [0.52; 0.81] | 0.82 | [0.76; 0.89] | 0.79 | [0.63; 1.00] |
| Pan 1997 / Yu 2014 | Healthy diet | All–cause mortality | 1.02 | [0.21; 4.98] | 0.74 | [0.63; 0.86] | 1.38 | [0.28; 6.76] |
| Salas–Salvadó 2008 / Tortosa 2007 | Mediterranean diet | Metabolic syndrome | 0.90 | [0.82; 1.00] | 0.39 | [0.14; 0.81] | 2.32 | [0.96; 5.61] |
| Salas–Salvado 2014 / InterAct 2011 | Mediterranean diet | Type 2 diabetes | 0.69 | [0.53; 0.91] | 0.88 | [0.79; 0.97] | 0.78 | [0.59; 1.05] |
| Salas–Salvado 2014 / InterAct 2011 | Olive oil | Type 2 diabetes | 0.89 | [0.82; 0.96] | 0.88 | [0.81; 0.95] | 1.01 | [0.90; 1.13] |
| Salas–Salvado 2014 / Pan 2013 | Nuts | Type 2 diabetes | 0.86 | [0.68; 1.08] | 0.88 | [0.83; 0.94] | 0.98 | [0.77; 1.24] |
| Schatzkin 2000 / Michels 2005 | Fiber | Colorectal cancer | 2.47 | [0.78; 7.85] | 0.94 | [0.74; 1.20] | 2.63 | [0.81; 8.55] |
| TOHP II 1997 / Cohen 2008 | Low–sodium | All–cause mortality | 0.67 | [0.36; 1.25] | 1.06 | [0.99; 1.14] | 0.63 | [0.34; 1.18] |
| Toledo 2018 / Buckland 2013 | Mediterranean diet | Breast cancer | 0.41 | [0.19; 0.86] | 0.94 | [0.88; 1.00] | 0.44 | [0.20; 0.93] |
| Walsh 2012 / Hillesund 2014 | Healthy diet | Preterm birth | 0.39 | [0.10; 1.46] | 0.91 | [0.80; 1.03] | 0.43 | [0.11; 1.65] |
| Walsh 2012 / Tobias 2012 | Mediterranean diet | Gestational diabetes | 0.71 | [0.35; 1.45] | 0.76 | [0.60; 0.95] | 0.93 | [0.44; 1.97] |
| Whelton 1998 / Cohen 2008 | Low–sodium | Cardiovascular disease | 0.78 | [0.52; 1.18] | 1.14 | [0.99; 1.30] | 0.68 | [0.44; 1.05] |
| **Random effects model** | | | | | | | **0.94** | **[0.80; 1.10]** |
| **Prediction interval** | | | | | | | | **[0.54; 1.64]** |
| Heterogeneity: $I^2 = 48\%$, $\tau^2 = 0.0644$, $p < 0.01$ | | | | | | | | |
| ***Supplementation*** | | | | | | | | |
| Armitage 2010 / Slatore 2008 | Folic acid | Lung cancer | 0.95 | [0.74; 1.22] | 0.92 | [0.40; 2.11] | 1.04 | [0.44; 2.47] |
| Baron 2015 / Ferraro 2017 | Vitamin D | Nephrolithiasis | 0.68 | [0.38; 1.21] | 1.10 | [0.99; 1.22] | 0.62 | [0.34; 1.11] |
| Brough 2010 / Catov 2011 | Multivitamins | Preterm birth | 1.09 | [0.43; 2.77] | 0.89 | [0.80; 0.99] | 1.22 | [0.48; 3.13] |
| Brunner 2011 / Robien 2007 | Vitamin D | Breast Cancer | 0.96 | [0.85; 1.09] | 0.96 | [0.91; 1.01] | 1.00 | [0.87; 1.14] |
| Charles 2005 / Wen 2016 | Folic acid | Pre–eclampsia | 0.61 | [0.34; 1.08] | 0.59 | [0.34; 1.02] | 1.04 | [0.47; 2.31] |
| Christian 2003 / Wang 2016 | Folic acid | Low birthweight | 0.96 | [0.85; 1.09] | 0.35 | [0.05; 2.49] | 2.74 | [0.39; 19.43] |
| Czeizel 1994 / Milunsky 1989 | Folic acid | Neural tube defect | 0.07 | [0.00; 1.32] | 0.29 | [0.15; 0.55] | 0.26 | [0.01; 4.89] |
| Czeizel 1998 / Czeizel 2004 | Folic acid | Congenital heart defects | 0.48 | [0.23; 1.03] | 0.60 | [0.38; 0.96] | 0.80 | [0.33; 1.93] |
| Gaziano 2009 / Kirsh 2006 | Vitamin C | Prostate cancer | 1.01 | [0.90; 1.14] | 1.01 | [0.94; 1.09] | 1.00 | [0.87; 1.15] |
| Gaziano 2009 / Rodriguez 2004 | Vitamin E | Prostate cancer | 0.96 | [0.85; 1.08] | 1.00 | [0.97; 1.03] | 0.96 | [0.85; 1.09] |
| Heinonen 1998 / Kirsh 2006 | ß–carotene | Prostate cancer | 1.24 | [0.96; 1.59] | 0.55 | [0.10; 2.92] | 2.24 | [0.41; 12.36] |
| Hollis 2011 / Haugen 2009 | Vitamin D | Pre–eclampsia | 0.50 | [0.20; 1.23] | 0.68 | [0.47; 0.99] | 0.74 | [0.28; 1.97] |
| Hsia 2007 / Yang 2016 | Calcium | Cardiovascular disease mortality | 1.01 | [0.79; 1.29] | 0.85 | [0.78; 0.92] | 1.19 | [0.92; 1.54] |
| Jackson 2006 / Prentice 2013 | Calcium | Fractures | 0.97 | [0.92; 1.03] | 1.07 | [1.01; 1.14] | 0.91 | [0.83; 0.98] |
| Karp 2013 / Dong 2008 | Selenium | Oesophageal cancer | 1.50 | [0.06; 36.86] | 0.02 | [0.00; 1.94] | 75.00 | [0.28; 20175.93] |
| Kirke 1992 / Nohr 2014 | Multivitamins | Stillbirth | 2.43 | [0.12; 50.05] | 0.82 | [0.62; 1.10] | 2.96 | [0.14; 61.35] |
| Lin 2009 / Cui 2008 | Vitamin C | Breast cancer | 1.11 | [0.87; 1.41] | 1.08 | [1.02; 1.14] | 1.03 | [0.80; 1.32] |
| Lin 2009 / Egnell 2017 | Vitamin C | Colorectal cancer | 0.76 | [0.42; 1.38] | 0.00 | [0.00; 11.67] | 760.00 | [0.06; 9833094.45] |
| Lin 2009 / Slatore 2008 | Vitamin C | Lung cancer | 1.84 | [1.14; 2.97] | 0.95 | [0.74; 1.21] | 1.94 | [1.13; 3.32] |
| Lippman 2009 / Hansen 2013 | Selenium | Colorectal cancer | 1.04 | [0.73; 1.48] | 0.52 | [0.32; 0.86] | 2.00 | [1.09; 3.67] |
| Lippman 2009 / Peters 2008 | Selenium | Prostate cancer | 1.03 | [0.90; 1.18] | 0.83 | [0.37; 1.89] | 1.24 | [0.54; 2.84] |
| Maraini 2008 / Milton 2006 | Multivitamins/Minerals | Nuclear cataract | 0.66 | [0.50; 0.88] | 0.75 | [0.61; 0.91] | 0.88 | [0.62; 1.24] |
| Maraini 2008 / Milton 2006 | Multivitamins/Minerals | Cortical cataract | 0.78 | [0.60; 1.02] | 0.91 | [0.68; 1.22] | 0.86 | [0.58; 1.27] |
| Maraini 2008 / Milton 2006 | Multivitamins/Minerals | Posterior subcapsular cataract | 2.00 | [1.35; 2.98] | 1.02 | [0.77; 1.36] | 1.96 | [1.20; 3.19] |
| Merchant 2005 / Timmermans 2011 | Folic acid | Gestational hypertension | 0.63 | [0.42; 0.94] | 1.21 | [0.92; 1.57] | 0.52 | [0.32; 0.85] |
| Meyer 2005 / Lawson 2007 | Multivitamins | Prostate cancer | 0.90 | [0.62; 1.33] | 1.03 | [0.99; 1.07] | 0.87 | [0.60; 1.28] |
| Riggs 1998 / Curhan 1997 | Calcium | Nephrolithiasis | 0.33 | [0.01; 7.96] | 1.59 | [0.95; 2.64] | 0.21 | [0.01; 6.10] |
| Sesso 2012 / Bailey 2015 | Multivitamins/Minerals | Cardiovascular disease mortality | 0.95 | [0.83; 1.09] | 0.65 | [0.49; 0.85] | 1.46 | [1.07; 1.99] |
| Sesso 2012 / Rautiainen 2016 | Multivitamins/Minerals | Coronary heart disease | 0.93 | [0.79; 1.09] | 0.88 | [0.74; 1.04] | 1.06 | [0.84; 1.34] |
| Sesso 2012 / Rautiainen 2016 | Multivitamins/Minerals | Stroke | 1.06 | [0.89; 1.25] | 0.94 | [0.79; 1.13] | 1.13 | [0.88; 1.44] |
| Zhang 2008 / Maruti 2009 | Folic acid | Breast cancer | 0.83 | [0.61; 1.14] | 0.91 | [0.35; 2.36] | 0.91 | [0.33; 2.50] |
| Zhang 2008 / Skinner 2004 | Folic acid | Pancreatic cancer | 1.50 | [0.42; 5.31] | 5.86 | [0.25; 139.27] | 0.26 | [0.01; 7.72] |
| Zhang 2008 / Zschäbitz 2013 | Folic acid | Colorectal cancer | 0.82 | [0.44; 1.52] | 0.89 | [0.25; 3.20] | 0.92 | [0.22; 3.79] |
| **Random effects model** | | | | | | | **1.04** | **[0.93; 1.16]** |
| **Prediction interval** | | | | | | | | **[0.70; 1.56]** |
| Heterogeneity: $I^2 = 40\%$, $\tau^2 = 0.0355$, $p = 0.01$ | | | | | | | | |
| Test for subgroup differences: $\chi^2_1 = 1.17$, df = 1 ($p = 0.28$) | | | | | | | | |

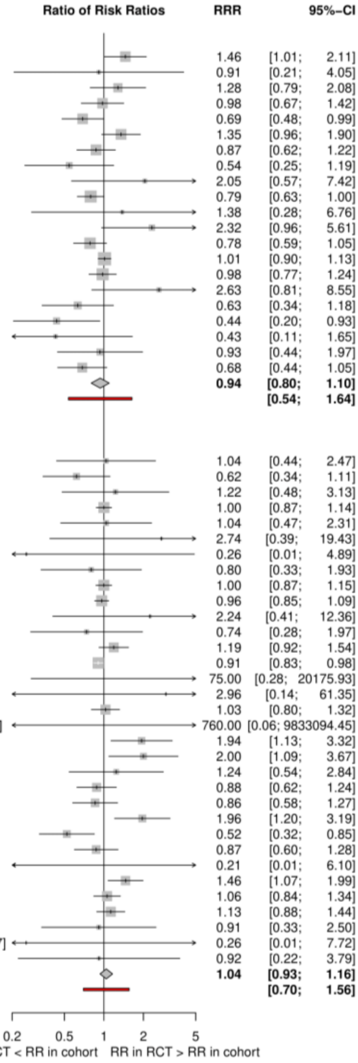


CI: confidence interval; PUFA: polyunsaturated fatty acid; RCT: randomised controlled trial; RR: risk ratio; RRR: ratio of risk ratios; TOHP II: Trials of Hypertension Prevention, Phase II

**Figure S8** Forest plot of the comparison between study design pairs with binary outcomes / subgroup analysis by outcome

| Reference Pairs | Intervention/Exposure | Outcome | RR (RCT) | 95%-CI | RR (cohort) | 95%-CI | RRR | 95%-CI |
|---|---|---|---|---|---|---|---|---|
| ***All-cause mortality*** | | | | | | | | |
| Estruch 2018 / Alvarez-Alvarez 2017 | Mediterranean diet | All-cause mortality | 1.01 | [0.81; 1.25] | 0.79 | [0.51; 1.22] | 1.28 | [0.79; 2.08] |
| Howard 2006 / Leosdottir 2005 | Low-fat diet | All-cause mortality | 0.98 | [0.90; 1.06] | 1.12 | [0.81; 1.56] | 0.87 | [0.62; 1.22] |
| Pan 1997 / Yu 2014 | Healthy diet | All-cause mortality | 1.02 | [0.21; 4.98] | 0.74 | [0.63; 0.86] | 1.38 | [0.28; 6.76] |
| TOHP II 1997 / Cohen 2008 | Low-sodium | All-cause mortality | 0.67 | [0.36; 1.25] | 1.06 | [0.99; 1.14] | 0.63 | [0.34; 1.18] |
| **Random effects model** | | | | | | | **0.93** | **[0.70; 1.24]** |
| **Prediction interval** | | | | | | | | **[0.42; 2.04]** |
| Heterogeneity: $I^2 = 14\%$, $\tau^2 = 0.0123$, $p = 0.32$ | | | | | | | | |
| ***Cardiovascular disease*** | | | | | | | | |
| Burr 1989 / Guasch-Ferre 2015 | PUFA | Major cardiovascular events | 0.99 | [0.87; 1.12] | 0.68 | [0.48; 0.96] | 1.46 | [1.01; 2.11] |
| Estruch 2018 / Bao 2013 | Nuts | Coronary heart disease | 0.79 | [0.55; 1.14] | 0.81 | [0.75; 0.88] | 0.98 | [0.67; 1.42] |
| Estruch 2018 / Bernstein 2012 | Nuts | Stroke | 0.62 | [0.44; 0.87] | 0.90 | [0.79; 1.02] | 0.69 | [0.48; 0.99] |
| Estruch 2018 / Buckland 2009 | Mediterranean diet | Cardiovascular disease | 0.81 | [0.64; 1.02] | 0.60 | [0.47; 0.77] | 1.35 | [0.96; 1.90] |
| Howard 2006 / Leosdottir 2005 | Low-fat diet | Cardiovascular disease mortality | 0.99 | [0.81; 1.20] | 1.82 | [0.85; 3.85] | 0.54 | [0.25; 1.19] |
| Hsia 2007 / Yang 2016 | Calcium | Cardiovascular disease mortality | 1.01 | [0.79; 1.29] | 0.85 | [0.78; 0.92] | 1.19 | [0.92; 1.54] |
| Sesso 2012 / Bailey 2015 | Multivitamins/Minerals | Cardiovascular disease mortality | 0.95 | [0.83; 1.09] | 0.65 | [0.49; 0.85] | 1.46 | [1.07; 1.99] |
| Sesso 2012 / Rautiainen 2016 | Multivitamins/Minerals | Coronary heart disease | 0.93 | [0.79; 1.09] | 0.88 | [0.74; 1.04] | 1.06 | [0.84; 1.34] |
| Sesso 2012 / Rautiainen 2016 | Multivitamins/Minerals | Stroke | 1.06 | [0.89; 1.25] | 0.94 | [0.79; 1.13] | 1.13 | [0.88; 1.44] |
| Whelton 1998 / Cohen 2008 | Low-sodium | Cardiovascular disease | 0.78 | [0.52; 1.18] | 1.14 | [0.99; 1.30] | 0.68 | [0.44; 1.05] |
| **Random effects model** | | | | | | | **1.06** | **[0.87; 1.27]** |
| **Prediction interval** | | | | | | | | **[0.58; 1.93]** |
| Heterogeneity: $I^2 = 59\%$, $\tau^2 = 0.0597$, $p < 0.01$ | | | | | | | | |
| ***Endocrine / metabolic disease*** | | | | | | | | |
| Baron 2015 / Ferraro 2017 | Vitamin D | Nephrolithiasis | 0.68 | [0.38; 1.21] | 1.10 | [0.99; 1.22] | 0.62 | [0.34; 1.11] |
| Pan 1997 / Chiuve 2012 | Healthy diet | Type 2 diabetes | 0.65 | [0.52; 0.81] | 0.82 | [0.76; 0.89] | 0.79 | [0.63; 1.00] |
| Riggs 1998 / Curhan 1997 | Calcium | Nephrolithiasis | 0.33 | [0.01; 7.96] | 1.59 | [0.95; 2.64] | 0.21 | [0.01; 6.10] |
| Salas-Salvadó 2008 / Tortosa 2007 | Mediterranean diet | Metabolic syndrome | 0.90 | [0.82; 1.00] | 0.39 | [0.14; 0.81] | 2.32 | [0.96; 5.61] |
| Salas-Salvado 2014 / InterAct 2011 | Mediterranean diet | Type 2 diabetes | 0.69 | [0.53; 0.91] | 0.88 | [0.79; 0.97] | 0.78 | [0.59; 1.05] |
| Salas-Salvado 2014 / InterAct 2011 | Olive oil | Type 2 diabetes | 0.89 | [0.82; 0.96] | 0.88 | [0.81; 0.95] | 1.01 | [0.90; 1.13] |
| Salas-Salvado 2014 / Pan 2013 | Nuts | Type 2 diabetes | 0.86 | [0.68; 1.08] | 0.88 | [0.83; 0.94] | 0.98 | [0.77; 1.24] |
| **Random effects model** | | | | | | | **0.90** | **[0.71; 1.14]** |
| **Prediction interval** | | | | | | | | **[0.46; 1.76]** |
| Heterogeneity: $I^2 = 50\%$, $\tau^2 = 0.0540$, $p = 0.06$ | | | | | | | | |
| ***Fractures*** | | | | | | | | |
| Jackson 2006 / Prentice 2013 | Calcium | Fractures | 0.97 | [0.92; 1.03] | 1.07 | [1.01; 1.14] | 0.91 | [0.83; 0.98] |
| ***Pregnancy outcomes*** | | | | | | | | |
| Brough 2010 / Catov 2011 | Multivitamins | Preterm birth | 1.09 | [0.43; 2.77] | 0.89 | [0.80; 0.99] | 1.22 | [0.48; 3.13] |
| Charles 2005 / Wen 2016 | Folic acid | Pre-eclampsia | 0.61 | [0.34; 1.08] | 0.59 | [0.34; 1.02] | 1.04 | [0.47; 2.31] |
| Christian 2003 / Wang 2016 | Folic acid | Low birthweight | 0.96 | [0.85; 1.09] | 0.35 | [0.05; 2.49] | 2.74 | [0.39; 19.43] |
| Czeizel 1994 / Milunsky 1989 | Folic acid | Neural tube defect | 0.07 | [0.00; 1.32] | 0.29 | [0.15; 0.55] | 0.26 | [0.01; 4.89] |
| Czeizel 1998 / Czeizel 2004 | Folic acid | Congenital heart defects | 0.48 | [0.23; 1.03] | 0.60 | [0.38; 0.96] | 0.80 | [0.33; 1.93] |
| Hollis 2011 / Haugen 2009 | Vitamin D | Pre-eclampsia | 0.50 | [0.20; 1.23] | 0.68 | [0.47; 0.99] | 0.74 | [0.28; 1.97] |
| Kirke 1992 / Nohr 2014 | Multivitamins | Stillbirth | 2.43 | [0.12; 50.05] | 0.82 | [0.62; 1.10] | 2.96 | [0.14; 61.35] |
| Merchant 2005 / Timmermans 2011 | Folic acid | Gestational hypertension | 0.63 | [0.42; 0.94] | 1.21 | [0.92; 1.57] | 0.52 | [0.32; 0.85] |
| Moses 2014 / Gresham 2016 | Healthy diet | Small for gestational age | 0.86 | [0.48; 1.54] | 0.42 | [0.11; 1.09] | 2.05 | [0.57; 7.42] |
| Walsh 2012 / Hillesund 2014 | Healthy diet | Preterm birth | 0.39 | [0.10; 1.46] | 0.91 | [0.80; 1.03] | 0.43 | [0.11; 1.65] |
| Walsh 2012 / Tobias 2012 | Mediterranean diet | Gestational diabetes | 0.71 | [0.35; 1.45] | 0.76 | [0.60; 0.95] | 0.93 | [0.44; 1.97] |
| **Random effects model** | | | | | | | **0.79** | **[0.60; 1.05]** |
| **Prediction interval** | | | | | | | | **[0.57; 1.10]** |
| Heterogeneity: $I^2 = 1\%$, $\tau^2 = 0.0012$, $p = 0.43$ | | | | | | | | |
| ***Cancer*** | | | | | | | | |
| Armitage 2010 / Slatore 2008 | Folic acid | Lung cancer | 0.95 | [0.74; 1.22] | 0.92 | [0.40; 2.11] | 1.04 | [0.44; 2.47] |
| Brunner 2011 / Robien 2007 | Vitamin D | Breast Cancer | 0.96 | [0.85; 1.09] | 0.96 | [0.91; 1.01] | 1.00 | [0.87; 1.14] |
| de Lorgeril 1998 / Lassale 2016 | Mediterranean diet | Cancer mortality | 0.75 | [0.17; 3.33] | 0.82 | [0.77; 0.88] | 0.91 | [0.21; 4.05] |
| Gaziano 2009 / Kirsh 2006 | Vitamin C | Prostate cancer | 1.01 | [0.90; 1.14] | 1.01 | [0.94; 1.09] | 1.00 | [0.87; 1.15] |
| Gaziano 2009 / Rodriguez 2004 | Vitamin E | Prostate cancer | 0.96 | [0.85; 1.08] | 1.00 | [0.97; 1.03] | 0.96 | [0.85; 1.09] |
| Heinonen 1998 / Kirsh 2006 | ß-carotene | Prostate cancer | 1.24 | [0.96; 1.59] | 0.55 | [0.10; 2.92] | 2.24 | [0.41; 12.36] |
| Karp 2013 / Dong 2008 | Selenium | Oesophageal cancer | 1.50 | [0.06; 36.86] | 0.02 | [0.00; 1.94] | 75.00 | [0.28; 20175.93] |
| Lin 2009 / Cui 2008 | Vitamin C | Breast cancer | 1.11 | [0.87; 1.41] | 1.08 | [1.02; 1.14] | 1.03 | [0.80; 1.32] |
| Lin 2009 / Egnell 2017 | Vitamin C | Colorectal cancer | 0.76 | [0.42; 1.38] | 0.00 | [0.00; 11.67] | 760.00 | [0.06; 9833094.45] |
| Lin 2009 / Slatore 2008 | Vitamin C | Lung cancer | 1.84 | [1.14; 2.97] | 0.95 | [0.74; 1.21] | 1.94 | [1.13; 3.32] |
| Lippman 2009 / Hansen 2013 | Selenium | Colorectal cancer | 1.04 | [0.73; 1.48] | 0.52 | [0.32; 0.86] | 2.00 | [1.09; 3.67] |
| Lippman 2009 / Peters 2008 | Selenium | Prostate cancer | 1.03 | [0.90; 1.18] | 0.83 | [0.37; 1.89] | 1.24 | [0.54; 2.84] |
| Meyer 2005 / Lawson 2007 | Multivitamins | Prostate cancer | 0.90 | [0.62; 1.33] | 1.03 | [0.99; 1.07] | 0.87 | [0.60; 1.28] |
| Schatzkin 2000 / Michels 2005 | Fiber | Colorectal cancer | 2.47 | [0.78; 7.85] | 0.94 | [0.74; 1.20] | 2.63 | [0.81; 8.55] |
| Toledo 2018 / Buckland 2013 | Mediterranean diet | Breast cancer | 0.41 | [0.19; 0.86] | 0.94 | [0.88; 1.00] | 0.44 | [0.20; 0.93] |
| Zhang 2008 / Maruti 2009 | Folic acid | Breast cancer | 0.83 | [0.61; 1.14] | 0.91 | [0.35; 2.36] | 0.91 | [0.33; 2.50] |
| Zhang 2008 / Skinner 2004 | Folic acid | Pancreatic cancer | 1.50 | [0.42; 5.31] | 5.86 | [0.25; 139.27] | 0.26 | [0.01; 7.72] |
| Zhang 2008 / Zschäbitz 2013 | Folic acid | Colorectal cancer | 0.82 | [0.44; 1.52] | 0.89 | [0.25; 3.20] | 0.92 | [0.22; 3.79] |
| **Random effects model** | | | | | | | **1.08** | **[0.89; 1.31]** |
| **Prediction interval** | | | | | | | | **[0.61; 1.91]** |
| Heterogeneity: $I^2 = 32\%$, $\tau^2 = 0.0637$, $p = 0.10$ | | | | | | | | |
| ***Eye disease*** | | | | | | | | |
| Maraini 2008 / Milton 2006 | Multivitamins/Minerals | Nuclear cataract | 0.66 | [0.50; 0.88] | 0.75 | [0.61; 0.91] | 0.88 | [0.62; 1.24] |
| Maraini 2008 / Milton 2006 | Multivitamins/Minerals | Cortical cataract | 0.78 | [0.60; 1.02] | 0.91 | [0.68; 1.22] | 0.86 | [0.58; 1.27] |
| Maraini 2008 / Milton 2006 | Multivitamins/Minerals | Posterior subcapsular cataract | 2.00 | [1.35; 2.98] | 1.02 | [0.77; 1.36] | 1.96 | [1.20; 3.19] |
| **Random effects model** | | | | | | | **1.12** | **[0.66; 1.88]** |
| **Prediction interval** | | | | | | | | **[0.00; 552.31]** |
| Heterogeneity: $I^2 = 76\%$, $\tau^2 = 0.1679$, $p = 0.02$ | | | | | | | | |
| Test for subgroup differences: $\chi^2_6 = 6.11$, df = 6 ($p = 0.41$) | | | | | | | | |

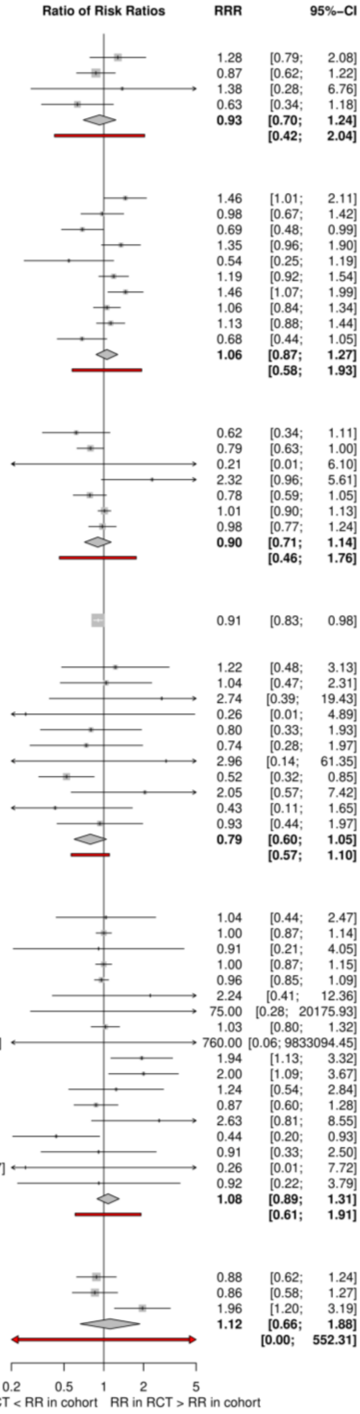


CI: confidence interval; PUFA: polyunsaturated fatty acid; RCT: randomised controlled trial; RR: risk ratio; RRR: ratio of risk ratios; TOHP II: Trials of Hypertension Prevention, Phase II

**Figure S9** Forest plot of the comparison between study design pairs with binary outcomes / subgroup analysis by PI/ECO similarity

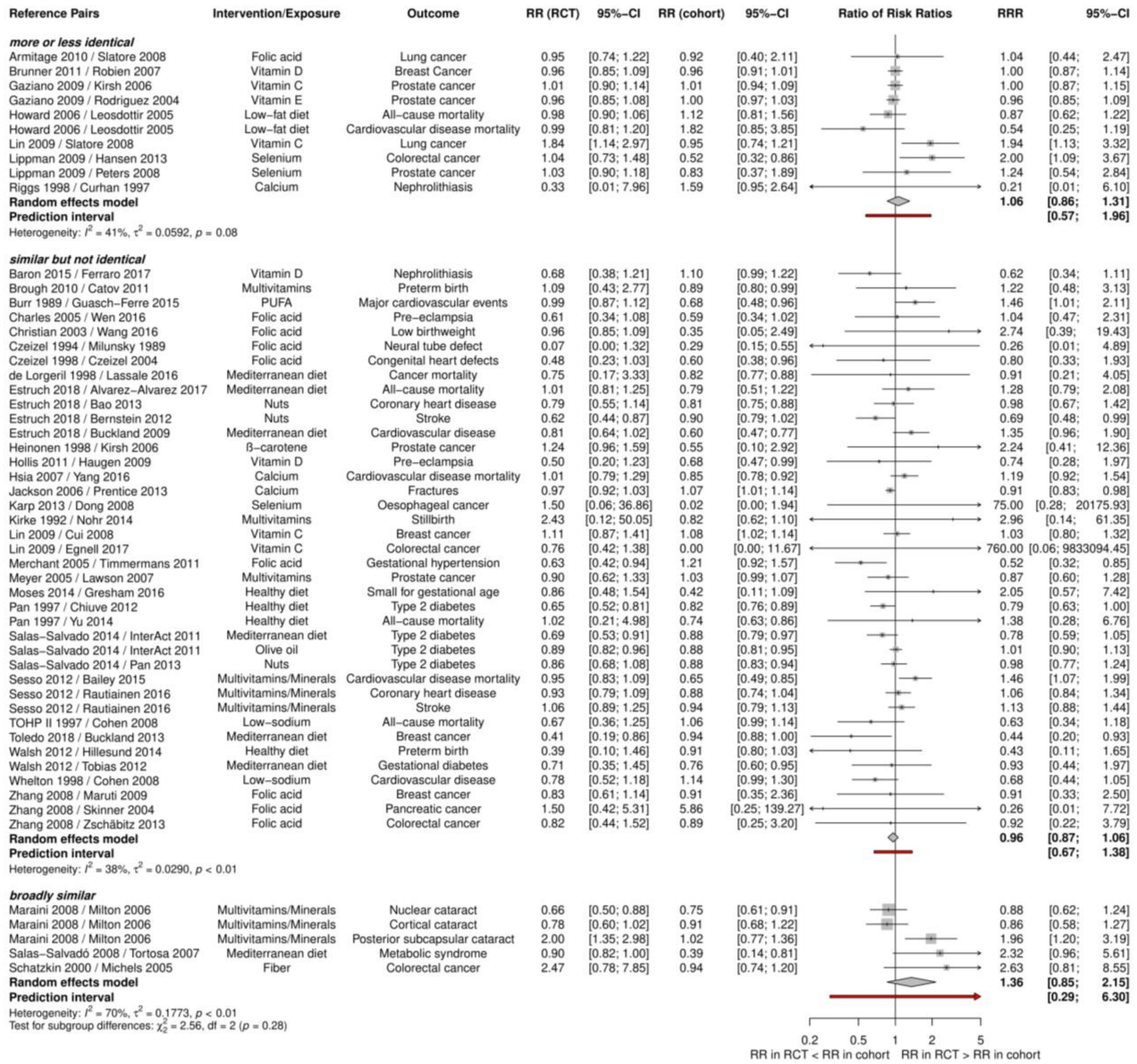


CI: confidence interval; PUFA: polyunsaturated fatty acid; RCT: randomised controlled trial; RR: risk ratio; RRR: ratio of risk ratios; TOHP II: Trials of Hypertension Prevention, Phase II

**Figure S10** Forest plot of the comparison between study design pairs with binary outcomes / subgroup analysis by risk of bias rating

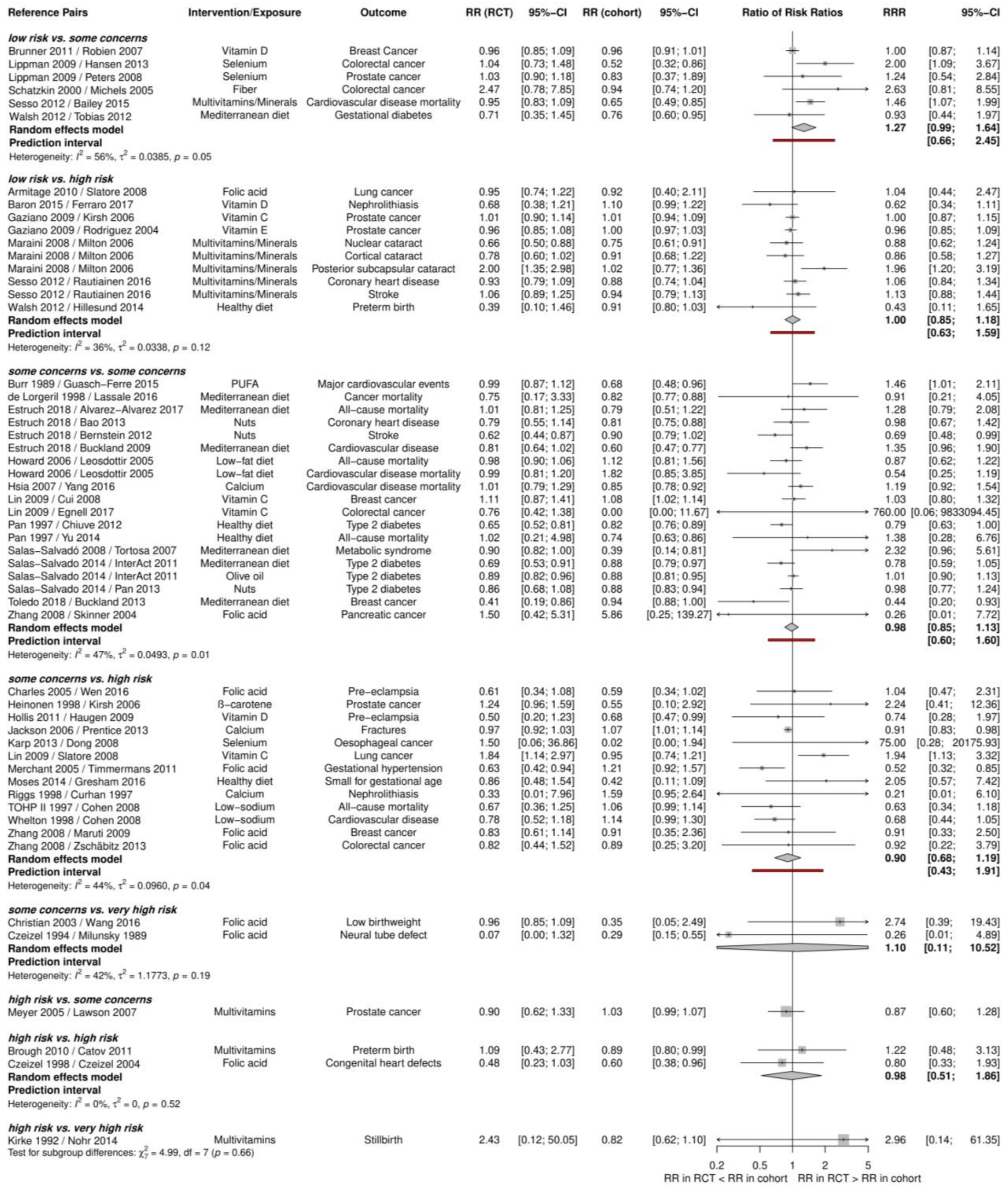


CI: confidence interval; PUFA: polyunsaturated fatty acid; RCT: randomised controlled trial; RR: risk ratio; RRR: ratio of risk ratios; TOHP II: Trials of Hypertension Prevention, Phase II

**Figure S11** Forest plot of the comparison between study design pairs with binary outcomes / sensitivity analysis excluding pairs with high risk of bias rating

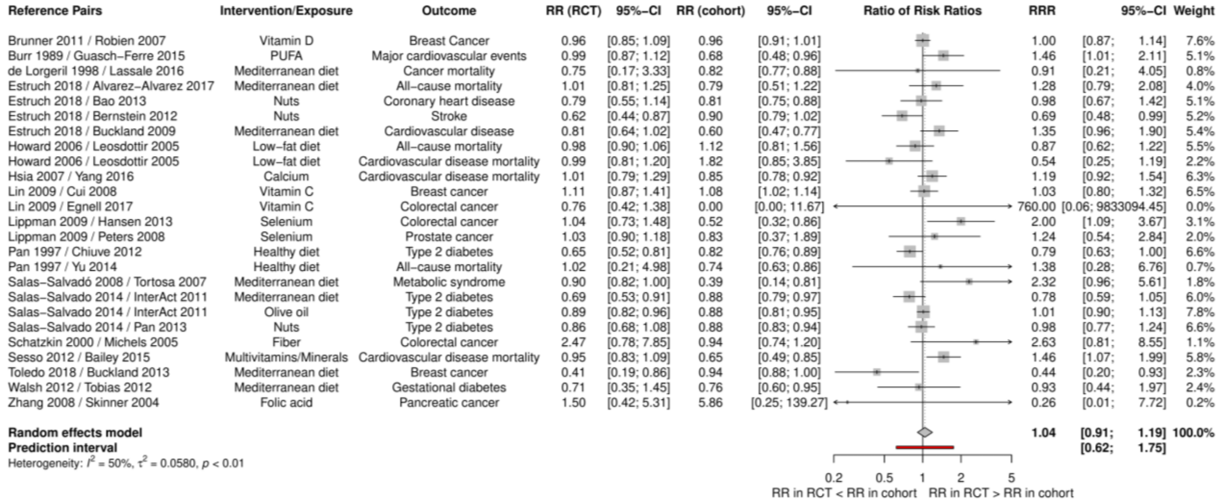


CI: confidence interval; PUFA: polyunsaturated fatty acid; RCT: randomised controlled trial; RR: risk ratio

**Figure S12** Forest plot of the comparison between study design pairs with binary outcomes / sensitivity analysis including each RCT only once* for each outcome

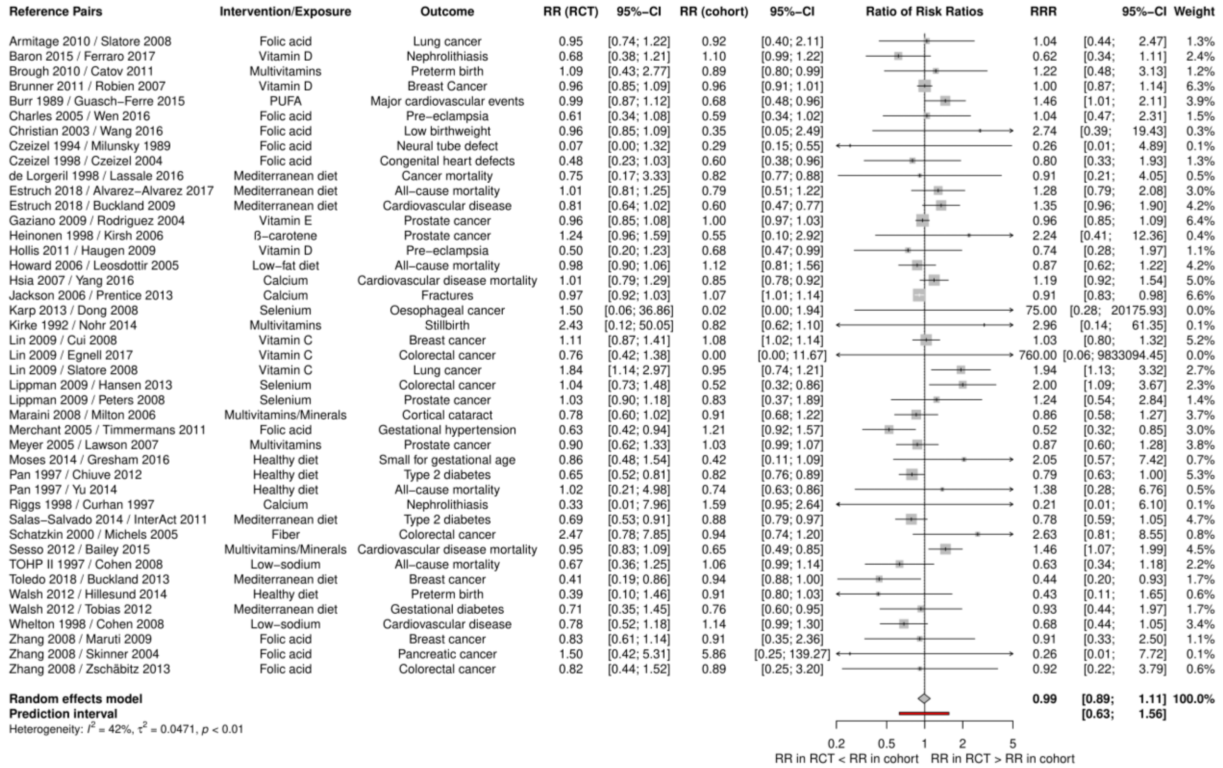


CI: confidence interval; PUFA: polyunsaturated fatty acid; RCT: randomised controlled trial; RR: risk ratio; TOHP II: Trials of Hypertension Prevention, Phase II

* We opted for the comparison with the highest number of included participants and cases in the RCT.

**Figure S13** Forest plot of the comparison between study design pairs with binary outcomes / sensitivity analysis including only RCTs with largest sample size

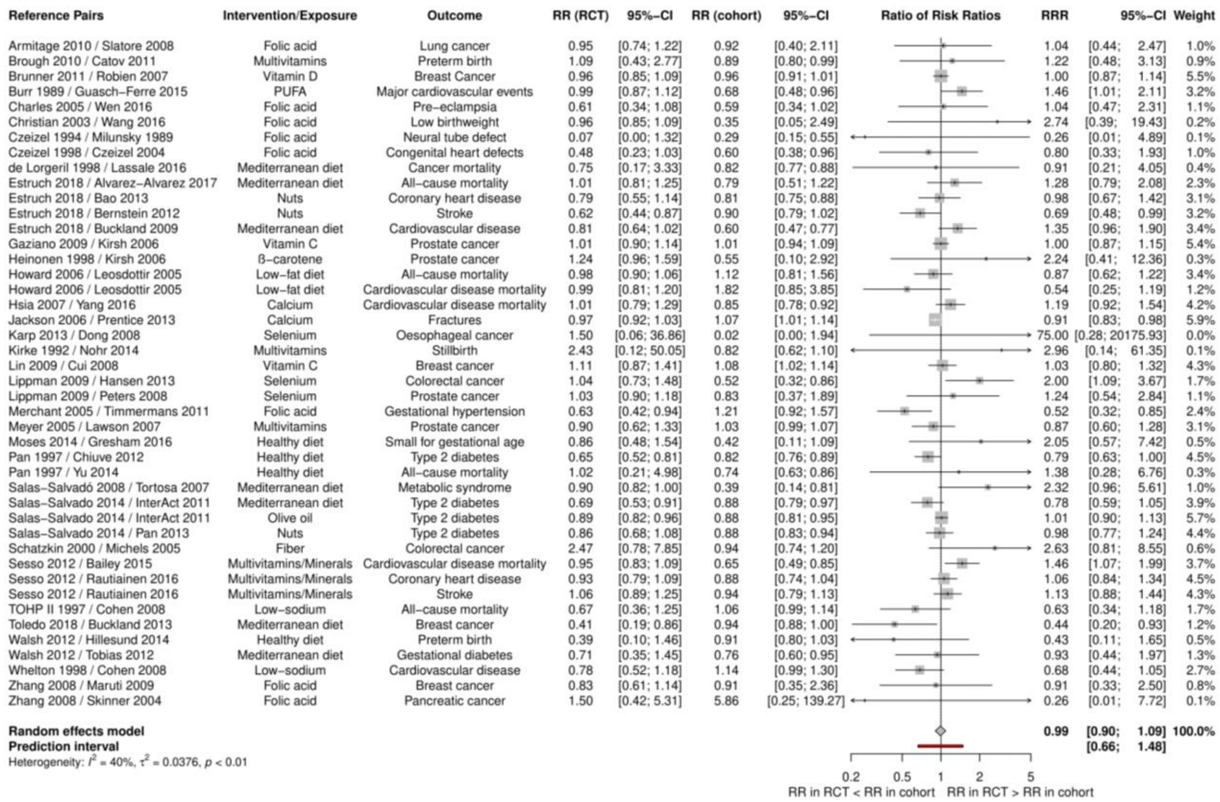


| Reference Pairs | Intervention/Exposure | Outcome | RR (RCT) | 95%-CI | RR (cohort) | 95%-CI | RRR | 95%-CI | Weight |
|---|---|---|---|---|---|---|---|---|---|
| Armitage 2010 / Slatore 2008 | Folic acid | Lung cancer | 0.95 | [0.74; 1.22] | 0.92 | [0.40; 2.11] | 1.04 | [0.44; 2.47] | 1.0% |
| Brough 2010 / Catov 2011 | Multivitamins | Preterm birth | 1.09 | [0.43; 2.77] | 0.89 | [0.80; 0.99] | 1.22 | [0.48; 3.13] | 0.9% |
| Brunner 2011 / Robien 2007 | Vitamin D | Breast Cancer | 0.96 | [0.85; 1.09] | 0.96 | [0.91; 1.01] | 1.00 | [0.87; 1.14] | 5.5% |
| Burr 1989 / Guasch–Ferre 2015 | PUFA | Major cardiovascular events | 0.99 | [0.87; 1.12] | 0.68 | [0.48; 0.96] | 1.46 | [1.01; 2.11] | 3.2% |
| Charles 2005 / Wen 2016 | Folic acid | Pre–eclampsia | 0.61 | [0.34; 1.08] | 0.59 | [0.34; 1.02] | 1.04 | [0.47; 2.31] | 1.1% |
| Christian 2003 / Wang 2016 | Folic acid | Low birthweight | 0.96 | [0.85; 1.09] | 0.35 | [0.05; 2.49] | 2.74 | [0.39; 19.43] | 0.2% |
| Czeizel 1994 / Milunsky 1989 | Folic acid | Neural tube defect | 0.07 | [0.00; 1.32] | 0.29 | [0.15; 0.55] | 0.26 | [0.01; 4.89] | 0.1% |
| Czeizel 1998 / Czeizel 2004 | Folic acid | Congenital heart defects | 0.48 | [0.23; 1.03] | 0.60 | [0.38; 0.96] | 0.80 | [0.33; 1.93] | 1.0% |
| de Lorgeril 1998 / Lassale 2016 | Mediterranean diet | Cancer mortality | 0.75 | [0.17; 3.33] | 0.82 | [0.77; 0.88] | 0.91 | [0.21; 4.05] | 0.4% |
| Estruch 2018 / Alvarez–Alvarez 2017 | Mediterranean diet | All–cause mortality | 1.01 | [0.81; 1.25] | 0.79 | [0.51; 1.22] | 1.28 | [0.79; 2.08] | 2.3% |
| Estruch 2018 / Bao 2013 | Nuts | Coronary heart disease | 0.79 | [0.55; 1.14] | 0.81 | [0.75; 0.88] | 0.98 | [0.67; 1.42] | 3.1% |
| Estruch 2018 / Bernstein 2012 | Nuts | Stroke | 0.62 | [0.44; 0.87] | 0.90 | [0.79; 1.02] | 0.69 | [0.48; 0.99] | 3.2% |
| Estruch 2018 / Buckland 2009 | Mediterranean diet | Cardiovascular disease | 0.81 | [0.64; 1.02] | 0.60 | [0.47; 0.77] | 1.35 | [0.96; 1.90] | 3.4% |
| Gaziano 2009 / Kirsh 2006 | Vitamin C | Prostate cancer | 1.01 | [0.90; 1.14] | 1.01 | [0.94; 1.09] | 1.00 | [0.87; 1.15] | 5.4% |
| Heinonen 1998 / Kirsh 2006 | ß–carotene | Prostate cancer | 1.24 | [0.96; 1.59] | 0.55 | [0.10; 2.92] | 2.24 | [0.41; 12.36] | 0.3% |
| Howard 2006 / Leosdottir 2005 | Low–fat diet | All–cause mortality | 0.98 | [0.90; 1.06] | 1.12 | [0.81; 1.56] | 0.87 | [0.62; 1.22] | 3.4% |
| Howard 2006 / Leosdottir 2005 | Low–fat diet | Cardiovascular disease mortality | 0.99 | [0.81; 1.20] | 1.82 | [0.85; 3.85] | 0.54 | [0.25; 1.19] | 1.2% |
| Hsia 2007 / Yang 2016 | Calcium | Cardiovascular disease mortality | 1.01 | [0.79; 1.29] | 0.85 | [0.78; 0.92] | 1.19 | [0.92; 1.54] | 4.2% |
| Jackson 2006 / Prentice 2013 | Calcium | Fractures | 0.97 | [0.92; 1.03] | 1.07 | [1.01; 1.14] | 0.91 | [0.83; 0.98] | 5.9% |
| Karp 2013 / Dong 2008 | Selenium | Oesophageal cancer | 1.50 | [0.06; 36.86] | 0.02 | [0.00; 1.94] | 75.00 | [0.28; 20175.93] | 0.0% |
| Kirke 1992 / Nohr 2014 | Multivitamins | Stillbirth | 2.43 | [0.12; 50.05] | 0.82 | [0.62; 1.10] | 2.96 | [0.14; 61.35] | 0.1% |
| Lin 2009 / Cui 2008 | Vitamin C | Breast cancer | 1.11 | [0.87; 1.41] | 1.08 | [1.02; 1.14] | 1.03 | [0.80; 1.32] | 4.3% |
| Lippman 2009 / Hansen 2013 | Selenium | Colorectal cancer | 1.04 | [0.73; 1.48] | 0.52 | [0.32; 0.86] | 2.00 | [1.09; 3.67] | 1.7% |
| Lippman 2009 / Peters 2008 | Selenium | Prostate cancer | 1.03 | [0.90; 1.18] | 0.83 | [0.37; 1.89] | 1.24 | [0.54; 2.84] | 1.1% |
| Merchant 2005 / Timmermans 2011 | Folic acid | Gestational hypertension | 0.63 | [0.42; 0.94] | 1.21 | [0.92; 1.57] | 0.52 | [0.32; 0.85] | 2.4% |
| Meyer 2005 / Lawson 2007 | Multivitamins | Prostate cancer | 0.90 | [0.62; 1.33] | 1.03 | [0.99; 1.07] | 0.87 | [0.60; 1.28] | 3.1% |
| Moses 2014 / Gresham 2016 | Healthy diet | Small for gestational age | 0.86 | [0.48; 1.54] | 0.42 | [0.11; 1.09] | 2.05 | [0.57; 7.42] | 0.5% |
| Pan 1997 / Chiuve 2012 | Healthy diet | Type 2 diabetes | 0.65 | [0.52; 0.81] | 0.82 | [0.76; 0.89] | 0.79 | [0.63; 1.00] | 4.5% |
| Pan 1997 / Yu 2014 | Healthy diet | All–cause mortality | 1.02 | [0.21; 4.98] | 0.74 | [0.63; 0.86] | 1.38 | [0.28; 6.76] | 0.3% |
| Salas–Salvadó 2008 / Tortosa 2007 | Mediterranean diet | Metabolic syndrome | 0.90 | [0.82; 1.00] | 0.39 | [0.14; 0.81] | 2.32 | [0.96; 5.61] | 1.0% |
| Salas–Salvado 2014 / InterAct 2011 | Mediterranean diet | Type 2 diabetes | 0.69 | [0.53; 0.91] | 0.88 | [0.79; 0.97] | 0.78 | [0.59; 1.05] | 3.9% |
| Salas–Salvado 2014 / InterAct 2011 | Olive oil | Type 2 diabetes | 0.89 | [0.82; 0.96] | 0.88 | [0.81; 0.95] | 1.01 | [0.90; 1.13] | 5.7% |
| Salas–Salvado 2014 / Pan 2013 | Nuts | Type 2 diabetes | 0.86 | [0.68; 1.08] | 0.88 | [0.83; 0.94] | 0.98 | [0.77; 1.24] | 4.4% |
| Schatzkin 2000 / Michels 2005 | Fiber | Colorectal cancer | 2.47 | [0.78; 7.85] | 0.94 | [0.74; 1.20] | 2.63 | [0.81; 8.55] | 0.6% |
| Sesso 2012 / Bailey 2015 | Multivitamins/Minerals | Cardiovascular disease mortality | 0.95 | [0.83; 1.09] | 0.65 | [0.49; 0.85] | 1.46 | [1.07; 1.99] | 3.7% |
| Sesso 2012 / Rautiainen 2016 | Multivitamins/Minerals | Coronary heart disease | 0.93 | [0.79; 1.09] | 0.88 | [0.74; 1.04] | 1.06 | [0.84; 1.34] | 4.5% |
| Sesso 2012 / Rautiainen 2016 | Multivitamins/Minerals | Stroke | 1.06 | [0.89; 1.25] | 0.94 | [0.79; 1.13] | 1.13 | [0.88; 1.44] | 4.3% |
| TOHP II 1997 / Cohen 2008 | Low–sodium | All–cause mortality | 0.67 | [0.36; 1.25] | 1.06 | [0.99; 1.14] | 0.63 | [0.34; 1.18] | 1.7% |
| Toledo 2018 / Buckland 2013 | Mediterranean diet | Breast cancer | 0.41 | [0.19; 0.86] | 0.94 | [0.88; 1.00] | 0.44 | [0.20; 0.93] | 1.2% |
| Walsh 2012 / Hillesund 2014 | Healthy diet | Preterm birth | 0.39 | [0.10; 1.46] | 0.91 | [0.80; 1.03] | 0.43 | [0.11; 1.65] | 0.5% |
| Walsh 2012 / Tobias 2012 | Mediterranean diet | Gestational diabetes | 0.71 | [0.35; 1.45] | 0.76 | [0.60; 0.95] | 0.93 | [0.44; 1.97] | 1.3% |
| Whelton 1998 / Cohen 2008 | Low–sodium | Cardiovascular disease | 0.78 | [0.52; 1.18] | 1.14 | [0.99; 1.30] | 0.68 | [0.44; 1.05] | 2.7% |
| Zhang 2008 / Maruti 2009 | Folic acid | Breast cancer | 0.83 | [0.61; 1.14] | 0.91 | [0.35; 2.36] | 0.91 | [0.33; 2.50] | 0.8% |
| Zhang 2008 / Skinner 2004 | Folic acid | Pancreatic cancer | 1.50 | [0.42; 5.31] | 5.86 | [0.25; 139.27] | 0.26 | [0.01; 7.72] | 0.1% |
| **Random effects model** | | | | | | | **0.99** | **[0.90; 1.09]** | **100.0%** |
| **Prediction interval** | | | | | | | | **[0.66; 1.48]** | |

Heterogeneity: $I^2 = 40\%$, $\tau^2 = 0.0376$, $p < 0.01$

CI: confidence interval; PUFA: polyunsaturated fatty acid; RCT: randomised controlled trial; RR: risk ratio;
